# Identification of a Novel Drug Lead That Inhibits HCV Infection and Cell-to-Cell Transmission by Targeting the HCV E2 Glycoprotein

**DOI:** 10.1371/journal.pone.0111333

**Published:** 2014-10-30

**Authors:** Reem R. Al Olaby, Laurence Cocquerel, Adam Zemla, Laure Saas, Jean Dubuisson, Jost Vielmetter, Joseph Marcotrigiano, Abdul Ghafoor Khan, Felipe Vences Catalan, Alexander L. Perryman, Joel S. Freundlich, Stefano Forli, Shoshana Levy, Rod Balhorn, Hassan M. Azzazy

**Affiliations:** 1 Department of Chemistry, The American University in Cairo, New Cairo, Egypt; 2 Center for Infection and Immunity of Lille, CNRS-UMR8204/Inserm-U1019, Pasteur Institute of Lille, University of Lille North of France, Lille, France; 3 Pathogen Bioinformatics, Lawrence Livermore National Laboratory, Livermore, CA, United States of America; 4 Protein Expression Center, Beckman Institute, California Institute of Technology, Pasadena, CA, United States of America; 5 Department of Chemistry and Chemical Biology, Rutgers University, Piscataway, NJ, United States of America; 6 Department of Medicine, Stanford University Medical Center, Stanford, CA, United States of America; 7 Department of Medicine, Division of Infectious Diseases, Center for Emerging & Re-emerging Pathogens, Rutgers University-New Jersey Medical School, Newark, NJ, United States of America; 8 Department of Pharmacology and Physiology, Rutgers University-New Jersey Medical School, Newark, NJ, United States of America; 9 Department of Integrative Structural and Computational Biology, The Scripps Research Institute, La Jolla, CA, United States of America; 10 Department of Applied Science, University of California Davis, Davis, CA, United States of America; University of South Florida College of Medicine, United States of America

## Abstract

Hepatitis C Virus (HCV) infects 200 million individuals worldwide. Although several FDA approved drugs targeting the HCV serine protease and polymerase have shown promising results, there is a need for better drugs that are effective in treating a broader range of HCV genotypes and subtypes without being used in combination with interferon and/or ribavirin. Recently, two crystal structures of the core of the HCV E2 protein (E2c) have been determined, providing structural information that can now be used to target the E2 protein and develop drugs that disrupt the early stages of HCV infection by blocking E2’s interaction with different host factors. Using the E2c structure as a template, we have created a structural model of the E2 protein core (residues 421–645) that contains the three amino acid segments that are not present in either structure. Computational docking of a diverse library of 1,715 small molecules to this model led to the identification of a set of 34 ligands predicted to bind near conserved amino acid residues involved in the HCV E2: CD81 interaction. Surface plasmon resonance detection was used to screen the ligand set for binding to recombinant E2 protein, and the best binders were subsequently tested to identify compounds that inhibit the infection of Huh-7 cells by HCV. One compound, 281816, blocked E2 binding to CD81 and inhibited HCV infection in a genotype-independent manner with IC50’s ranging from 2.2 µM to 4.6 µM. 281816 blocked the early and late steps of cell-free HCV entry and also abrogated the cell-to-cell transmission of HCV. Collectively the results obtained with this new structural model of E2c suggest the development of small molecule inhibitors such as 281816 that target E2 and disrupt its interaction with CD81 may provide a new paradigm for HCV treatment.

## Introduction

Hepatitis C virus (HCV) is a global public health problem [1] in which nearly 85% of affected individuals have acute HCV infections and exhibit no symptoms. In addition, more than three-quarters of these cases will advance to chronic disease, which include liver cirrhosis and liver cancer [2]. The current standard of care treatment for HCV (Peg-interferon/Ribavirin, PR) can cause deleterious side effects, and a sustained virologic response (SVR) is achieved in less than 50% of genotype-1 patients [3]. The FDA approved protease inhibitors Telaprevir (TVR) and Boceprevir (BOC) have been shown to provide higher SVR rates in genotype 1 patients [3], [4] when each is combined with PR. However the poor safety profile of TVR and BOC reported in the Week 16 analysis of the French Early Access Program suggest there is still a need for better HCV drugs [5]. The two most recent FDA approvals have been for the oral drugs Simeprevir and Sofosbuvir, inhibitors that target the HCV NS3/4A protease and polymerase, respectively [6]. Semiprevir, which needs to be administered with Ribavirin and Peg-interferon, has a number of undesirable side effects [7]. The efficacy of Semiprevir has also been shown to be diminished significantly, due to viral breakthrough (HCV RNA rebounds and becomes detectable in the patient before treatment is completed), in patients infected by HCV genotypes 4–6 containing the Q80K, R155K and D168E/V polymorphisms in the NS3 protease [7]. Recommendations for the use of Sofosbuvir indicate it should be administered with Ribavirin in HCV genotype 2 and 3 infections and that Peg-Interferon should be included in the treatment when infections involve genotypes 1 and 4. While Sofosbuvir is considered the Holy Grail in HCV treatment by some, it is recommended that treatments be limited to 12 weeks [6]. Its high cost ($1,000 USD/pill) also puts it out of reach of many HCV infected patients. This has led many of the larger pharmaceutical companies to continue developing new drugs that target one or more steps in the HCV life cycle and block virus invasion, processing of the pro-protein or replication of the viral genome.

Since its identification as the first putative receptor for HCV [8], the tetraspanin CD81 has been demonstrated to be a key player in HCV entry [9]. In particular, its large extracellular loop (CD81-LEL) is involved in the binding to the HCV envelope glycoprotein E2 [10], [11]. Zhang et al. [12] elucidated a separate, additional function for CD81 in the HCV life cycle. These studies revealed that CD81-LEL is important for efficient HCV genome replication. In addition, the E2-CD81-LEL interaction has been determined to induce several immuno-modulatory effects such as the production and release of pro-inflammatory cytokine gamma interferon from T-cells. In addition, this interaction has also been shown to down regulate T-cell receptors and suppress the activity of natural killer (NK) cells [13]. Therefore, it is tempting to speculate that blocking the CD81-LEL:HCV E2 interaction might also contribute to arresting disease progression to liver cirrhosis.

Following the discovery of the E2 glycoprotein’s role in HCV infection and disease progression, several approaches have been used to attempt to develop anti-HCV drugs and vaccines that target the HCV E2 glycoprotein [14]–[17] located on the surface of viral particles. These efforts have had to deal with challenges that relate to the genomic diversity and heterogeneity of HCV, limitations in animal models used to test vaccines and drugs, and the lack of a resolved crystal structure for the HCV E2 glycoprotein. Recently, two crystal structures have been reported for the core ectodomain of the HCV E2 protein [18], [19]. Kong et al. [18] obtained the structure of amino acid residues 384–746 (E2c) by designing and expressing 41 soluble HCV E2 constructs and selecting 15 to screen against E2-specific Fab fragments in crystallization trials. Using a combination of x-ray crystallography and negative stain-electron microscopy, Kong et al. [18] discovered the structures they obtained for E2 were globular and very different from the predicted models of E2 that were created using class II fusion protein templates containing three β-sheet domains. Additionally, they were able to identify key CD81-binding residues through mutational studies. Important CD81 binding sites were determined to be in the epitope recognized by the neutralizing antibody AR3C, along one side of the β-sandwich (an isolated region of the CD81-binding loop) and a front layer consisting of loops, short helices and β-sheets [18]–[20]. AR3C was also found to cross-neutralize HCV genotypes by blocking CD81 binding to HCV E2 [21]. A second structure was reported for E2c (amino acid residues 492–649) by Khan et al. [19]. This new structure, which was obtained by crystallizing E2c in complex with a Fab fragment of the mouse monoclonal antibody 2A12, is very similar to the previously reported structure. In addition to providing a second structure for the E2 core from a different HCV genotype (2a), new information was also reported on the accessibility of the E2 core amino acids within the structure using a combination of limited proteolytic degradation and deuterium exchange.

Despite the advances that have been made in the field of HCV drug development, our current drugs offer little protection against the emergence of genetic variants (escape variants) of HCV – a feature of HCV biology that complicates both drug and vaccine development. Drugs that target only one step in the HCV life cycle will be the least effective in treating patients that become infected with these emerging variants. The FDA approved drugs for HCV are good examples, as they are only effective against a subset of genotypes. In an effort to identify a suitable drug candidate that targets the majority of the existing HCV genotypes, we created an HCV E2 homology model based on the new HCV E2 core crystal structure reported by Kong et al. [18] that contains three peptide segments that were not present in the reported structure, and we have used this model to identify small molecule drug leads that target highly conserved sites on the HCV E2 glycoprotein located within the region bound by CD81. AutoDock was used to perform virtual screening runs against 1,715 small molecules and 34 of the best compounds were tested experimentally using surface plasmon resonance (Biacore T100) to identify a set of small molecules that bind to the recombinant E2 protein. The compounds showing binding activities were then tested for their ability to block HCV infection of Huh-7 cells. One compound, 281816, was found to block infection of the cells by each of the HCV genotypes and subtypes tested (1a, 1b, 2a, 2b, 4a and 6a) in a dose-dependent manner. Experiments with Huh-7 cells have shown that both mechanisms that lead to HCV infection, cell-free and cell-to-cell transmission, are abrogated by 281816. Inhibition of cell-free infection is limited to the viral attachment step, as well as interactions occurring during viral internalization and fusion; 281816 appears to have no effect on post-entry processes.

## Materials and Methods

### Creation of the homology model of E2 used for docking

A crystal structure of E2c deposited in the PDB under a code 4MWF was solved by Kong et al. [18] at a resolution of 2.65 Angstroms. However, upon examination of the structure file prior to docking, the set of reported atom coordinates of the protein was found to be incomplete. In addition to the coordinate file containing structural information for only 171 residues out of the 363 amino acids present in the full-length protein, structural information was missing for several peptide segments or loops within the structural core of the protein. In order to prepare a more complete version of the structure for docking, we have performed several homology modeling and structure analysis tasks using the coordinates of E2c as a template. The final structural model was created using the AS2TS system [21] based on atom coordinates from the PDB chains 4mwf_C and 4mwf_D and extensively manually edited. A structural search for similar fragments in proteins in the PDB that could be used to model missing loop regions was performed using the StralSV algorithm [22], which identifies protein structures that exhibit structural similarities despite low primary amino acid sequence similarity. The side-chain prediction was accomplished using SCWRL [23] when residue-residue correspondences did not match. Residues that were identical in the template and E2 protein were copied from the template onto the model. Potential steric clashes were identified in the unrefined model using a contact-dot algorithm in the MolProbity software package [24], and the constructed model was finished with relaxation using UCSF Chimera [25].

### Virtual screen of the NCI Diversity Set III to the HCV E2 protein model

AutoDock VINA 1.1.2 (VINA) [26] was used to perform a virtual screen of the NCI Diversity Set III against the homology model that was created using the new crystal structure solved by Kong et al. [18] (PDB ID: 4MWF) as a template. The model of the protein was prepared for docking using the MolProbity Server (to add all of the hydrogen atoms and to flip the HIS/ASN/GLN residues if doing so significantly lowered the energy) and AutoDockTools 4.2 (which added the Gasteiger-Marsili charges and merged the non-polar hydrogens onto their respective heavy atoms) [27], [28]. The NCI Diversity Set III library containing 1,715 models of compounds was obtained from the ZINC server (http://zinc.docking.org) [29]. The multi-molecule “mol2” files from ZINC were prepared for docking calculations using Raccoon [30], which added the Gasteiger-Marsili charges, merged the non-polar hydrogen atoms onto their respective heavy atoms, and determined which bonds should be allowed to freely rotate during the calculations, to generate the “pdbqt” docking input format.

Four different, overlapping grid boxes were used in this virtual screen to enable the docking calculations to explore almost the entire surface of this E2 model. Those amino acids missing from the E2c crystal structure whose modeled coordinates were known with the lowest degree of certainty, such as residues E454 and L456–E482 located in the large missing loop and residues G575–L580 and F586–K588 in the two other two missing segments, were not included in the boxes. By defining the boxes to exclude these residues, we were able to minimize the impact of these less accurate parts of the model on ligand docking. Since large grid boxes were used in these calculations, the “exhaustiveness” setting in VINA was increased to 20. Each calculation used 8 CPUs on the Linux cluster at Rutgers University-NJMS. The first box, which included P490, was centered at 38.829, 12.968, −40.958 (x, y, z) and had the following dimensions: 24.0×35.0×30.0 (x, y, z in Angstroms). The second grid box, which included G436, was centered at 48.401, 11.791, −14.449 and had a size of: 32.0×36.0×24.0. The third grid box, which included S528, was centered at 51.644, 25.877, −27.795 and encompassed 30.0×30.0×30.0 Angstroms^3^. The fourth grid box, which was selected to include the side of E2 not covered by the previous three grid boxes, was centered at 57.777, 12.968, −34.067 and enclosed 24.0×35.0×32.0 Angstroms^3^.

The docking outputs generated by VINA were processed and filtered using python scripts from Raccoon2 and Fox [30]. The top-ranked VINA mode from each docking calculation was harvested, and 17 different sets of energetic and interaction-based filters (Table 1) were investigated to harvest the most promising docking results for visual inspection. Different sites have different numbers and arrangements of hydrogen bond donors, hydrogen bond acceptors, and aromatic rings. They also have very different geometries (i.e., van der Waal surfaces and solvent accessibility patterns and percentages). Consequently, several different filters were tested for the docking results against each site in order to harvest a”reasonable” number of docked modes for visual inspection against each site. This is a subjective process, guided by extensive experience with virtual screening. If the same filters are used against each site, then for some sites (or for filters that are not restrictive enough), too many compounds are obtained for visual inspection (i.e., the process is less efficient and a larger number of false positive results are likely to occur). For other sites (or for filters that are too restrictive), an insufficient number of compounds will be obtained for visual inspection. This would increase the chance of missing promising candidates (having too many false negatives) [31]. The following parameters were explored in the filtering process: -e indicates the minimum estimated Free Energy of Binding from the VINA score in kcal/mol, -l is the minimum ligand efficiency value in kcal/mol/heavy atom, -S is the minimum number of hydrogen bonds between the ligand and target, and -H indicates that the ligand had to form a hydrogen bond with either a backbone amino group (::N) or a backbone carbonyl oxygen (::O) of any residue in that grid box.

**Table 1:** Energetic and interaction-based filters used to harvest the most promising results from the ligand docking runs.

Filter	Parameter Set
1	-e −6.5 -l −0.29 -S 3
2	-e −7.0 -l −0.29 -S 3
3	-e −7.5 -l −0.29 -S 3
4	-e −8.0 -l −0.29 -S 3
5	-e −7.0 -l −0.29 -S 4
6	-e −7.5 -l −0.29 -S 4
7	-e −8.0 -l −0.29 -S 4
8	-e −6.5 -l −0.29 -S 3 -H ::N
9	-e −6.5 -l −0.29 -S 3 -H ::O
10	-e −7.0 -l −0.29 -S 3 -H ::N
11	-e −7.0 -l −0.29 -S 3 -H ::O
12	-e −7.0 -l −0.29 -S 4 -H ::N
13	-e −7.0 -l −0.29 -S 4 -H ::O
14	-e −7.0 -S 3 -H ::N
15	-e −7.0 -S 3 -H ::O
16	-e −7.0 -S 4 -H ::N
17	-e −7.0 -S 4 -H ::O

For the results with grid box 1, filters 12 and 13 each harvested 70 and 51 compounds, respectively. Those filtered sets were pooled together to form a set of 96 unique compounds for visual inspection. Filters 14 (which harvested 11 compounds), 15 (which harvested 21 compounds), and 1 (which harvested 34 compounds) were pooled together from the results with grid box 2, in order to identify 52 compounds for visual inspection. Similarly, for the results with grid box 3, filters 1 (which harvested 25 compounds), 14 (which identified 20 candidates), and 15 (which harvested 13 compounds) were pooled to obtain 34 compounds for visual inspection. To identify candidates in the results with grid box 4, filters 1 (which harvested 26 compounds), 14 (which harvested 19 compounds), and 15 (which harvested 14 compounds) were pooled to obtain 42 compounds.

These four different pools of potentially promising compounds were then visually inspected to select the ligands to be tested experimentally for binding to recombinant E2 protein. Both the structure of the compound and the nature of its predicted interaction with the protein were examined. Compounds were considered good hits and suitable for testing if they 1) were small (molecular weight ∼200–600 Da), 2) contained a single free amine or carboxyl to facilitate their potential conjugation to other ligands, 3) were not highly charged or highly hydrophobic, 4) did not contain iodine, disulfide bonds or highly reactive functional groups, 5) did not contain multiple conjugated aromatic ring systems, 6) exhibited multiple contacts to the protein surface, and 7) had conformers that bound to the protein surface near one or more E2 amino acid residues that have been shown to participate in or be required for binding to CD81. Detergent-like molecules were avoided and only commercially available compounds were considered for screening.

### Expression and purification of the HCV E2 protein Con1eE2

A construct containing a sequence encoding amino acids 384–656 of the Con1 envelope protein 2 ectodomain (eE2) [19], a genotype 1 E2 sequence, was cloned into a lentiviral expression vector containing a carboxy-terminal Protein A tag separated by a PreScission Protease cleavage consensus sequence. eE2-ProtA was stably expressed in HEK293T cells using lentiviral infection. The protein was secreted into the media and supernatants were purified using IgG Sepharose (GE Healthcare, Piscataway, NJ). eE2-ProtA was eluted with 100 mM sodium citrate and 20 mM KCl at pH 3 directly into tubes containing 1M Tris pH 9 for immediate neutralization. PreScission Protease (GE Healthcare, Piscataway, NJ) was added to the eluted sample at a ratio of 1∶50 (enzyme:eE2), and the digest was then dialyzed into 20 mM HEPES pH 7.5, 250 mM NaCl, 5% glycerol. eE2 was separated from the cleaved tag and the PreScission Protease by ion exchange chromatography [19].

### Experimental analysis of ligand binding to recombinant E2 and CD81-LEL by surface plasmon resonance (SPR) detection

A set of 34 of the ligands predicted by AutoDock to bind to E2 were tested experimentally to determine if they bound to recombinant E2 protein immobilized on a chip using surface plasmon resonance detection. The SPR analyses were performed using a Biacore T100 workstation (GE Healthcare, NJ, USA) and recombinant HCV E2 protein. 1 µM HCV E2 was diluted into 10 mM sodium acetate buffer pH 5 and immobilized for 15 min at a flow speed of 5 µl/min onto a CM5 sensor chip using amine coupling (EDC-NHS). Approximately 10,000 response units (RU) of protein were immobilized on the chip. His-CD81-LEL (Bioclone Inc., San Diego, CA) binding to HCV E2 was tested as a positive control prior to injecting the ligands to confirm the E2 protein was functional and would bind CD81-LEL. In a typical experiment with CD81, 1 µl of his-CD81 (50 nM) in 114 µl PBS was injected into channel 2 and 106.4 RUs of CD81 bound to the E2 on the chip. This was followed by testing the binding of the 34 virtual screening hits where the ligands were prepared as 200 µM solutions in PBS and they were introduced to the protein using a pre-programmed 3 min association and 1 min dissociation interval. The response was measured at two time points during dissociation, 10 and 50 seconds, to obtain information on the rate of ligand dissociation from E2.

Two single cycle kinetic studies were also performed to compare the binding of 281816 to the recombinant E2 and his-tagged CD81-LEL proteins. In both studies, the proteins were diluted to a concentration of 1 µM in 10 mM sodium acetate buffer pH 4.5 and immobilized for 15 min on a CM5 sensor chip using amine coupling (EDC-NHS). Data on the kinetics and affinity of 281816 binding was obtained by flowing five concentrations of the 281816 ligand (2.5 µM, 7.4 µM, 22.2 µM, 66.7 µM and 200 µM) over the chip sequentially at a flow rate of 30 µl/min. Equilibrium binding curves were generated for each protein and the data were fitted using a monovalent binding model to determine the Kd for 281816 binding to E2 and CD81-LEL.

### HCV infection assays

Pseudotyped retroviral particles harboring HCV envelope proteins (HCVpp) from different genotypes were produced as described previously [32], [33] with plasmids kindly provided by F.L. Cosset, J. Ball, and R. Bartenschlager. A plasmid encoding the feline endogenous virus RD114 glycoprotein [34] was used for the production of RD114pp. Both HCVpp and RD114pp expressed *Firefly* luciferase.

The cell culture-produced HCV particles (HCVcc) used in this study were based on the JFH1 strain [35] and were prepared as described previously [36], [37]. They were engineered to express the A4 epitope, titer-enhancing mutations and *Gaussia* luciferase [36], [37].

To identify ligands that inhibit HCV infection, Huh-7 cells were seeded in 96-well plates and treated the day after with six different concentrations of each ligand diluted in DMSO in duplicate using a Zephyr automated liquid handling workstation (Caliper BioSciences, Hopkinton, MA). The final concentration of DMSO (1%) was adjusted to be the same for all ligand concentrations. Cells treated with DMSO were used as negative controls. Cells treated with different concentrations of anti-CD81 antibody (JS-81 from BD Pharmingen, San Jose, CA) 1 hr before infection, were also used as positive controls. The third day, RD114pp, HCVpp or HCVcc were inoculated and incubated for 30 hr at 37°C. *Firefly* and *Gaussia* luciferase assays were performed as indicated by the manufacturer (Promega, San Luis Obispo, CA).

The analysis of the effect of the 281816 ligand on Huh-7 infection by HCVpp bearing envelope proteins from different genotypes was performed in 24-well plates using the method described above. This ligand was also screened for toxicity to the cells using the MTS (3-(4,5-dimethylthiazol-2-yl)-5-(3-carboxymethoxyphenyl)-2-(4-sulfophenyl)-2Htetrazolium) assay [38] and was found to not be toxic under the conditions used in the infection assays.

### Inhibition of recombinant E2 binding to native CD81

Two different assays were performed to test for the inhibition of E2 binding to CD81 by 281816. In a cell binding assay, the human B cell line Raji (ATCC, Manassas, VA), which expresses high levels of CD81 on its surface [39], [40], was used to determine if ligand 281816 inhibits the binding of HCV-E2 protein to native human CD81. Purified HCV-E2 protein (4 µg) was pre-incubated with 1,5,15, 50, 100 or 400 µM of the ligand 281816 for 25 min at RT. After pre-incubation the E2-ligand complex was added to the cells and incubated for 25 min. The complexes were washed from the cells and 0.5 µg of mouse anti E2 antibody (clone H53) was added followed by a FITC-conjugated anti-mouse antibody (Southern Biotechnology, Birmingham, AL). The cells were washed, fixed with 3% paraformaldehyde, and analyzed by flow cytometry (BD FACSCalibur, software: Cell Quest Pro). The mean fluorescence intensity (MFI) was calculated using Flowjo software (TreesStar, www.flowjo.com).

The second test used an ELISA assay to determine if E2 binding to a recombinant CD81 protein is inhibited by the presence of ligand 281816. In this assay, a 96 well plate was coated with GST-tagged human CD81-LEL (5 µg/ml) overnight as previously described [10], then washed with PBS, 0.5% Triton X-100 and blocked with 2% milk in PBS for 1 hr. HCV E2 protein (5 µg/ml) was pre-incubated with different concentrations of 281816 for 30 min before adding to the plate, then HCV-E2 protein (with or without the ligand) was added to the GST-tagged human CD81-LEL coated plate and incubated for 1 hr at room temperature to allow the protein to bind. To detect HCV-E2 binding, a primary mouse anti-E2 antibody (H53 clone, 5 µg/ml) was added and incubated for 1 hr followed by a secondary goat anti-mouse-horse radish peroxidase (HRP) antibody (Southern Biotechnology Associates, Birmingham, AL) diluted 1∶5000. Substrate was added (citrate buffer pH 4.0, 3.5 µl hydrogen peroxide and 100 µl 2,2'-azino-bis(3-ethylbenzothiazoline-6-sulphonic acid)) and the absorbance was measured at 405 nm.

### Inhibition of anti-CD81 5A6 antibody binding to CD81-LEL

To determine if ligand 281816 binds to the E2 binding site on CD81, a competition binding experiment was run using the ligand and an anti-CD81 antibody (5A6 clone) that has been shown previously to block E2 binding [57], [58]. In this assay, a 96 well plate was coated with GST-tagged human CD81-LEL overnight and then blocked with 2% milk in PBS for 1 hr at RT. The plate was then incubated for 40 min with 281816 (1 µM) or PBS as a control. The indicated concentrations of mouse anti-human CD81 antibody (5A6) were added to the plate and incubated for an additional 1 hr, followed by anti-mouse IgG-HRP. The absorbance was then measured at 405 nm.

### HCVcc cell-to-cell transmission assay

Cell-to-cell transmission was measured as described previously [41], [42]. Briefly, Huh-7 cells were seeded on coverslips and infected at low multiplicity of infection with HCVcc for 2 hr at 37°C. After washing, cells were cultured in medium containing neutralizing anti-E2 antibody (3/11; 50 µg/ml) to block cell-free transmission and 281816 at the indicated concentrations. Cells cultured in the presence of DMSO or Epigallocatechin-3-gallate (EGCG, 50 µM) [42] were used as negative and positive controls of inhibition, respectively. Three days post-infection, cells were fixed with formalin solution (formaldehyde 4%, Sigma, St Louis, MO) and stained by indirect immunofluorescence using the anti-E1 monoclonal antibody A4 and Alexa555-conjugated anti-mouse immunoglobulins. Cell-to-cell transmission was quantified by counting the number of infected cells per focus. As a control, cells were infected with HCVcc pre-incubated with 50 µg/ml 3/11 antibody to confirm cell-free transmission is blocked by the antibody as reported previously [41].

### Kinetics of entry

Cells treated with 281816 at 10 µM or with DMSO were infected with HCVcc for 1 hr at 4°C (attachment/binding period). Virus was removed, cells were washed with medium and incubated again for 1 hr at 4°C (post-attachment/binding period). Cells were then washed and incubated for 1 hr at 37°C (endocytosis/fusion period). Lastly, cells were washed and incubated in complete culture medium for 21 hr. Infection levels were monitored by measuring luciferase activities. To confirm 281816 was not toxic to the cells, an MTS assay [38] was also performed after incubating the cells with 10 µM 281816 for the same lengths of time (1 hr, 2 hr or 3 hr) the ligand was exposed to the cells in the entry experiments.

### Antibodies

Mouse anti-E1 A4 [43], anti-E2 H53 [44] and rat anti-E2 3/11 [45] were produced *in vitro* using a MiniPerm apparatus (Heraeus). FITC-conjugated and Alexa555-conjugated anti-mouse immunoglobulins were obtained from Southern Biotechnology (Birmingham, AL) and Jackson Immunoresearch (West Grove, PA), respectively.

## Results

### Structural model of E2

**Figure 1:**
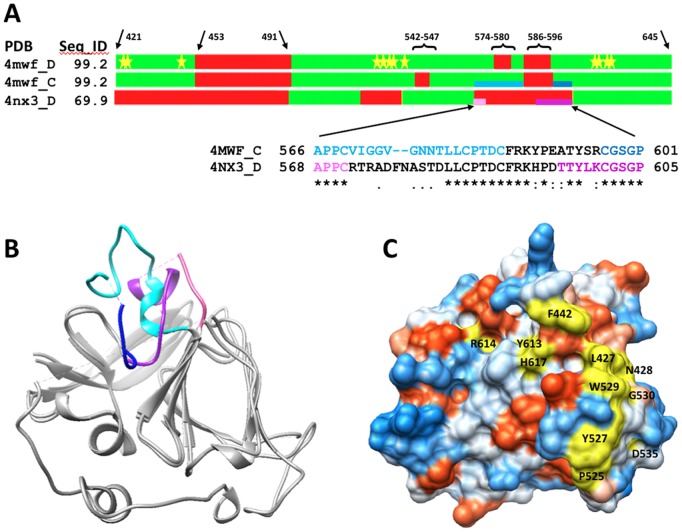
Comparison of structural templates used for modeling the HCV E2c protein. (A) Bar representation of E2 sequence showing the structural similarities between crystal structures 4MWF chains C and D (E2c structure, genotype 1a), and 4NX3 chain D (genotype 2a). Regions reported in the coordinates span amino acid residues from H421 to N645. The percent sequence identities between amino acid sequences taken from coordinates and corresponding sequence fragments from HCV E2 protein of genotype 1a are shown in the column Seq_ID. In green are colored regions where structural deviations are below 3 Ångstroms measured as Cα-Cα distances between corresponding residues from the superimposed structures. In red are regions where structural data is missing or deviations are greater than 3 Angstroms. The locations of amino acid residues that have been reported to be important for E2 binding to CD81 are marked with yellow stars. (B) Structural superposition of 4mwf_C and 4nx3_D shows strong conformational similarities between experimentally solved structures of E2 proteins for which the level of sequence identity is 69%. In blue and purple are colored structural fragments where two structures 4mwf_C (566–601; Blue: light-dark) and 4nx3_D (568–605; Purple: light-dark) significantly differ. (C) Surface presentation of the 4mwf_D structure showing the amino acid residues identified to be important for E2 binding to CD81 (yellow). The other amino acid residues are color coded with the most hydrophilic residues being colored blue, the most hydrophobic residues colored red orange, and intermediate residues colored white.

In order to maximize the likelihood that these experiments would lead to the discovery of small molecules that bind to E2 and block E2’s binding to CD81, we created a homology model of the core of the E2 protein to use as our docking target. This model was created using the HCV genotype 1a protein sequence NP_751921.1, which corresponds to isolate H77, and the crystal structure of E2c as the primary template (PDB entry: 4MWF). Using a model, rather than the E2c crystal structure, was important because the reported crystal structure of E2c has three large gaps in which atom coordinates for 57 amino acids, or one quarter of the E2c structure, is missing. The coordinates listed in PDB chains 4mwf_C and 4mwf_D provide structural information for only 169 and 171 residues respectively out of the 363 amino acids present in the full-length E2 protein. Within each of the deposited PDB chains, three stretches of amino acid sequence (large loop P453-P491 containing 39 amino acids, T542-G547 or V574-N577, and F586-R596) are missing from the structure (Figure 1A). Docking to structures lacking such a large proportion of their amino acids can be problematic because the missing peptide segments are usually located on the protein’s surface, and the underlying amino acid residues packed in the interior of the protein are exposed and incorrectly presented as the surface during the docking. Unfortunately, similar regions are also not present in the crystal structure of the genotype 2a HCV E2c protein (PDB chain: 4nx3_D) reported by Kahn et al. [19] which provides atom coordinates for only 119 amino acids. Structural superposition of 4mwf_C and 4nx3_D (Figure 1B) shows strong conformational similarities between the experimentally solved structures of the E2 proteins with a root mean square deviation of 1.07 Angstroms measured on 98 residues for which distances between corresponding Cα atoms are under 3 Angstroms. The most significant structural deviations are observed in the region 566–601 (numbering from 4mwf_C) which corresponds to the region that also exhibits the greatest variation in sequence (see sequence alignment in Figure 1A).

**Figure 2:**
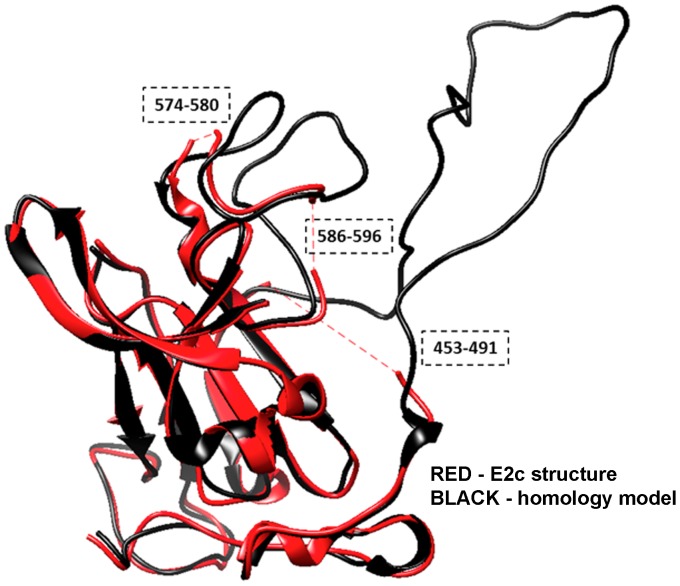
Comparison of the crystal structure of E2c with the homology model. Structural superposition between E2c crystal structure from the PDB chain 4mwf_D (red) and the homology model (black) is illustrated using a ribbon representation. The crystal structure and homology model overlap in most of the regions, except the fragments where coordinates in the experimental structure are missing (red dashed lines).

Exhaustive structure similarity searches of 90 residue structural fragments of E2 conducted using the entire PDB database (255,302 PDB chains) revealed that no additional structural homologs could be found at the level of calculated structure similarities by LGA score [46] higher than LGA_S = 45%, suggesting that the HCV E2 protein represents a novel fold in the current PDB. Thus, the modeling of the structure of the insertions needed to fill in missing regions in the experimentally solved structures and to complete the model was a difficult task, and it was completed with a very low degree of confidence. By applying a combination of structural modeling and analysis methods to the E2 crystal structure (see Materials and Methods section), we were able to construct a model (Figure 2) that contains the 57 amino acids that are missing in the E2c structure, including an amino acid known to be critical for E1 binding (W487), key amino acids known to participate in CD81 binding (Figure 1C), as well as the exact sequence for the HCV genotype 1a E2 protein. Three regions of the protein that have been identified by others to be critical for E2 binding to CD81 [47]–[49] are contained in the model in their entirety. Currently, however, only three of the twenty-one Region 1 amino acids (H421–N423) are present in the model. A comparison of our model to the two E2c structures (see bar plots in Figure 1A and superposition of the E2c structure and the model in Figure 2) shows the main core regions are, as one would expect, very similar. The differences that are observed in the core region are small and appear to reflect only minor local deviations between experimentally solved structural templates. The large region that does differ corresponds to the missing peptide segments.

### Ligands predicted to bind to CD81 binding sites on E2

**Figure 3:**
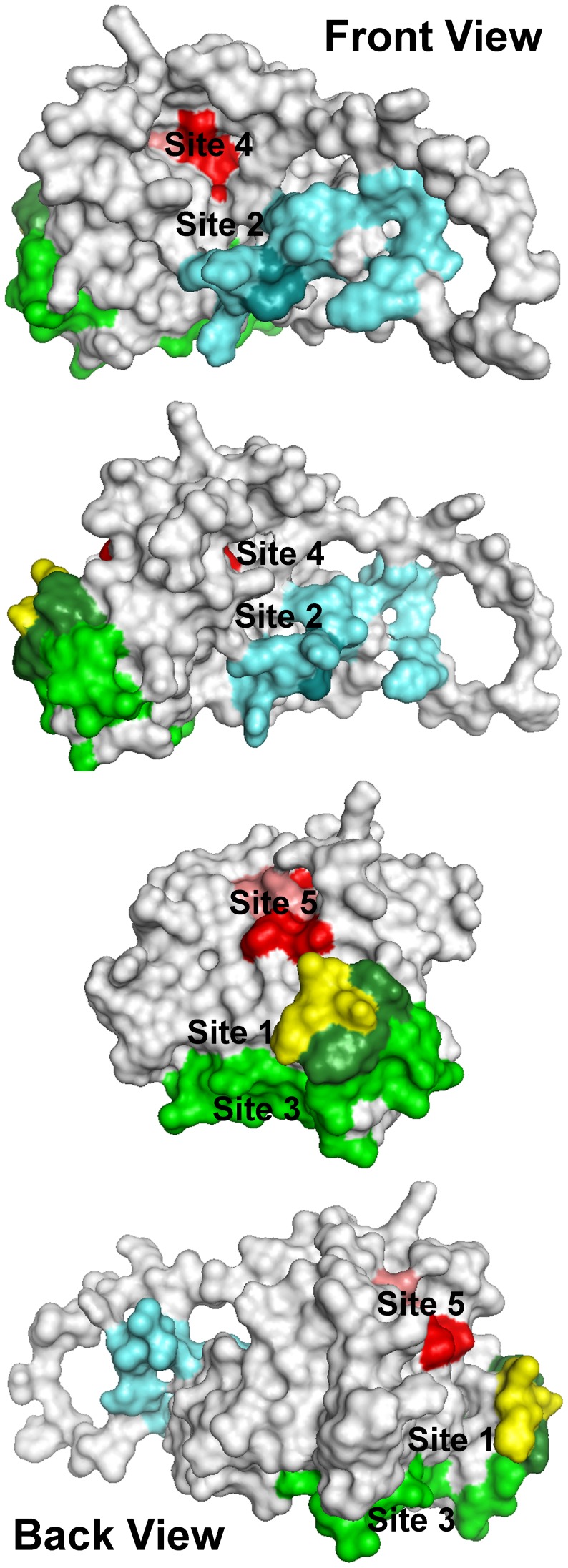
Location of ligand-binding sites on the E2 homology model used to select ligands for testing. Each of these sites either covers or is located immediately adjacent to amino acids or peptide segments of the E2 protein known to be important for HCV infectivity. H421–N423 (yellow): each amino acid in this region is important for infectivity. Antibodies binding to amino acids Y474–R492 (light cyan) have been shown to prevent infectivity, but this region of the protein has no effect on E2 binding CD81. W487 (dark cyan) is a key amino acid that is involved in E2 binding to E1. S522–G551 (light green) and Y527 and W529 (dark green) are critical for E2 binding to CD81. Site 4: P612, Y613, and H617–P619 (red) are critical for E2 binding to CD81; mutations to R614–W616 (pink) disrupt the structure of the region. The four views show the structure as it is rotated counterclockwise from left to right. Movie S1 shows the rotating structure.

Five ligand-binding sites on the HCV E2 homology model (Figure 3) were identified by docking the National Cancer Institute’s Diversity Set III library of ligands to the E2 model. Each of these sites is associated with or positioned next to one or more of the amino acid or peptide sequences that have been identified by others to either participate in E2 binding to CD81, E2 binding to E1, or to be important for HCV infectivity. While the accuracy of the structure of the modeled segments missing from E2c may be low, the docking and visual inspection processes focused on the regions of the target that were based on the crystal structure. The majority of the amino acids that make up or surround each of the cavities used for ligand docking and the neighboring amino acids that play a role in E2 binding to CD81 are all located in the core region of E2. The structure of this region of the model is known with high confidence, as it is essentially identical to the two recent crystal structures of the E2 protein core determined by two different groups [18], [19]. The locations of the grid boxes were also defined in such a manner that the amino acids in the modeled segments missing from the crystal structure of E2c would only be marginally considered during the docking. Only those residues in close contact with the core E2c structure were included in the boxes. In this way, the regions of the homology model with the least well-defined structures had a minimal impact on the docking results.

The first sequence of importance is the peptide segment Q412–N423 that was identified to bind to the broadly neutralizing antibody AP33 [20], [50]. Alanine mutagenesis studies have shown all of the amino acids in this region appear to be important for HCV infectivity [48]. The model used in this study currently contains only three of the amino acids that correspond to this segment, H421, I422 and N423. Sequence 2 spans the second hyper-variable domain of E2, extending from amino acid Y474 to R492 [13], [47]–[49]. The majority of amino acids in this sequence have been shown to have no effect on E2 binding to CD81 when mutated [51], but antibodies binding to this region of the protein do inhibit HCV infectivity [49] and CD81 binding [50]. One amino acid located within sequence 2, W487, does however appear to be critical for E2 binding to E1. This amino acid is the first residue in one of the WHY motifs that have been reported to play a role in E1:E2 dimerization [47]. The third sequence spans amino acids S522–G551 [20], [47]–[49] and the fourth sequence of importance is comprised of amino acids P612–P619 [49]. Mutations of residues Y527, W529, D535, Y613, R614, W616, H617 and Y618 in these two regions have all been shown to eliminate E2 binding to CD81 [47], [49]. Mutating all but three of these amino acids (D535, R614 and W616) appears to eliminate specific interactions with CD81. W616 is the first amino acid in another WHY motif that is located in a region (G600–C620) that has been shown to be involved in fusion [52]. Alanine mutagenesis of D535, R614 and W616 was found to disrupt the structure of the AR3A epitope and indirectly impact CD81 binding [49].

These five binding sites were used to guide to our selection of the top virtual screening hits to be tested experimentally for binding to recombinant E2 protein. All five sites are cavities in the protein surface that would be expected to be accessible to ligands because they contain or are surrounded by amino acid residues known to participate in E2 binding to CD81 or they are located within the epitopes of antibodies that inhibit HCV infectivity or block CD81 binding. While there is still some debate regarding the importance of the entire regions bound by neutralizing antibodies, amino acid mutagenesis studies have provided a great deal of insight into those amino acids located within the epitopes that participate in E2 binding to CD81. Based on this information, we have used the set of amino acids W420–I422, S424, G523, Y527, W529, G530, D535, P612–R614 and W616–P619, whose mutation has been shown to eliminate E2 binding to CD81, to identify locations within these sites (Figure 3) where ligand binding would be expected to disrupt E2’s ability to bind to CD81.

Thirty-four of the highest scoring ligands were selected from the docking run for experimental analysis. Docked conformations of each of the ligands were predicted to bind to one or more of these five binding sites. The best ligands were considered to be those that exhibited the lowest free energy of binding and were predicted to interact with or bind nearby one or more of the E2 amino acids within the sites that were reported to be critical for E2 binding to CD81. The free energy of binding predicted for the best bound ligand conformations, shown in Table 2, ranged from −6.2 to −8.7 kcal/mol. Additional criteria used to select among the group of ligands predicted to bind include the number of contact points/interactions (such as hydrogen bonds, salt bridges, van der Waals interactions) with amino acids in the model (the larger number of contacts or interactions the better) and the chemical structure of the ligands (preference is given to those that contain a free amino or carboxyl group that is exposed to solvent). Ligands with free amino or carboxyl groups can easily be linked to other ligands to create higher affinity or more selective second-generation inhibitors. Compounds that have been reported previously to be highly toxic were excluded.

**Table 2:** Ligands predicted to bind to the HCV E2 protein by blind docking of the NCI Diversity Set III small molecule library to the HCV E2 structural model and their predicted free energies of binding.

Ligand ID (NSC#)	Free Energy of Binding (kcal/mol)	Ligand ID	Free Energy of Binding (kcal/mol)
670283	−7.69	211490	−8.7
86467	−7.47	113486	−6.26
639174	−7.81	144694	−7.27
81462	−6.81	4429	−7.3
403379	−7.58	133071	−7.5
213700	−7.89	163910	−7.4
359472	−7.91	54709	−7.3
146554	−7.67	135618	−8.7
204232	−8.54	281254	−6.5
281816	−8.64	319990	−7.4
308835	−8.4	369070	−6.3
60785	−7.48	59620	−7.3
84100	−6.99	38968	−3.9
158413	−7.9	171303	−5.8
57103	−6.36	228155	−8.7
121861	−8.16	13316	−6.8
3076	−7.71	117268	−7.6

### Experimental confirmation of ligand binding to HCV E2

**Figure 4:**
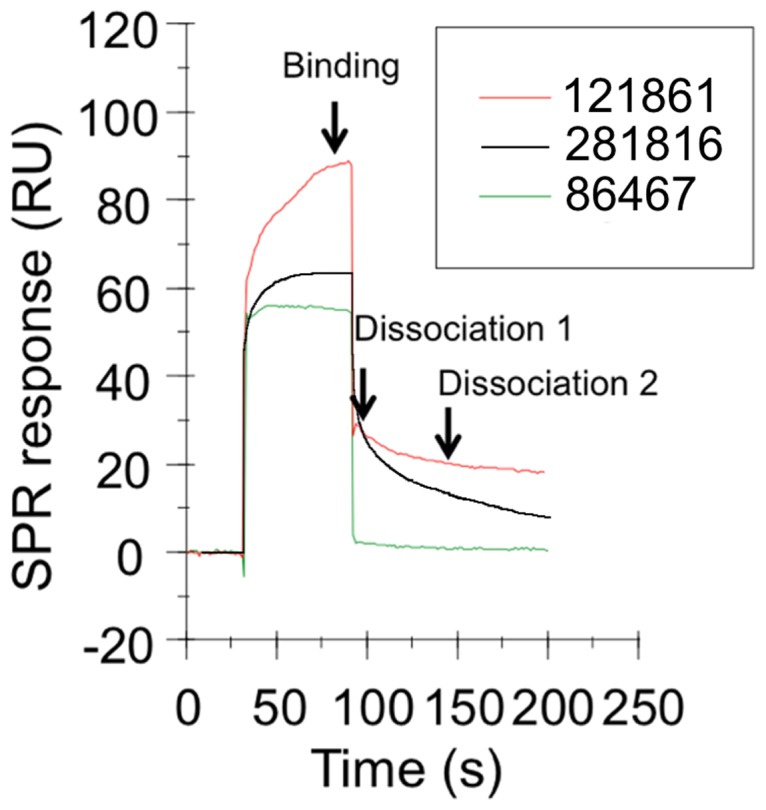
Surface plasmon resonance sensorgrams of ligands binding to recombinant E2 protein (Biacore T100). This figure shows sensorgrams (binding and dissociation plots) for three of the ligands that bound to the recombinant E2 protein immobilized on a CM5 chip, 281816 (black), 86467 (green) and 121861 (red), and the three reference points that are used to measure the binding and dissociation (dissociation 1 and dissociation 2) of the compound expressed in response units (RU).

Each of the 34 ligands was tested experimentally using surface plasmon resonance (SPR) detection (on a Biacore T100 instrument) to determine if it would bind to recombinant HCV E2 protein and to obtain an assessment (relative to the other ligands) of how well it binds. Twenty-three of the molecules provided a positive change in response units (RUs) indicating they bound to the E2 protein immobilized on the chip (Table 3). The measured responses for the ligands that bound varied from 54 to 276 RUs. Data was also obtained on the rate of ligand dissociation by measuring the amount of ligand remaining bound at two time points, dissociation 1 (10 seconds) and dissociation 2 (50 seconds), during the rinsing of the chip with buffer (Figure 4). The majority of the ligands dissociated quickly, as one might expect for small molecules that bind to the surface of a protein. A few, such as ligands 121861, 4429, 158413, 81462, and 57103, exhibited slower off rates when compared to others.

**Table 3:** Magnitude of surface plasmon resonance binding response obtained for the 23 ligands that were identified to bind to recombinant E2 protein immobilized on a CM5 sensor chip.

Ligand ID (NSC#)	Binding (RU)	Dissociation 1 (RU)	Dissociation 2 (RU)
670283	54.3	4	1.4
86467	54.9	1.9	0.8
639174	55.4	2.3	0.6
81462	57.2	9.2	6.5
403379	58	2.8	1.1
213700	62	3.1	0.8
359472	62	2.5	0.8
146554	63.4	3.1	0.8
204232	63.4	2.5	0.4
281816	64.5	3.7	0.9
308835	64.8	7.1	5.2
60785	70.4	2.8	0.6
84100	71.2	4.2	2.2
158413	71.2	10.3	8.5
57103	81.6	11.4	2.5
121861	88.4	26.1	20.4
117268	88.5	4.1	1.2
3076	92.2	3.2	1.6
211490	102.9	6.1	2.1
113486	104.7	7	2.6
144694	118.8	6	2.3
4429	155.3	28.9	14.2
133071	276.3	1.8	−2

The rate of ligand dissociation is assessed by measuring the response units at two time points (10 sec and 50 sec) after the chip with bound ligand is rinsed with buffer.

### Inhibition of HCV entry

**Figure 5:**
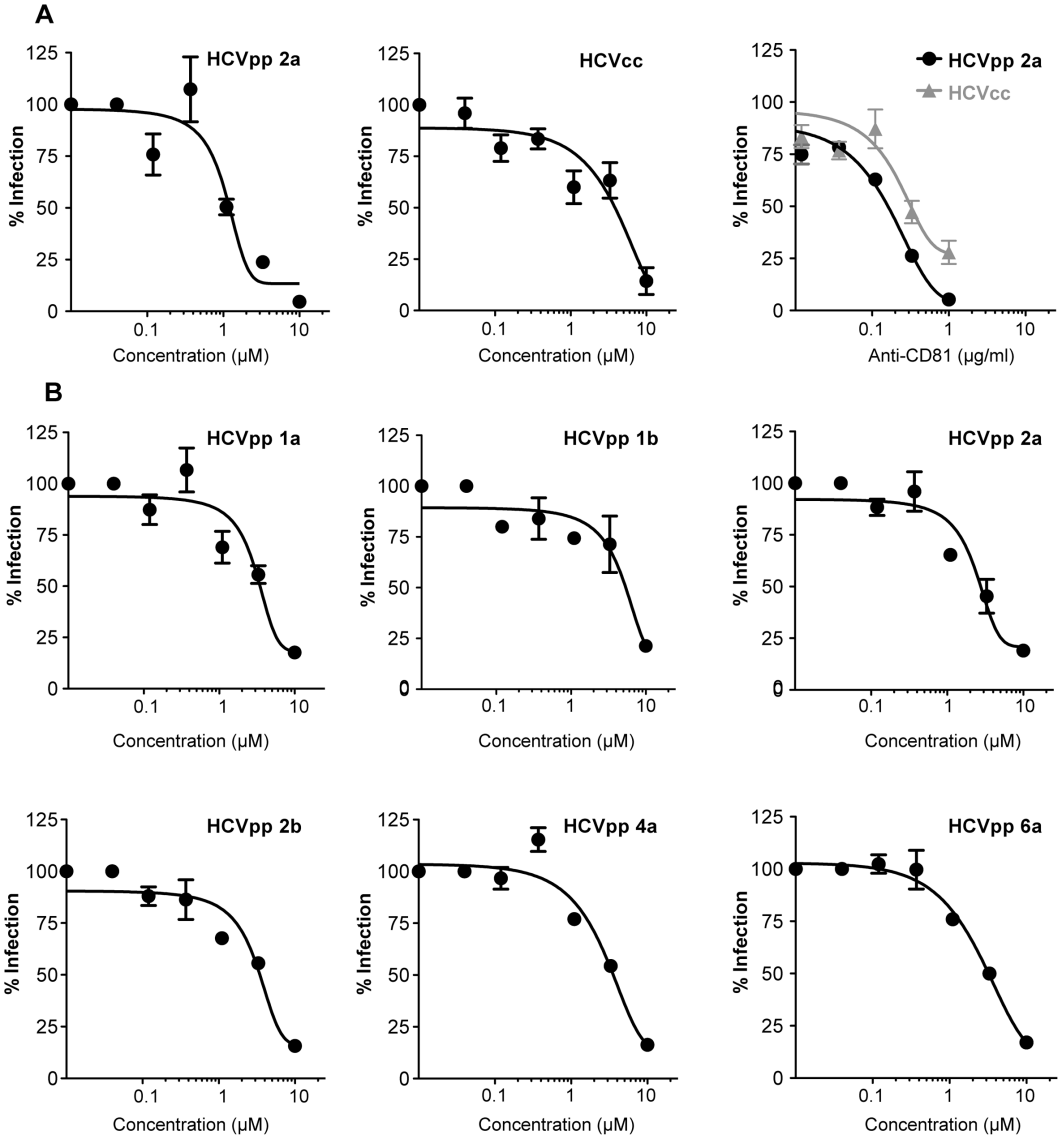
281816 inhibits HCV entry in a genotype-independent manner. (A) Huh-7 cells in 96-well plates were pre-treated with 281816 (left and middle panels) or anti-CD81 antibody (right panel) at the indicated concentrations and then infected with HCVpp 2a or HCVcc. (B) Huh-7 cells in 24-well plates were pre-treated with 281816 at the indicated concentrations and infected with HCVpp expressing envelope proteins from the indicated genotype. After 30 hr of infection, cells were lysed and luciferase activities quantified. HCVpp infections were normalized to RD114pp infections.

The 23 compounds that were observed to bind to recombinant E2 protein were then tested to determine if they would block HCV infection of Huh-7 cells. Pseudotyped retroviral particles harboring the envelope protein of an endogenous feline retrovirus (RD114pp) were first used to determine the specificity and the safety of molecules. We excluded from a further characterization the molecules for which the half maximal inhibitory concentration (IC50) against RD114pp was lower than 10 µM or the molecules that significantly increased RD114pp infection (Table 4). The remaining ligands were next tested against pseudotyped retroviral particles harboring genotype-2a HCV envelope proteins (HCVpp 2a), cell culture produced HCV particles (HCVcc) or RD114pp. As a positive control, an anti-CD81 antibody (JS-81) was included in the assays. One compound, 281816, showed an inhibitory effect on both HCVpp and HCVcc infection with IC50’s of 1.02 µM and 3.95 µM, respectively (Table 4 and Figure 5A), indicating that this molecule inhibits the entry step of the HCV lifecycle, probably through a specific effect on the virus’s interaction with CD81. Huh-7 cell toxicity was not observed over the range of ligand 281816 concentrations tested in these assays.

**Table 4:** The IC50 values obtained for the 23 ligands screened for their ability to inhibit HCVcc, HCVpp and RD114pp infection of Huh-7 cells.

Ligand ID(NSC#)	IC50 (µM)		
	RD114pp	HCVpp	HCVcc
670283	3	ND	ND
86467	>10	>10	>10
639174	0.03	ND	ND
81462	>10	>10	>10
403379	>10	>10	>10
213700	>10	>10	>10
359472	>10	>10	>10
146554	>10	ND	ND
204232	>10	>10	>10
**281816**	**>10**	**1.02**	**3.95**
308835	>10	>10	>10
60785	3.5	ND	ND
84100	>10	>10	>10
158413	>10	>10	>10
57103	0.3	ND	ND
121861	>10	>10	>10
117268	0.1	>10	>10
3076	0.25	ND	ND
211490	0.5	ND	ND
113486	>10	>10	>10
144694	>10	>10	>10
4429	>10	>10	>10
133071	0.10	ND	ND
Anti-CD81	>10*	0.17*	0.36*

ND refers to molecules that were not assayed because the molecule was not specific for HCV (it inhibited RD114pp infection). *IC50 values for anti-CD81 antibody are in µg/ml.

To determine if 281816 would inhibit HCV genotypes other than 2a, a series of infection assays was performed with HCVpp bearing envelope proteins from a number of different HCV genotypes. Interestingly, 281816 was found to be equally effective in inhibiting Huh-7 infection by all the HCV genotypes tested (1a, 1b, 2a, 2b, 4a and 6a, Figure 5B). The IC50 values ranged from 2.2 µM to 4.6 µM (Table 5).

**Table 5:** Genotype independent inhibition of HCVpp infection of Huh-7 cells by ligand 281816.

Subtypes	IC50 (µM)
HCVpp 1a	2.95
HCVpp 1b	4.66
HCVpp 2a	2.22
HCVpp 2b	2.93
HCVpp 4a	3.44
HCVpp 6a	3.30

**Figure 6:**
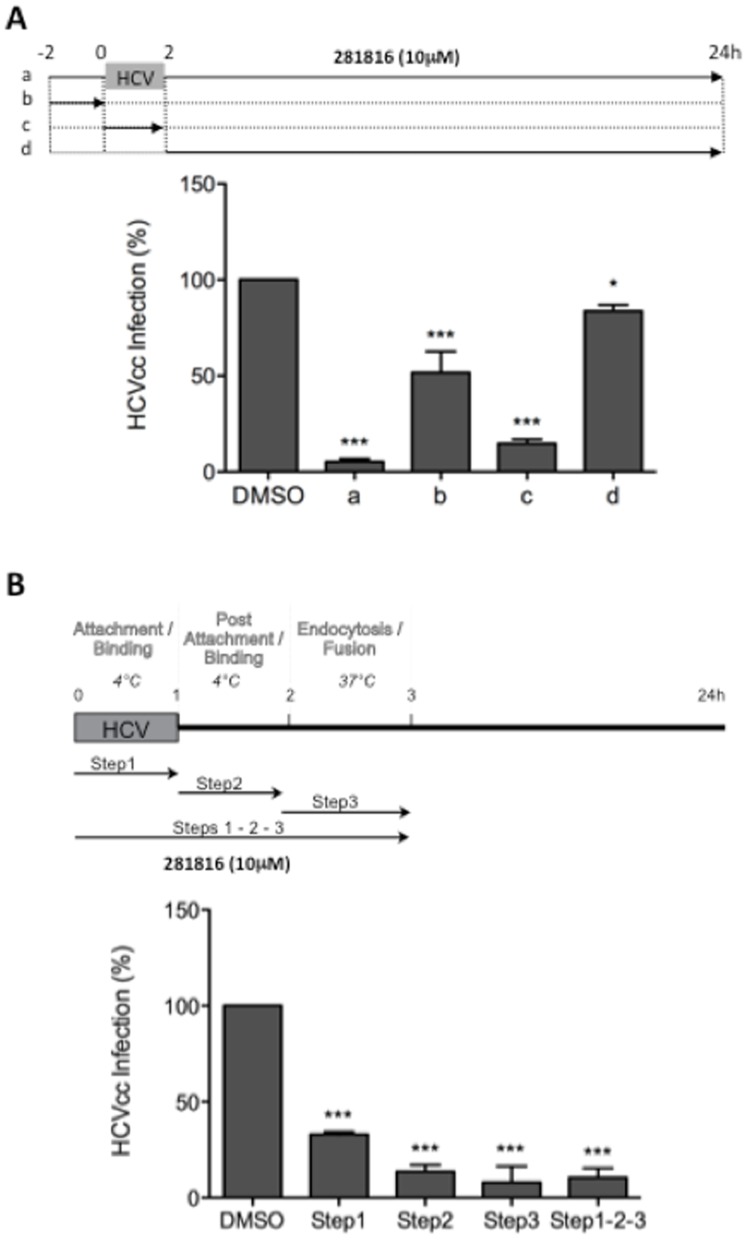
281816 inhibits HCV entry. (A) Huh-7 cells in 24-well plates were treated at different time points with 281816 at 10 µM and infected with HCVcc for 2 hr at 37°C. 281816 was added full-time during the experiment (a), 2 hr before virus inoculation (b), 2 hr during virus inoculation (c), or full-time after virus inoculation (d). (B) Huh-7 cells were infected with HCVcc for 1 hr at 4°C (Step 1: attachment/binding), then virus was removed and cells incubated again at 4°C for 1 hr (Step 2: post-attachment/binding). Finally, cells were shifted at 37°C for 1 hr (Step 3: endocytosis/fusion) and left at 37°C for 21 hr. 281816 was added at 10 µM either during the Step 1, Step 2, Step 3 or Steps 1-2-3. * and *** indicate p values below 0.05 and 0.0001, respectively.

To confirm that 281816 inhibits HCV entry with no further effect on post-entry steps, 281816 (10 µM) was added at different time points (Figure 6A) before (−2 to 0 hr, b), during (0 to 2 hr, c), or after (2 to 24 hr, d) inoculation of Huh-7 cells with HCVcc, as previously described [53]. Cells treated with dimethylsulfoxide (DMSO) and cells treated continuously (−2 to 24 hr, a) with 281816 were used as controls. The results clearly showed that 281816 significantly inhibits HCVcc infection when present during virus infection (Figure 6A, c). The decrease in HCVcc infection that was observed in condition b is likely to be due either to some 281816 remaining bound to the cell after the washing step or its entering into the cells and acting on the entry step (Figure 6A, b). Similarly, a slight decrease was also observed in condition d (Figure 6A), which is likely related to 281816 acting on the entry of the remaining particles (those entering after 2 hr). Together, these results confirm that 281816 inhibits the entry step of HCV lifecycle.

**Figure 7:**
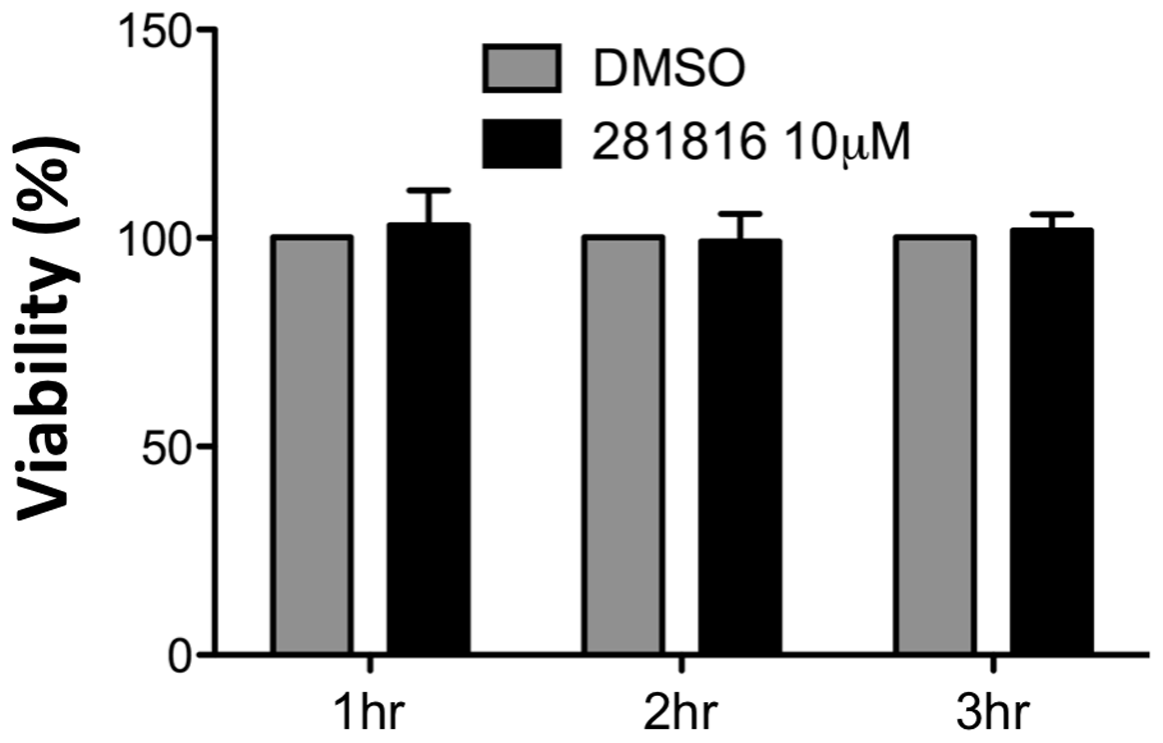
Viability of Huh-7 cells treated with 281816 in the HCV entry experiments. An MTS assay [38] was used to determine the viability of cells treated with 10 µM 281816 in DMSO (and DMSO alone, as a control) for 1 hr, 2 hr, or 3 hr under the same conditions used in the HCV entry experiments. 281816 is not toxic under any of the conditions used in this assay. There were no significant differences between the 281816 treated and control samples (p values <0.05).

After attachment to the cell surface and binding to entry factors, HCV virions are internalized by clathrin-mediated endocytosis [54], [55]. Following internalization, HCV is transported to early endosomes along actin stress fibers, where fusion seems to take place [55], [56]. To determine which step in HCV entry is impaired by 281816, we administered the ligand at different intervals during the early phase of infection. Virus attachment and binding were performed at 4°C (Figure 6B, Steps 1 and 2), Then, cells were shifted to 37°C to allow endocytosis and fusion (Figure 6B, Step 3). Cells treated with JS-81 were used as controls. The addition of 281816 during step 2 and step 3 led to the strongest inhibition of HCV infection, as strong as the one observed when 281816 was present during all three steps. We also observed a significant inhibition of HCV infection when 281816 was added during the early attachment/binding steps (Figure 6B, Step 1). An MTS assay performed with 10 µM 281816 for each length of time the cells were treated with 281816 (1 hr, 2 hr, and 3 hr) showed the compound was not cytotoxic to the cells under the conditions used in the assay (Figure 7). Together, these results indicate that 281816 inhibits HCV infection by acting on more than the first (attachment/binding) step of viral entry. These data suggest the ligand also affects interactions during HCV internalization and fusion.

### Blocking of E2 binding to CD81

**Figure 8:**
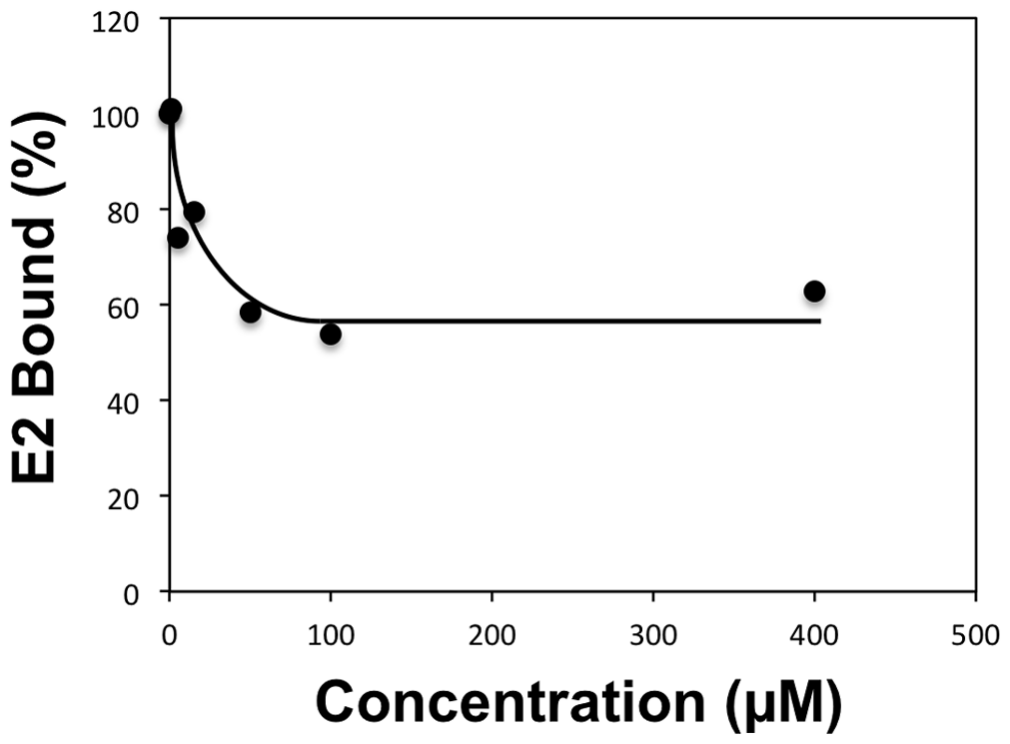
281816 inhibition of HCV E2 protein binding to native CD81 on Raji cells. Flow cytometry was used to quantify recombinant HCV E2 protein binding to native CD81 over-expressed on Raji cells. Binding of the recombinant E2 protein to native CD81 on the surface of Raji cells was detected using the mouse monoclonal E2 antibody clone H53 followed by staining with a secondary FITC anti-mouse antibody. E2 binding is inhibited by 281816 in a dose-dependent manner up to 100 µM.

**Figure 9:**
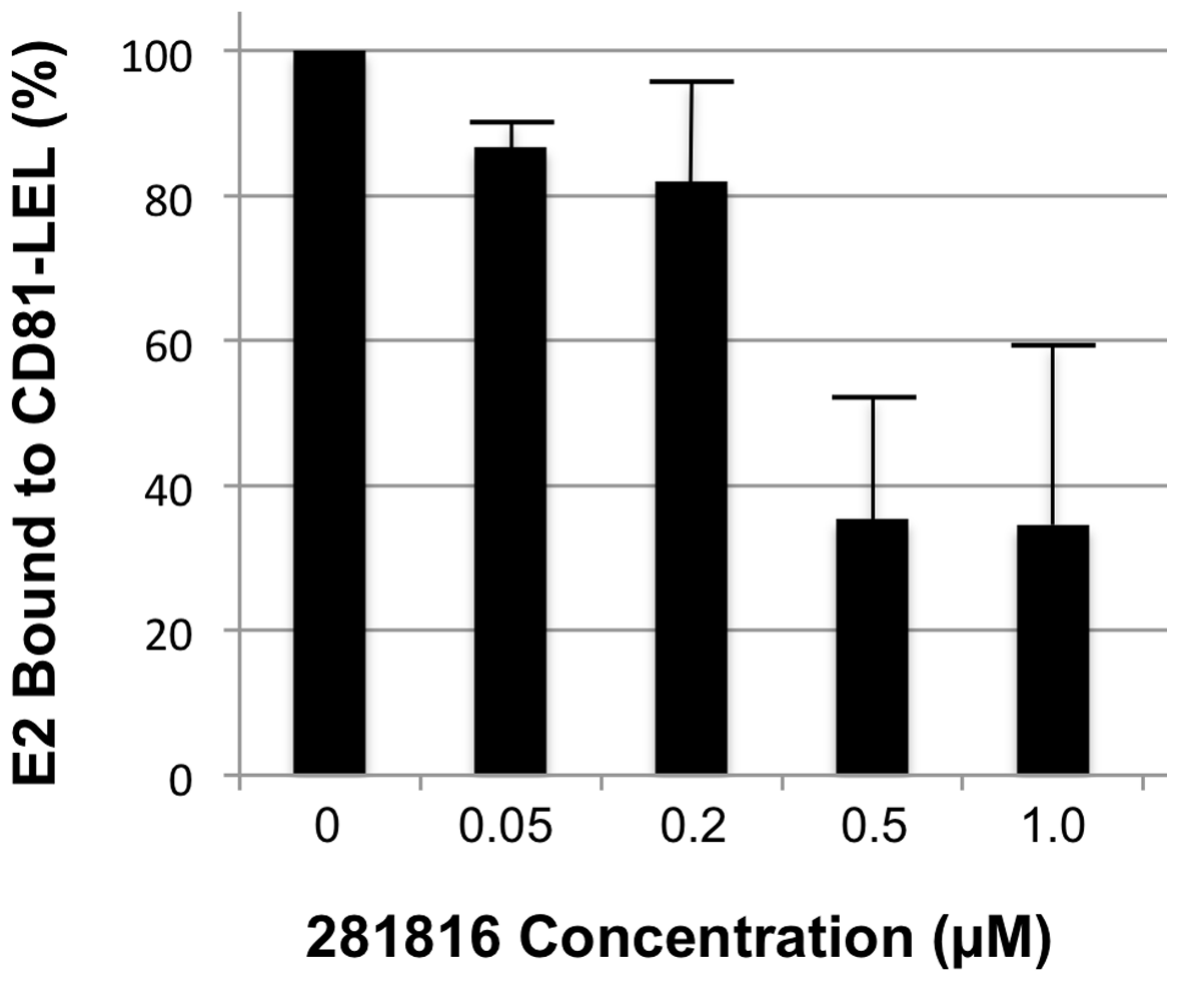
Ligand 281816 inhibits HCV-E2 binding to recombinant CD81-LEL. Binding of recombinant E2 protein to GST-tagged human CD81-LEL immobilized on a 96 well plate was determined using an ELISA assay. The plate was coated with GST-tagged human CD81-LEL (5 µg/ml) overnight as previously described [10], HCV E2 protein (5 µg/ml) was pre-incubated with different concentrations of 281816 for 30 min before adding to the plate, and the HCV-E2 protein (with or without the ligand) was then added to the GST-tagged human CD81-LEL coated plate and incubated for 1 hr. HCV-E2 binding was detected using a primary mouse anti-E2 antibody (H53 clone) and a secondary goat anti-mouse-HRP antibody by measuring the absorbance at 405 nm. The results, which are plotted as percent of E2 protein bound to CD81-LEL relative to E2 binding observed in the absence of the ligand (buffer control), show a dose-dependent effect of 281816 on the inhibition of the E2 protein binding to immobilized recombinant CD81-LEL. P values for the 0.05, 0.2, 0.5 and 1.0 µM 281816 samples are 0.0069, 0.0195, 0.0006 and 0.0009 respectively.

Ligand 281816 was originally selected for testing based on the prediction by docking that it would bind to a site on the HCV E2 protein where CD81 binds. The infection assay conducted with Huh-7 cells demonstrated 281816 is effective in inhibiting the entry step in the HCV life cycle. To confirm that the binding of 281816 to E2 inhibits the HCV E2-CD81 interaction, flow cytometry was used to monitor the binding of a recombinant form of the E2 protein to native CD81 overexpressed on Raji cells as a function of 281816 concentration. The results in Figure 8 show binding of the E2 protein to Raji cells is inhibited by 281816 in a dose dependent manner. Using a second technique (an ELISA-based assay), we observed a similar dose-dependent effect of 281816 on the inhibition of the E2 protein binding to recombinant CD81-LEL immobilized on micro titer plates (Figure 9). While an IC50 for 281816 blocking the binding of E2 to CD81 could not be determined from the flow cytometry data, the ELISA results indicate the IC50 is in the range of 0.2–0.5 µM.

A blind docking experiment with 281816 has also suggested the ligand may bind to several sites on CD81, including one that is located within the region bound by E2. 281816 binding to CD81 or other cellular proteins could explain the 281816 retention observed in washed cells in the HCV entry experiments. Such binding would likely be of little consequence, unless the ligand were to be bound within the E2 binding site on CD81 and were to block the E2:CD81 interaction by targeting both proteins.

**Figure 10:**
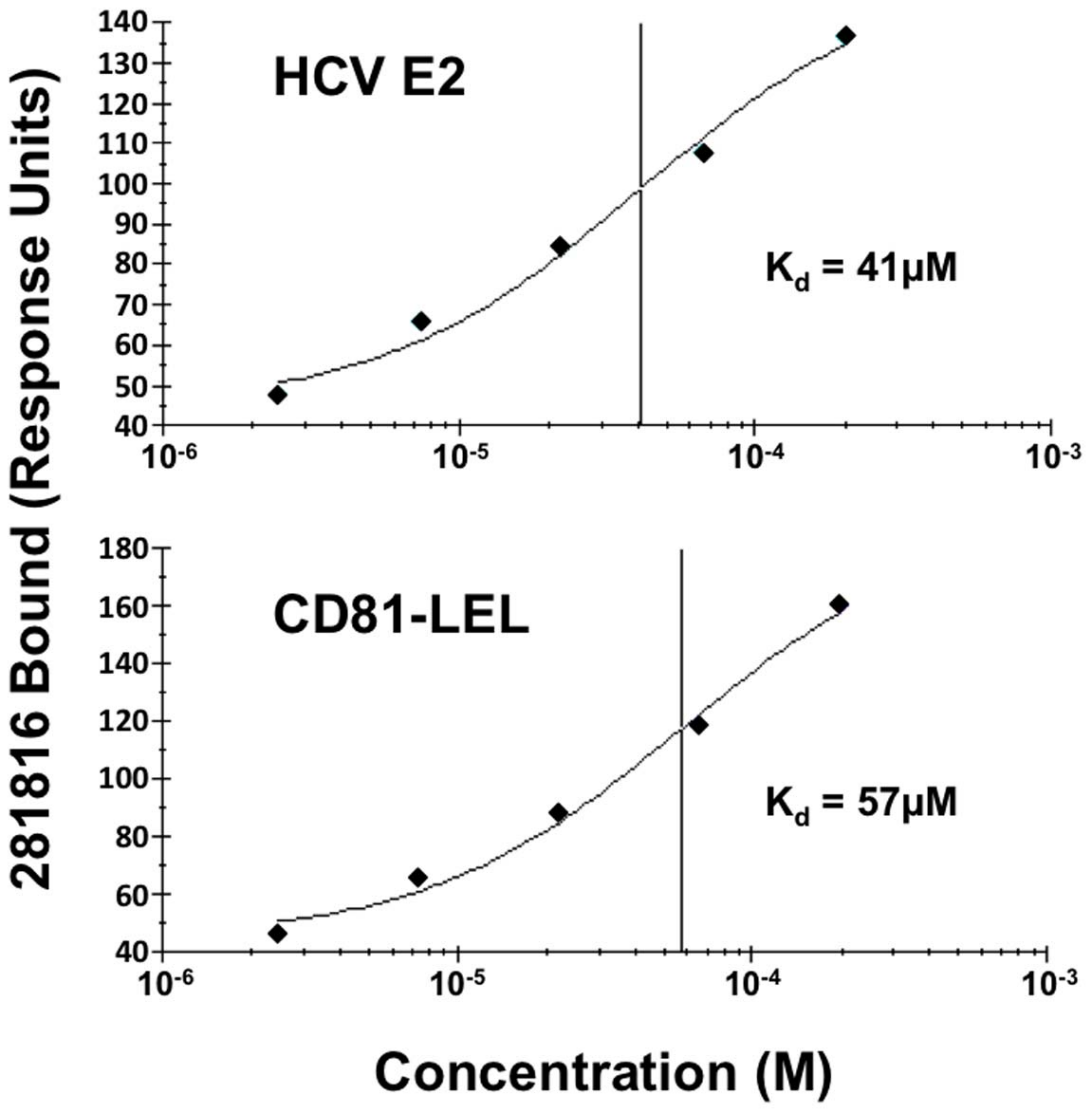
Single cycle kinetics of 281816 binding to recombinant HCV E2 and his-tagged CD81-LEL. Using surface plasmon resonance detection, ligand 281816 is observed to bind to both HCV E2 and CD81-LEL proteins immobilized on a CM5 chip. Analyses of the binding kinetics were used to obtain dissociation constants for 281816 binding to recombinant E2 (K_d_ = 41 µM) and recombinant his-tagged CD81-LEL (K_d_ = 57 µM) protein.

**Figure 11:**
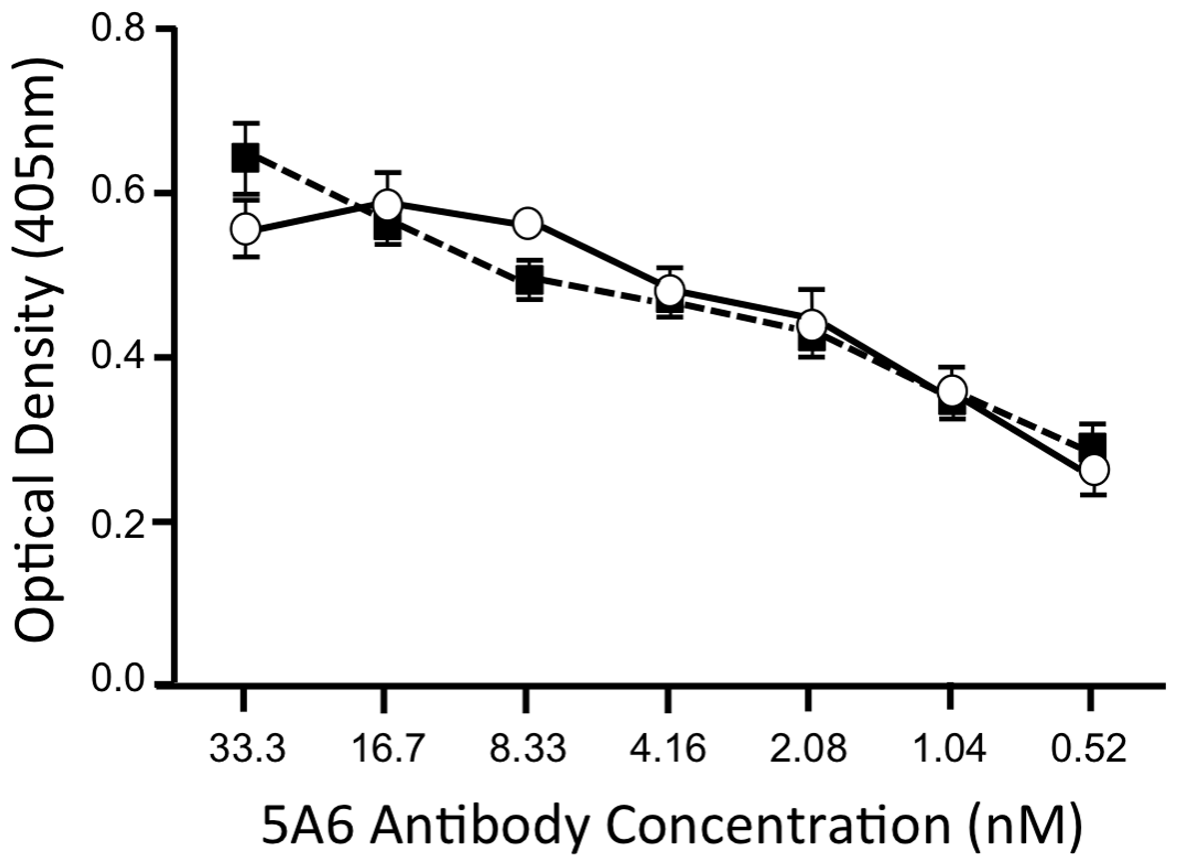
Ligand 281816 does not compete with binding of an anti-CD81 antibody to human CD81-LEL. Binding of anti-CD81 monoclonal antibody 5A6 to recombinant GST-tagged human CD81-LEL protein was determined by ELISA. Serial dilutions of 5A6 antibody were incubated with the CD81-LEL protein in the presence of 1 µM ligand 281816 (black squares) or PBS as a control (open circles). The amount of 5A6 antibody bound to CD81 remained the same in the presence and absence of the ligand, demonstrating that 281816 does not bind sufficiently well to the E2 binding site on CD81 to block 5A6 binding, even when the antibody concentration was reduced to 1/2000^th^ the concentration of the ligand.

To determine if ligand 281816 also binds to CD81, surface plasmon resonance was used to test for 281816 binding to recombinant CD81-LEL protein immobilized on a chip. As shown in Figure 10, 281816 does bind to CD81-LEL. A kinetic analysis of this binding has shown that the ligand binds to CD81-LEL (Kd = 57 µM) almost as well as it binds to E2 (Kd = 41 µM). However, a competition ELISA experiment that used the anti-CD81 antibody, 5A6, that blocks E2 binding to CD81 (Figure 11) revealed that 281816 does not bind to the E2 binding site on CD81. In this experiment, CD81-LEL was immobilized on a micro titer plate and the binding of the 5A6 antibody was monitored in the presence of 1 µM 281816 as a function of antibody dilution. The antibody 5A6 has been shown previously to bind to the same site on CD81 recognized by E2 [57], [58] with an affinity (75 nM) [59] about 1/10th that of E2 (4–10 nM) [60], [61]. Working at the same concentration of 281816 (1 µM) that provided the best inhibition of E2 binding to CD81-LEL, 281816 did not inhibit the 5A6 antibody binding to CD81 even when the antibody concentration was reduced to 1/2000^th^ the concentration of the ligand (Figure 11). The amount of 5A6 antibody bound to CD81 remained the same in the presence and absence of the ligand, demonstrating that 281816 does not bind sufficiently well to the E2 binding site on CD81 to block 5A6 binding.

### 281816 abrogates HCV cell-to-cell transmission

**Figure 12:**
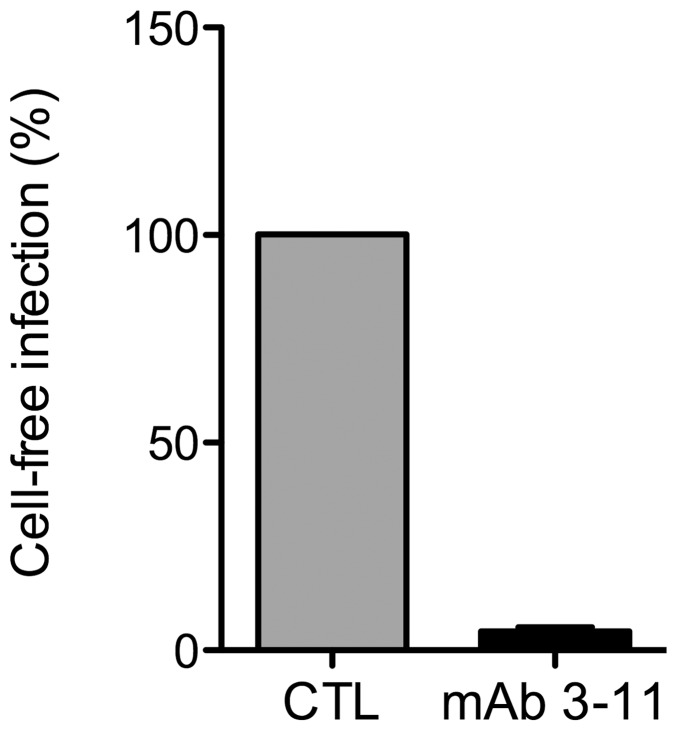
Cell-free infection of Huh-7 cells by HCVcc is blocked by the anti-E2 antibody 3/11. HCVcc were pre-incubated for 1 hr with neutralizing anti-E2 monoclonal antibody 3/11 at a concentration of 50 µg/ml and next inoculated to Huh-7 cells for 2 hr. Three days post-infection, cells were fixed, stained with the mouse anti-E1 antibody A4 and Alexa555 conjugated anti-mouse IgG, and the number of infected cells was counted. The results show the anti-E2 antibody 3/11 effectively blocks cell-free infection of Huh-7 cells by HCVcc.

**Figure 13:**
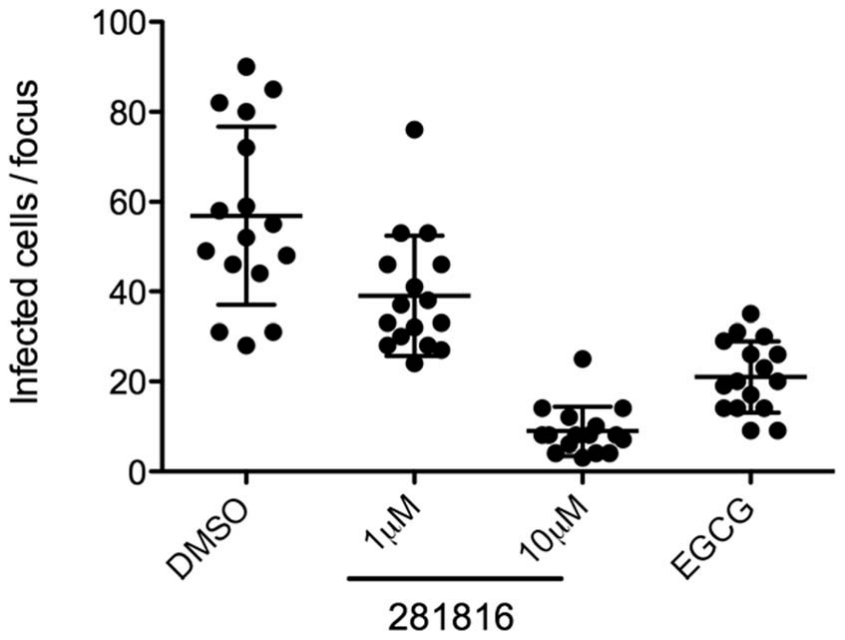
281816 blocks HCV cell-to-cell transmission. Huh-7 cells were seeded on coverslips and infected with HCVcc for 2 hr at 37°C. Cells were then washed and cultured for 72 hr at 37°C in culture medium containing the 3/11 neutralizing antibody (50 µg/ml) in presence or in absence of 281816 at indicated concentrations. Cells cultured in presence of DMSO or EGCG at 50 µM were used as controls. The number of infected cells per focus was determined by A4 indirect immunofluorescence. The results show treatment with 281816 significantly reduces the number of infected cells per focus in a dose-dependent manner. Mean p values were below 0.001 for 1 µM 281816 and below 0.0001 for the 10 µM 281816 and EGCG treatment groups.

In addition to cell-free infection, HCV can also be transmitted to neighboring cells via cell-to-cell contact by a mechanism that is not completely understood [41], [42], [62]. Indeed, HCV is transmitted in the presence of monoclonal antibodies, such as the anti-E2 antibody 3/11, or patient-derived antibodies that are able to neutralize virus-free infectivity [42], [62]. Since cell-to-cell transmission has been suggested to be a major route of transmission for HCV [41], we next analyzed the effect of 281816 on this process. For this purpose, Huh-7 cells were infected at low multiplicity of infection with HCVcc for 2 hr and then cultured with neutralizing anti-E2 antibody (3/11), which blocks infection by free particles as shown in Figure 12 [41], and in the presence of 281816 (1 µM and 10 µM). Cells cultured in the presence of 3/11 and solvent (DMSO) or Epigallocatechin-3-gallate (EGCG, 50 µM) [42] were used as negative and positive controls of inhibition, respectively. Three days post-infection, cells were fixed and foci of infected cells were visualized by immunofluorescence. Cell-to-cell transmission was measured by counting the number of infected cells per focus. The results showed that 281816 led to a significant reduction of the number of infected cells per focus in a dose-dependent manner (Figure 13). Together, these results indicate that 281816 also inhibits cell-to-cell transmission of HCV.

## Discussion

While it has been known for some time that the E2 envelope glycoprotein plays an important role in the life cycle of HCV, we are only now beginning to learn details about the structure of the E2 and how it functions. This has been attributed to the challenging intrinsic properties of the protein, such as the presence of multiple flexible loops, its tendency to form disulfide aggregates in solution and the high level of N-linked glycosylation, all of which make it difficult to determine it’s structure. Neutralizing antibody epitope analyses and mutation studies, in contrast, have provided a great deal of information about the regions of the E2 protein and specific amino acids that participate in CD81 binding and are important for HCV infectivity [9].

The recent determination of two HCV E2 protein core crystal structures [18], [19] and our use of the deposited coordinates to create a new homology model of the protein’s structure containing the majority of conserved amino acids and peptide segments known to be important for viral invasion of hepatocytes has made it possible to use computational docking and structure-based drug design methods to begin developing anti-HCV drugs that target the conserved regions of E2 and block its interaction with host receptors. Our docking of a library of diverse small molecules to this homology model led to the identification of a set of ligands that were predicted to bind to sites near key amino acids known to participate in CD81 or E1 binding or to block HCV infection, and 23 of the 34 compounds were confirmed by experiment to bind to recombinant E2 protein. When these 23 ligands were tested for activity in blocking HCV infection of Huh-7 cells, only ligand 281816 was found to inhibit HCV infection using both HCVcc and HCVpp based assays. Upon analyzing the activity spectrum of HCV using HCVpp bearing envelope proteins from different HCV genotypes (1a, 1b, 2a, 2b, 4a and 6a), 281816 was found to inhibit the infection of all tested genotypes with IC50’s ranging from 2.2 µM to 4.6 µM (Table 5), indicating that this small molecule inhibits HCV infection in a genotype-independent manner. Ligand 281816 was also observed to block the binding of HCV E2 protein to CD81-LEL protein and to Raji cells expressing CD81.

**Figure 14:**
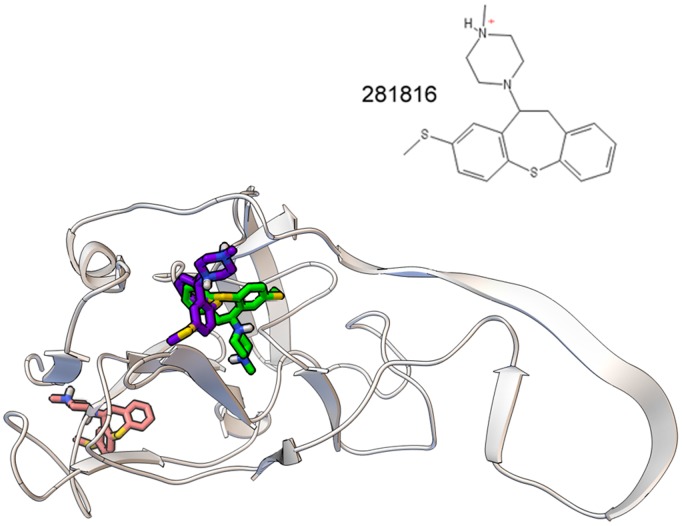
Relative location of 281816 binding sites 1 and 4 on HCV E2. 281816 (structure, top) is predicted to bind to two sites on the E2 protein. Two conformers of 281816 with the lowest free energy of binding are shown bound to site 4. The conformer with the lowest free energy of binding to site 1 is also shown. A video showing the surface structure of the E2 homology model with the three 281816 conformers bound that rotates 360° can be found in Movie S1.

The docking experiments conducted with 281816 identified the two binding sites on E2 shown in Figure 14. One cluster of 281816 conformers bound deep inside a cavity positioned directly above Y618 and P619, two amino acids in site 4 (Figure 3) that are known to contribute to E2’s binding to CD81 [47]. The two strongest 281816 ligand binding modes are shown bound to this site. 281816 was also predicted to bind to a shallow cavity on the opposite side of the protein. These conformers were predicted to bind to site 1 near residues V515, G517, P515 and H421–N423. H421–N423 is part of a larger segment of E2 that has been shown to bind to CD81 [19]. As expected, the ligand positioned above Y618 and P619 in the deeper cavity was predicted to bind more strongly to this region of the protein (free energy of binding of the best bound ligand = −8.64 kcal/mol) than when it was bound to the shallow cavity on the other side of the protein (free energy of binding = −6.39 kcal/mol).

A subset of the 281816 conformers in the cluster observed to bind near site 4 overlapped into site 2 and bound immediately adjacent to D481–P490, part of the epitope targeted by antibodies that block HCV infectivity and E2 binding to CD81 [50]. W487, a residue within this peptide segment whose mutation has been shown to disrupt E2:E1 dimerization [63], is also located near site 2. Other conformers in the cluster binding to site 1 also overlapped into site 5 and bound near amino acid residues P612 and Y613. These docking results illustrate one interesting and unique feature of the 281816 ligand; a number of its conformers are predicted to bind immediately above or next to both exposed faces of the P612–P619 amino acid residues that are known to participate in E2 binding to CD81 [47].

One factor that can have a significant impact on the accessibility of these sites to the binding of 281816 and other ligands is the oligomeric state of the native E2 protein. Analyses of the E2 protein and its complexes have shown that the protein exists in several different oligomeric states [43], [64]–[73]. These include non-covalent heterodimers of E1 and E2, large disulfide cross-linked E2 complexes and aggregates, as well as monomers and disulfide linked E2 homodimers. E1E2 non-covalent heterodimers are formed in infected cells [70]–[72], and it has been proposed that the two proteins remain as a complex on the virus surface, perhaps covalently linked through an intermolecular disulfide bond located in their transmembrane regions [69]. Large covalent complexes containing E2 stabilized by multiple disulfide bonds have also been observed to be associated with the surface of infective virions [71]. It has been hypothesized that the disulfide crosslinks in these large complexes may contribute to the structural stability of the virion. It has also been proposed that these large complexes may play a role in budding. Other large disulfide cross-linked aggregates of E2 have been found in recombinant E2 expression systems [74]–[78] and in the endoplasmic reticulum, but these aggregates do not appear to represent a functional or biologically relevant oligomeric state.

E2 is known to be heavily glycosylated [69], [79], [80]. In the HCVcc system E2 has been reported to have 11 glycosylated sites that collectively account for nearly half the mass of the protein [69], [79], [80]. Glycosylation of a number of these sites has been shown to prevent the binding of neutralizing antibodies to E2 and to block E2 binding to CD81 [81], Although the ligands identified in this study are much smaller than antibodies or CD81, the presence of the glycans could also prevent small molecules from binding to the protein’s surface if the glycosylated amino acid residues are located close to the ligand binding site.

While the oligomeric structure of E2 on the surface of the HCV virus is not known, the E2 present in the virus and pseudoparticles does bind to both recombinant CD81-LEL and the native CD81 receptor present on hepatocytes. Since each of the five ligand binding sites on E2 we used in our docking experiments contain or are located immediately adjacent to amino acid residues that are known to participate in E2 binding to CD81, ligands targeting these sites should have access to bind in the cavities. In addition, a number of these binding sites are located within epitopes recognized by antibodies that inhibit HCV infectivity, providing an additional confirmation of the accessibility of the sites. Only one of the 11 known glycosylated amino acid residues, E2N7, is located near a ligand docking site (Site 3). Our docking studies have identified 281816 ligand conformers that are predicted to bind to sites 1 and 4 and a few that overlap into sites 2 and 5, but none are expected to bind to Site 3. This suggests that the binding of 281816 and its analogs should not be affected by glycosylation. The limited proteolysis and deuterium exchange experiments conducted with the E2 protein core and reported by Khan et al. [19] also indicate that each of the five ligand docking/binding sites is accessible and exposed to solvent – an important prerequisite for ligand binding.

To probe more deeply into the inhibition of the infection process by 281816, experiments were conducted to determine if the inhibition of cell-free infection by 281816 might be limited to viral entry, which step in the entry process might be affected by the compound, and what impact, if any, 281816 might have on cell-to-cell transmission of HCV. Analyses of Huh-7 cells inoculated with HCVcc before, during or after treatment with 281816 revealed the compound only blocks HCV entry and does not inhibit post-entry processes in the HCV life cycle. A kinetic analysis of the effect of 281816, coupled with a temperature block to endocytosis and fusion, was used to examine the cell-free entry steps in more detail and showed 281816 inhibits not only the initial attachment/binding step, but it also has an effect on interactions that occur later during viral internalization and fusion. Ligand 281816 was also observed to abrogate the cell-to-cell transmission of HCV. 281816 treatment of Huh-7 cells cultured in the presence of the anti-E2 neutralizing antibody 3/11 not only led to a dose dependent reduction in the number of cells forming foci, but it was found to be more effective in blocking cell-to-cell transmission that the Epigallocatechin-3-gallate [42] used as a positive control.

The observation that some 281816 remained bound to cells after washing in the HCV entry experiments suggests that 281816 may bind to other cellular proteins. This should not be surprising, since this compound has been reported to have other activities [82]–[87]. A blind docking experiment performed with 281816 also predicted the ligand could bind to CD81. Three potential binding sites were identified, one located within the E2 binding site on CD81 and two others in regions that would not be expected to impact E2 binding. Collectively these observations suggested the exciting possibility that 281816 might play a dual role in blocking E2 binding to CD81 by binding not only to the CD81 binding site on E2, but also by binding to the E2 binding site on CD81. In support of this idea, results obtained in an SPR binding study showed 281816 bound to CD81 almost as well as it bound to E2. However, a subsequent competition experiment conducted with 281816 and a monoclonal antibody (5A6) known to bind to the E2 binding site on CD81 revealed that the ligand did not compete with the antibody. One micromolar 281816, which effectively blocks E2 binding to CD81-LEL and inhibits viral invasion of Huh-7 cells, had no effect on antibody 5A6 binding to CD81-LEL.

In addition to identifying a promising new small molecule drug lead for treating HCV that targets the E2 glycoprotein, this study also demonstrates the utility of our new E2 homology model in the discovery of small molecules that bind to important sites on E2. By targeting sites containing amino acid residues identified by others to participate in CD81 binding and CD81-dependent processes that impact HCV infectivity, a small molecule was identified that not only blocks E2 binding to CD81 and the cell-free entry process, but it is also effective in blocking the cell-to-cell transmission of HCV – the predominant mechanism of transmission that contributes to the persistence of infections [41] but for which the precise mechanism needs to be defined. Recently, it has been shown that exosomes produced by HCV infected hepatic cells can transfer viral RNA to plasmacytoid dendritic cells [88] and might transmit infection to naïve hepatic cells [89]. Although several entry factors have been implicated in this process, the viral determinants, entry factor requirements and molecular mechanisms involved in cell-to-cell transmission route still need to be further characterized. In particular, the role played by CD81 has remained controversial with studies reporting HCV cell-to-cell transmission as a CD81-dependent pathway [41], [90], [91], whereas others demonstrated a CD81-independent transmission [56], [62], [92]. However, a recent study has highlighted the coexistence of CD81-dependent and CD81-independent cell-to-cell transmission [93]. The inhibition of cell-to-cell transmission of HCV by 281816, which blocks E2 binding to CD81, is consistent with other reports of a CD81-dependent cell-to-cell transmission process [93]–[94] that can be blocked by anti-CD81 antibodies [41], [94] and soluble CD81 [56], both of which also block E2 binding to CD81. While it is possible that E2 binding to CD81 may play a role in the cell-to-cell transmission of HCV, it is also possible the 281816 that binds to CD81, which does not inhibit E2 binding, may have a totally unrelated effect that impacts the interaction of CD81 with other proteins or molecular structures in the tetraspanin web [95], [96] and blocks fusion related events involving CD81 that occur during the cell-to-cell transmission process.

281816, known as methiothepin or 1-methyl-4-(3-methylsulfanyl-5,6-dihydrobenzo[b] [1]benzothiepin-5-yl)piperazine, is also interesting because it has been determined previously to block dopamine [82] and serotonin [83] receptors and has been reported to inhibit a number of other biological activities, which include the binding or entry of two other unrelated viruses into cells (Lassa [84], Marburg [85]), *Plasmodium falciparum* proliferation [86], and *Mycoplasmodium tuberculosis* infections [87]. Numerous structural analogs of 281816 have been tested and shown to be effective in treating a wide variety of neurological diseases (schizophrenia [97], [98], Parkinson and dementia-related psychoses [99], [100]), bipolar disorders [101], [102], and depression [103], [104]. While we have not found experimental studies that report the membrane permeability of 281816, the logP (log of octanol/water partition coefficient) has been calculated to be 4.14, which indicates the compound is likely to exhibit good membrane permeability. Since the logP is less than 5, according to Chris Lipinski/Pfizer’s Rule of 5 the compound could also be orally active, as are a number of 281816 structural analogs (octoclothepine, loxapine, amoxapine, clozapine, quetiapine, olanzapine, and amitriptyline) that have been used to treat a variety of neurological disorders.

## Supporting Information

Table S1 **Table showing lowest free energies of binding obtained for all 1,715 ligands docked to the E2 homology model.**
(DOCX)

Movie S1 **Conformers of ligand 281816 exhibiting lowest free energies of binding are shown bound to sites 4 and 1 on the E2 homology model.**
(MOV)
