# Supplementary material for: Identification of a Novel Drug Lead That Inhibits HCV Infection and Cell-to-Cell Transmission by Targeting the HCV E2 Glycoprotein
Source: PLoS One. 2014 Oct 30;9(10):e111333. doi: 10.1371/journal.pone.0111333 (PMC4214736; doi:10.1371/journal.pone.0111333)
Supplement: Table S1 — Table showing lowest free energies of binding obtained for all 1,715 ligands docked to the E2 homology model. (DOCX) [file pone.0111333.s001.docx]

| Ligand (ZINC ID) | Box 1 | | Box 2 | | Box 3 | | Box 4 | |
| --- | --- | --- | --- | --- | --- | --- | --- | --- |
|  | Free Energy of Binding (kcal/mol) | Ligand Efficiency (kcal/mol/heavy atom) | Free Energy of Binding (kcal/mol) | Ligand Efficiency (kcal/mol/heavy atom) | Free Energy of Binding (kcal/mol) | Ligand Efficiency (kcal/mol/heavy atom) | Free Energy of Binding (kcal/mol) | Ligand Efficiency (kcal/mol/heavy atom) |
| ZINC00000171 | -7.5 | -0.326 | -6.2 | -0.27 | -6.2 | -0.27 | -6.3 | -0.274 |
| ZINC00000179 | -7.4 | -0.352 | -6.6 | -0.314 | -5.9 | -0.281 | -5.9 | -0.281 |
| ZINC00000570 | -8.2 | -0.41 | -6 | -0.3 | -6 | -0.3 | -5.7 | -0.285 |
| ZINC00001018 | -7.8 | -0.411 | -6.4 | -0.337 | -6.2 | -0.326 | -6.1 | -0.321 |
| ZINC00001087 | -7.8 | -0.3 | -7.7 | -0.296 | -7.1 | -0.273 | -6.9 | -0.265 |
| ZINC00001228 | -6.1 | -0.29 | -6.4 | -0.305 | -6.3 | -0.3 | -6.1 | -0.29 |
| ZINC00001522 | -6.3 | -0.45 | -5.6 | -0.4 | -5.2 | -0.371 | -5.9 | -0.421 |
| ZINC00001785 | -8.7 | -0.435 | -6.7 | -0.335 | -6.2 | -0.31 | -5.9 | -0.295 |
| ZINC00001791 | -7.9 | -0.359 | -6.8 | -0.309 | -6.5 | -0.295 | -6.1 | -0.277 |
| ZINC00001947 | -8.5 | -0.472 | -6.9 | -0.383 | -6.9 | -0.383 | -6 | -0.333 |
| ZINC00002036 | -7.8 | -0.433 | -6.4 | -0.356 | -6.1 | -0.339 | -5.8 | -0.322 |
| ZINC00002106 | -7.8 | -0.371 | -6.8 | -0.324 | -6.5 | -0.31 | -6 | -0.286 |
| ZINC00005155 | -7.9 | -0.439 | -6.6 | -0.367 | -6 | -0.333 | -5.9 | -0.328 |
| ZINC00013246 | -6.1 | -0.555 | -5.3 | -0.482 | -5 | -0.455 | -5.3 | -0.482 |
| ZINC00014894 | -6.6 | -0.44 | -5.8 | -0.387 | -5.2 | -0.347 | -5.6 | -0.373 |
| ZINC00021557 | -6.6 | -0.347 | -6.1 | -0.321 | -5.9 | -0.311 | -5.9 | -0.311 |
| ZINC00024336 | -8.2 | -0.373 | -7.1 | -0.323 | -7.2 | -0.327 | -6.6 | -0.3 |
| ZINC00028671 | -7.5 | -0.395 | -7.2 | -0.379 | -6.5 | -0.342 | -5.7 | -0.3 |
| ZINC00031164 | -6.2 | -0.517 | -5.4 | -0.45 | -4.7 | -0.392 | -4.7 | -0.392 |
| ZINC00031165 | -6.1 | -0.508 | -5.3 | -0.442 | -4.6 | -0.383 | -4.7 | -0.392 |
| ZINC00031410 | -8.7 | -0.435 | -7.3 | -0.365 | -7 | -0.35 | -7.2 | -0.36 |
| ZINC00035663 | -8.6 | -0.374 | -6.2 | -0.27 | -6.2 | -0.27 | -6 | -0.261 |
| ZINC00035871 | -8.8 | -0.419 | -7.2 | -0.343 | -6.7 | -0.319 | -6.5 | -0.31 |
| ZINC00039221 | -8.4 | -0.35 | -7.3 | -0.304 | -7.3 | -0.304 | -7.1 | -0.296 |
| ZINC00039435 | -7.4 | -0.352 | -7.2 | -0.343 | -6.7 | -0.319 | -6.2 | -0.295 |
| ZINC00039490 | -5.6 | -0.56 | -4.6 | -0.46 | -4.3 | -0.43 | -4.4 | -0.44 |
| ZINC00041118_1 | -7.9 | -0.465 | -6.5 | -0.382 | -6 | -0.353 | -6.3 | -0.371 |
| ZINC00041118 | -8.4 | -0.494 | -6.2 | -0.365 | -6.1 | -0.359 | -6.3 | -0.371 |
| ZINC00054469 | -7.1 | -0.507 | -6.1 | -0.436 | -6.2 | -0.443 | -6.2 | -0.443 |
| ZINC00056624 | -7.3 | -0.406 | -6.2 | -0.344 | -6.2 | -0.344 | -5.9 | -0.328 |
| ZINC00057263 | -8.1 | -0.426 | -6.4 | -0.337 | -6.4 | -0.337 | -6.3 | -0.332 |
| ZINC00057264 | -8.5 | -0.447 | -6.2 | -0.326 | -6.2 | -0.326 | -5.9 | -0.311 |
| ZINC00057677 | -8.5 | -0.472 | -6.7 | -0.372 | -6.5 | -0.361 | -6.2 | -0.344 |
| ZINC00057716 | -7.2 | -0.424 | -6.7 | -0.394 | -6.1 | -0.359 | -5.3 | -0.312 |
| ZINC00058294 | -7 | -0.28 | -6.9 | -0.276 | -5.9 | -0.236 | -6 | -0.24 |
| ZINC00061529 | -8.9 | -0.468 | -7.2 | -0.379 | -7.2 | -0.379 | -6.7 | -0.353 |
| ZINC00065175_1 | -6.6 | -0.388 | -6.4 | -0.376 | -5.5 | -0.324 | -5.5 | -0.324 |
| ZINC00065175 | -7.4 | -0.435 | -6.6 | -0.388 | -5.6 | -0.329 | -5.6 | -0.329 |
| ZINC00065182 | -7.2 | -0.514 | -6.1 | -0.436 | -5.7 | -0.407 | -5.2 | -0.371 |
| ZINC00066629 | -8.1 | -0.405 | -6.7 | -0.335 | -6.6 | -0.33 | -6.7 | -0.335 |
| ZINC00068268 | -8.6 | -0.358 | -6.8 | -0.283 | -6.6 | -0.275 | -6.4 | -0.267 |
| ZINC00073731 | -7.2 | -0.48 | -5.8 | -0.387 | -5.7 | -0.38 | -5.5 | -0.367 |
| ZINC00075241 | -7 | -0.467 | -6.2 | -0.413 | -5.4 | -0.36 | -6.1 | -0.407 |
| ZINC00076793 | -7.2 | -0.514 | -6.2 | -0.443 | -5.8 | -0.414 | -5.6 | -0.4 |
| ZINC00080747 | -6.8 | -0.4 | -6.4 | -0.376 | -5.6 | -0.329 | -5.6 | -0.329 |
| ZINC00081505 | -8.2 | -0.456 | -6 | -0.333 | -5.7 | -0.317 | -5.9 | -0.328 |
| ZINC00081510 | -8.2 | -0.456 | -6.7 | -0.372 | -6 | -0.333 | -6 | -0.333 |
| ZINC00084613 | -7.9 | -0.316 | -7.1 | -0.284 | -7.9 | -0.316 | -6.8 | -0.272 |
| ZINC00084617 | -8.2 | -0.328 | -7.4 | -0.296 | -6.7 | -0.268 | -6.6 | -0.264 |
| ZINC00084716 | -8.8 | -0.463 | -6.7 | -0.353 | -6.5 | -0.342 | -6.2 | -0.326 |
| ZINC00085895 | -7.5 | -0.375 | -7.1 | -0.355 | -7.1 | -0.355 | -6.7 | -0.335 |
| ZINC00086102 | -7.9 | -0.329 | -7.2 | -0.3 | -7.1 | -0.296 | -6.7 | -0.279 |
| ZINC00091200 | -7.6 | -0.422 | -6.5 | -0.361 | -6.5 | -0.361 | -5.9 | -0.328 |
| ZINC00091560 | -7.5 | -0.395 | -6.6 | -0.347 | -7 | -0.368 | -5.9 | -0.311 |
| ZINC00092157_1 | -7.9 | -0.494 | -6.3 | -0.394 | -6.3 | -0.394 | -5.8 | -0.362 |
| ZINC00092157 | -8.2 | -0.512 | -6.3 | -0.394 | -6.3 | -0.394 | -6.1 | -0.381 |
| ZINC00093738_1 | -7.8 | -0.371 | -6.3 | -0.3 | -6.3 | -0.3 | -5.8 | -0.276 |
| ZINC00093738 | -8.3 | -0.395 | -6.3 | -0.3 | -6.4 | -0.305 | -5.9 | -0.281 |
| ZINC00094441 | -7.5 | -0.375 | -6.4 | -0.32 | -6.5 | -0.325 | -6.1 | -0.305 |
| ZINC00095158 | -6.9 | -0.431 | -6.4 | -0.4 | -6.4 | -0.4 | -5.7 | -0.356 |
| ZINC00099494 | -7.7 | -0.405 | -6.1 | -0.321 | -5.6 | -0.295 | -5.5 | -0.289 |
| ZINC00099827 | -7.1 | -0.473 | -5.5 | -0.367 | -5 | -0.333 | -5.3 | -0.353 |
| ZINC00100248 | -7.6 | -0.4 | -6.9 | -0.363 | -6.8 | -0.358 | -6 | -0.316 |
| ZINC00102611 | -5 | -0.556 | -4.6 | -0.511 | -4.2 | -0.467 | -4.7 | -0.522 |
| ZINC00104475 | -7.4 | -0.493 | -5.8 | -0.387 | -5 | -0.333 | -5.6 | -0.373 |
| ZINC00105181 | -6.3 | -0.332 | -6.2 | -0.326 | -6.1 | -0.321 | -5.7 | -0.3 |
| ZINC00105309 | -8.3 | -0.319 | -7.4 | -0.285 | -6.9 | -0.265 | -7.5 | -0.288 |
| ZINC00110358_1 | -7.5 | -0.375 | -6 | -0.3 | -5.7 | -0.285 | -5.7 | -0.285 |
| ZINC00110358 | -7.8 | -0.39 | -6 | -0.3 | -5.7 | -0.285 | -5.5 | -0.275 |
| ZINC00110365_1 | -7.2 | -0.36 | -6 | -0.3 | -5.4 | -0.27 | -6.1 | -0.305 |
| ZINC00110365 | -7.7 | -0.385 | -6 | -0.3 | -5.3 | -0.265 | -5.7 | -0.285 |
| ZINC00113222 | -7.2 | -0.45 | -5.9 | -0.369 | -5.9 | -0.369 | -5.9 | -0.369 |
| ZINC00113984 | -6.6 | -0.471 | -5.2 | -0.371 | -4.9 | -0.35 | -4.9 | -0.35 |
| ZINC00115489 | -7.9 | -0.395 | -6.4 | -0.32 | -6.2 | -0.31 | -5.4 | -0.27 |
| ZINC00116869 | -7.5 | -0.417 | -5.7 | -0.317 | -5.6 | -0.311 | -5.3 | -0.294 |
| ZINC00117074 | -7.3 | -0.384 | -6.4 | -0.337 | -5.9 | -0.311 | -6.2 | -0.326 |
| ZINC00119004 | -8.2 | -0.39 | -6.2 | -0.295 | -6.1 | -0.29 | -6.4 | -0.305 |
| ZINC00119372 | -8.4 | -0.35 | -6.9 | -0.288 | -6.8 | -0.283 | -6 | -0.25 |
| ZINC00121415 | -7.1 | -0.355 | -6.3 | -0.315 | -5.7 | -0.285 | -5.3 | -0.265 |
| ZINC00126096 | -7.2 | -0.424 | -5.8 | -0.341 | -5.6 | -0.329 | -5.3 | -0.312 |
| ZINC00135449 | -6.8 | -0.283 | -7.3 | -0.304 | -5.9 | -0.246 | -5.9 | -0.246 |
| ZINC00135453 | -6.7 | -0.279 | -7.3 | -0.304 | -6.1 | -0.254 | -6.2 | -0.258 |
| ZINC00135673 | -7.2 | -0.45 | -5.8 | -0.362 | -5.5 | -0.344 | -5.7 | -0.356 |
| ZINC00136890 | -6.8 | -0.309 | -6.2 | -0.282 | -6.2 | -0.282 | -5.1 | -0.232 |
| ZINC00136892 | -7.7 | -0.35 | -6.2 | -0.282 | -6 | -0.273 | -5.6 | -0.255 |
| ZINC00136893 | -7.1 | -0.323 | -6.2 | -0.282 | -5.9 | -0.268 | -5.5 | -0.25 |
| ZINC00136894 | -7 | -0.318 | -5.9 | -0.268 | -6.1 | -0.277 | -5.6 | -0.255 |
| ZINC00137148 | -7.8 | -0.371 | -7.1 | -0.338 | -6.3 | -0.3 | -6.7 | -0.319 |
| ZINC00137187 | -7.5 | -0.341 | -7.9 | -0.359 | -6.5 | -0.295 | -5.7 | -0.259 |
| ZINC00137189 | -7.4 | -0.336 | -7.3 | -0.332 | -6.5 | -0.295 | -5.8 | -0.264 |
| ZINC00138096 | -8.5 | -0.405 | -6.3 | -0.3 | -6.3 | -0.3 | -6.1 | -0.29 |
| ZINC00138986 | -7.6 | -0.422 | -6.2 | -0.344 | -5.7 | -0.317 | -5.3 | -0.294 |
| ZINC00139367 | -6.3 | -0.45 | -5.4 | -0.386 | -5 | -0.357 | -5 | -0.357 |
| ZINC00139370 | -6.4 | -0.457 | -5.5 | -0.393 | -5 | -0.357 | -5.1 | -0.364 |
| ZINC00141916 | -7.4 | -0.322 | -6.4 | -0.278 | -6 | -0.261 | -5.8 | -0.252 |
| ZINC00143079 | -7.2 | -0.4 | -6 | -0.333 | -6.2 | -0.344 | -5.9 | -0.328 |
| ZINC00143743 | -5.5 | -0.5 | -4.9 | -0.445 | -4.4 | -0.4 | -5 | -0.455 |
| ZINC00145517 | -8.3 | -0.377 | -6.8 | -0.309 | -6.8 | -0.309 | -6.8 | -0.309 |
| ZINC00150151 | -8.1 | -0.426 | -6.1 | -0.321 | -5.4 | -0.284 | -5.3 | -0.279 |
| ZINC00151887_1 | -7.2 | -0.48 | -6 | -0.4 | -5.8 | -0.387 | -5.7 | -0.38 |
| ZINC00151887 | -7.6 | -0.507 | -5.9 | -0.393 | -5.8 | -0.387 | -5.6 | -0.373 |
| ZINC00154608 | -8.1 | -0.506 | -6.1 | -0.381 | -6 | -0.375 | -6.1 | -0.381 |
| ZINC00154792 | -6.1 | -0.508 | -5.5 | -0.458 | -5 | -0.417 | -5 | -0.417 |
| ZINC00154832 | -7.1 | -0.507 | -6.1 | -0.436 | -5.2 | -0.371 | -5.6 | -0.4 |
| ZINC00154888 | -8.2 | -0.357 | -8.5 | -0.37 | -6.7 | -0.291 | -6.7 | -0.291 |
| ZINC00155089 | -7.1 | -0.444 | -6.2 | -0.388 | -5.6 | -0.35 | -6.1 | -0.381 |
| ZINC00155292 | -7.9 | -0.395 | -7 | -0.35 | -7 | -0.35 | -6.4 | -0.32 |
| ZINC00155857 | -6.2 | -0.564 | -4.9 | -0.445 | -4.8 | -0.436 | -5.4 | -0.491 |
| ZINC00156701 | -8.7 | -0.435 | -6.7 | -0.335 | -6.1 | -0.305 | -6.1 | -0.305 |
| ZINC00157210 | -6.6 | -0.44 | -5.6 | -0.373 | -5.1 | -0.34 | -5.6 | -0.373 |
| ZINC00157625 | -7.2 | -0.48 | -6.6 | -0.44 | -5.6 | -0.373 | -5.5 | -0.367 |
| ZINC00160306 | -6.5 | -0.464 | -5.8 | -0.414 | -5.5 | -0.393 | -5.4 | -0.386 |
| ZINC00160781 | -7.4 | -0.411 | -6.2 | -0.344 | -5.6 | -0.311 | -5.6 | -0.311 |
| ZINC00161631 | -5.7 | -0.518 | -4.6 | -0.418 | -4.4 | -0.4 | -4.9 | -0.445 |
| ZINC00161700 | -7.5 | -0.3 | -7.4 | -0.296 | -7 | -0.28 | -6.9 | -0.276 |
| ZINC00163247 | -5.7 | -0.57 | -4.6 | -0.46 | -4.5 | -0.45 | -5 | -0.5 |
| ZINC00163657 | -7.7 | -0.405 | -6 | -0.316 | -6 | -0.316 | -6.1 | -0.321 |
| ZINC00166215 | -8.3 | -0.346 | -7 | -0.292 | -7.4 | -0.308 | -7 | -0.292 |
| ZINC00188345 | -9 | -0.429 | -6.8 | -0.324 | -6.5 | -0.31 | -6.4 | -0.305 |
| ZINC00204666 | -7.1 | -0.418 | -5.3 | -0.312 | -5.3 | -0.312 | -5.2 | -0.306 |
| ZINC00214590 | -7.9 | -0.395 | -6.9 | -0.345 | -6.4 | -0.32 | -6.3 | -0.315 |
| ZINC00217297 | -7.2 | -0.424 | -6.4 | -0.376 | -6.2 | -0.365 | -5.9 | -0.347 |
| ZINC00225990 | -7.5 | -0.441 | -6.1 | -0.359 | -6.2 | -0.365 | -5.7 | -0.335 |
| ZINC00226959 | -7.8 | -0.52 | -5.7 | -0.38 | -5.7 | -0.38 | -5.8 | -0.387 |
| ZINC00227103 | -8.3 | -0.361 | -6.9 | -0.3 | -6.5 | -0.283 | -6 | -0.261 |
| ZINC00235625 | -8.1 | -0.426 | -6.3 | -0.332 | -5.7 | -0.3 | -6.1 | -0.321 |
| ZINC00235981 | -7.7 | -0.513 | -5.9 | -0.393 | -5.6 | -0.373 | -5.9 | -0.393 |
| ZINC00247785 | -8.2 | -0.357 | -7.2 | -0.313 | -7 | -0.304 | -7.1 | -0.309 |
| ZINC00258800 | -8.3 | -0.415 | -7.2 | -0.36 | -7.2 | -0.36 | -6.7 | -0.335 |
| ZINC00270777 | -6.3 | -0.525 | -5.1 | -0.425 | -4.6 | -0.383 | -5.3 | -0.442 |
| ZINC00274634 | -6.6 | -0.471 | -5.4 | -0.386 | -5 | -0.357 | -5.2 | -0.371 |
| ZINC00274689 | -9.3 | -0.358 | -7.2 | -0.277 | -7.1 | -0.273 | -6.1 | -0.235 |
| ZINC00282116 | -6.6 | -0.33 | -6 | -0.3 | -5.6 | -0.28 | -5.1 | -0.255 |
| ZINC00283058 | -7.2 | -0.327 | -7.5 | -0.341 | -6.6 | -0.3 | -6.3 | -0.286 |
| ZINC00292958 | -7.3 | -0.406 | -6.5 | -0.361 | -5.4 | -0.3 | -5.2 | -0.289 |
| ZINC00293214 | -6.8 | -0.486 | -5.6 | -0.4 | -5.4 | -0.386 | -5.4 | -0.386 |
| ZINC00304325 | -8.8 | -0.4 | -6.6 | -0.3 | -6.4 | -0.291 | -6.1 | -0.277 |
| ZINC00306008 | -7.4 | -0.435 | -6.2 | -0.365 | -6.2 | -0.365 | -5.7 | -0.335 |
| ZINC00323854 | -8.6 | -0.453 | -6.9 | -0.363 | -6.1 | -0.321 | -5.5 | -0.289 |
| ZINC00329885 | -7.3 | -0.456 | -6 | -0.375 | -5.7 | -0.356 | -5.4 | -0.338 |
| ZINC00330473 | -4.8 | -0.533 | -3.9 | -0.433 | -3.9 | -0.433 | -3.9 | -0.433 |
| ZINC00331547 | -7.4 | -0.493 | -6.1 | -0.407 | -5.4 | -0.36 | -5.2 | -0.347 |
| ZINC00331745 | -7.5 | -0.536 | -6 | -0.429 | -5.4 | -0.386 | -5.3 | -0.379 |
| ZINC00332365 | -7.7 | -0.321 | -7.1 | -0.296 | -6.9 | -0.288 | -6.5 | -0.271 |
| ZINC00337138 | -7.4 | -0.463 | -5.9 | -0.369 | -5.4 | -0.338 | -5.4 | -0.338 |
| ZINC00350319 | -7.4 | -0.411 | -6.9 | -0.383 | -6.9 | -0.383 | -6.2 | -0.344 |
| ZINC00369656 | -7.8 | -0.52 | -6.1 | -0.407 | -6.1 | -0.407 | -6.1 | -0.407 |
| ZINC00383488 | -6.5 | -0.464 | -5.8 | -0.414 | -5.7 | -0.407 | -5.1 | -0.364 |
| ZINC00383489 | -6.8 | -0.486 | -5.7 | -0.407 | -5.5 | -0.393 | -5.3 | -0.379 |
| ZINC00387464 | -8.1 | -0.45 | -6.5 | -0.361 | -5.9 | -0.328 | -5.5 | -0.306 |
| ZINC00389747 | -7.1 | -0.296 | -7.2 | -0.3 | -6.4 | -0.267 | -5.7 | -0.238 |
| ZINC00389762 | -6.1 | -0.469 | -5 | -0.385 | -4.8 | -0.369 | -4.9 | -0.377 |
| ZINC00392897 | -7.6 | -0.362 | -6.1 | -0.29 | -6.3 | -0.3 | -6.4 | -0.305 |
| ZINC00393546 | -6.9 | -0.363 | -6.2 | -0.326 | -5.8 | -0.305 | -5.9 | -0.311 |
| ZINC00393598 | -7.1 | -0.444 | -5.7 | -0.356 | -5.6 | -0.35 | -5.2 | -0.325 |
| ZINC00393599 | -7.2 | -0.45 | -5.9 | -0.369 | -5.7 | -0.356 | -5.3 | -0.331 |
| ZINC00393651 | -6.7 | -0.353 | -5.8 | -0.305 | -5.3 | -0.279 | -4.8 | -0.253 |
| ZINC00393674 | -9.4 | -0.392 | -7.4 | -0.308 | -7.6 | -0.317 | -7.6 | -0.317 |
| ZINC00393856 | -8.1 | -0.368 | -7.2 | -0.327 | -7 | -0.318 | -6.8 | -0.309 |
| ZINC00393885 | -6.6 | -0.508 | -5.6 | -0.431 | -5.3 | -0.408 | -6.5 | -0.5 |
| ZINC00394316 | -6 | -0.4 | -4.8 | -0.32 | -4.8 | -0.32 | -4.7 | -0.313 |
| ZINC00394330 | -7.8 | -0.487 | -6 | -0.375 | -5.6 | -0.35 | -5.6 | -0.35 |
| ZINC00395036 | -7.9 | -0.316 | -7 | -0.28 | -7.4 | -0.296 | -6.1 | -0.244 |
| ZINC00395364 | -7 | -0.438 | -5.5 | -0.344 | -5 | -0.312 | -5.2 | -0.325 |
| ZINC00395576 | -5.7 | -0.57 | -5 | -0.5 | -4.6 | -0.46 | -5.1 | -0.51 |
| ZINC00395872 | -6.7 | -0.558 | -5.1 | -0.425 | -4.8 | -0.4 | -4.7 | -0.392 |
| ZINC00397002 | -7.8 | -0.433 | -6.3 | -0.35 | -6.2 | -0.344 | -6.1 | -0.339 |
| ZINC00397404 | -5.8 | -0.483 | -4.8 | -0.4 | -4.8 | -0.4 | -4.8 | -0.4 |
| ZINC00397978 | -7 | -0.389 | -6.2 | -0.344 | -6.1 | -0.339 | -6 | -0.333 |
| ZINC00398060 | -7.8 | -0.39 | -6.7 | -0.335 | -6.7 | -0.335 | -6.1 | -0.305 |
| ZINC00398332 | -7 | -0.467 | -5.3 | -0.353 | -5.1 | -0.34 | -5.6 | -0.373 |
| ZINC00398390 | -8.7 | -0.335 | -7.5 | -0.288 | -7.6 | -0.292 | -7.2 | -0.277 |
| ZINC00398392 | -8.2 | -0.39 | -7 | -0.333 | -7 | -0.333 | -6.7 | -0.319 |
| ZINC00398470 | -8.2 | -0.373 | -7.4 | -0.336 | -7.4 | -0.336 | -7 | -0.318 |
| ZINC00399102 | -6.4 | -0.427 | -5.4 | -0.36 | -5.2 | -0.347 | -5.1 | -0.34 |
| ZINC00399281 | -8.2 | -0.373 | -7.1 | -0.323 | -7.7 | -0.35 | -6.2 | -0.282 |
| ZINC00399404 | -6.8 | -0.309 | -6.4 | -0.291 | -6.6 | -0.3 | -5.6 | -0.255 |
| ZINC00399512 | -5.6 | -0.467 | -4.6 | -0.383 | -4.5 | -0.375 | -5.1 | -0.425 |
| ZINC00400127 | -8.4 | -0.35 | -7 | -0.292 | -7.4 | -0.308 | -6.7 | -0.279 |
| ZINC00400410 | -8.1 | -0.386 | -7.1 | -0.338 | -7.1 | -0.338 | -6.3 | -0.3 |
| ZINC00400541 | -7 | -0.467 | -5.5 | -0.367 | -5.3 | -0.353 | -5.7 | -0.38 |
| ZINC00401809 | -7.4 | -0.352 | -6.5 | -0.31 | -6.5 | -0.31 | -6.2 | -0.295 |
| ZINC00404349 | -5 | -0.556 | -4.3 | -0.478 | -4 | -0.444 | -4.8 | -0.533 |
| ZINC00404427 | -6 | -0.545 | -5.2 | -0.473 | -4.8 | -0.436 | -4.9 | -0.445 |
| ZINC00404472 | -6.4 | -0.337 | -6.3 | -0.332 | -6.4 | -0.337 | -5.9 | -0.311 |
| ZINC00407984 | -6.5 | -0.342 | -6.2 | -0.326 | -6.1 | -0.321 | -6 | -0.316 |
| ZINC00408303 | -7.3 | -0.487 | -5.3 | -0.353 | -5.1 | -0.34 | -5.5 | -0.367 |
| ZINC00408598 | -7.1 | -0.374 | -6.9 | -0.363 | -6.8 | -0.358 | -6.4 | -0.337 |
| ZINC00409811 | -7.2 | -0.36 | -5.9 | -0.295 | -5.7 | -0.285 | -5.5 | -0.275 |
| ZINC00409897 | -8.2 | -0.432 | -6.2 | -0.326 | -6 | -0.316 | -5.9 | -0.311 |
| ZINC00410187 | -7 | -0.368 | -7.4 | -0.389 | -6.2 | -0.326 | -6.3 | -0.332 |
| ZINC00410188 | -7.2 | -0.379 | -7.5 | -0.395 | -6.8 | -0.358 | -6.3 | -0.332 |
| ZINC00410244 | -6.9 | -0.46 | -5.8 | -0.387 | -5.4 | -0.36 | -5.4 | -0.36 |
| ZINC00410252 | -7.3 | -0.429 | -6.3 | -0.371 | -6.3 | -0.371 | -5.9 | -0.347 |
| ZINC00410253 | -6.8 | -0.523 | -5.4 | -0.415 | -5 | -0.385 | -5.3 | -0.408 |
| ZINC00410269 | -8.6 | -0.391 | -6.6 | -0.3 | -6.6 | -0.3 | -5.6 | -0.255 |
| ZINC00410312 | -6.9 | -0.431 | -5.6 | -0.35 | -5.6 | -0.35 | -5.4 | -0.338 |
| ZINC00433226 | -7.7 | -0.428 | -6.3 | -0.35 | -6.3 | -0.35 | -5.8 | -0.322 |
| ZINC00434844 | -7.6 | -0.475 | -5.8 | -0.362 | -5.4 | -0.338 | -5.3 | -0.331 |
| ZINC00442499 | -6.9 | -0.46 | -5.9 | -0.393 | -5.9 | -0.393 | -5.1 | -0.34 |
| ZINC00444253 | -6.3 | -0.45 | -5 | -0.357 | -4.7 | -0.336 | -4.6 | -0.329 |
| ZINC00475774 | -6.8 | -0.4 | -5.8 | -0.341 | -5.3 | -0.312 | -5.3 | -0.312 |
| ZINC00513614 | -7.9 | -0.376 | -6.9 | -0.329 | -6.9 | -0.329 | -6.2 | -0.295 |
| ZINC00538110 | -6.4 | -0.183 | -7 | -0.2 | -7.1 | -0.203 | -7.5 | -0.214 |
| ZINC00563913 | -7.9 | -0.465 | -6.2 | -0.365 | -6.3 | -0.371 | -5.9 | -0.347 |
| ZINC00600322 | -7.6 | -0.281 | -7.4 | -0.274 | -6.8 | -0.252 | -6.8 | -0.252 |
| ZINC00601312 | -7.6 | -0.33 | -6.5 | -0.283 | -6.5 | -0.283 | -6.3 | -0.274 |
| ZINC00607731 | -7.7 | -0.321 | -7.2 | -0.3 | -6.9 | -0.288 | -6.7 | -0.279 |
| ZINC00608128 | -6.8 | -0.262 | -7.5 | -0.288 | -5.8 | -0.223 | -5.8 | -0.223 |
| ZINC00608205 | -8.1 | -0.368 | -7 | -0.318 | -6.2 | -0.282 | -5.8 | -0.264 |
| ZINC00611334 | -6.5 | -0.295 | -6.3 | -0.286 | -5.9 | -0.268 | -5.5 | -0.25 |
| ZINC00611335 | -7.6 | -0.345 | -6.2 | -0.282 | -6 | -0.273 | -5.6 | -0.255 |
| ZINC00615883 | -8.5 | -0.37 | -7.2 | -0.313 | -7.1 | -0.309 | -6.8 | -0.296 |
| ZINC00625965 | -8.2 | -0.328 | -6.6 | -0.264 | -6.7 | -0.268 | -7.2 | -0.288 |
| ZINC00639634 | -6.6 | -0.254 | -6 | -0.231 | -5.8 | -0.223 | -5.8 | -0.223 |
| ZINC00640726 | -7.9 | -0.293 | -7.2 | -0.267 | -7.5 | -0.278 | -6.3 | -0.233 |
| ZINC00640777 | -7.9 | -0.263 | -6.9 | -0.23 | -6.8 | -0.227 | -6.3 | -0.21 |
| ZINC00641093 | -8.5 | -0.315 | -7.3 | -0.27 | -7.2 | -0.267 | -6.6 | -0.244 |
| ZINC00641146 | -8 | -0.32 | -6.2 | -0.248 | -7 | -0.28 | -7 | -0.28 |
| ZINC00642588 | -8.6 | -0.374 | -6.7 | -0.291 | -7.2 | -0.313 | -6.7 | -0.291 |
| ZINC00728291 | -6.6 | -0.236 | -7.4 | -0.264 | -6.4 | -0.229 | -6.9 | -0.246 |
| ZINC00810178 | -5.9 | -0.492 | -4.9 | -0.408 | -4.8 | -0.4 | -5.1 | -0.425 |
| ZINC00815686 | -6.5 | -0.406 | -5.6 | -0.35 | -5.6 | -0.35 | -5.4 | -0.338 |
| ZINC00838233 | -7.2 | -0.4 | -6.1 | -0.339 | -5.8 | -0.322 | -5.7 | -0.317 |
| ZINC00849668 | -7.3 | -0.429 | -6.4 | -0.376 | -5.6 | -0.329 | -6.9 | -0.406 |
| ZINC00896836 | -8.4 | -0.42 | -5.9 | -0.295 | -6 | -0.3 | -5.7 | -0.285 |
| ZINC00900951 | -7.3 | -0.348 | -7.2 | -0.343 | -6.3 | -0.3 | -6 | -0.286 |
| ZINC00967339 | -7.5 | -0.536 | -6.3 | -0.45 | -5.5 | -0.393 | -5.6 | -0.4 |
| ZINC00968360 | -6.8 | -0.296 | -6.7 | -0.291 | -5.9 | -0.257 | -6 | -0.261 |
| ZINC00977107 | -6.6 | -0.508 | -5.8 | -0.446 | -5.2 | -0.4 | -5.4 | -0.415 |
| ZINC00984004 | -7.4 | -0.389 | -6.1 | -0.321 | -5.7 | -0.3 | -5.8 | -0.305 |
| ZINC00990239 | -7 | -0.25 | -8.3 | -0.296 | -7.6 | -0.271 | -7.7 | -0.275 |
| ZINC01036792 | -7.8 | -0.52 | -6.7 | -0.447 | -5.9 | -0.393 | -5.8 | -0.387 |
| ZINC01036880 | -7.9 | -0.359 | -6.9 | -0.314 | -6.2 | -0.282 | -5.7 | -0.259 |
| ZINC01045051 | -8 | -0.348 | -6.3 | -0.274 | -6.2 | -0.27 | -5.6 | -0.243 |
| ZINC01045089 | -7.7 | -0.296 | -7.3 | -0.281 | -7.6 | -0.292 | -7.5 | -0.288 |
| ZINC01045090 | -6.8 | -0.262 | -7.6 | -0.292 | -7.4 | -0.285 | -7.2 | -0.277 |
| ZINC01045530 | -7.6 | -0.292 | -7.2 | -0.277 | -7.1 | -0.273 | -6.6 | -0.254 |
| ZINC01045531 | -8.6 | -0.331 | -7 | -0.269 | -7 | -0.269 | -6.5 | -0.25 |
| ZINC01069082 | -6.7 | -0.258 | -7.4 | -0.285 | -6.5 | -0.25 | -6.6 | -0.254 |
| ZINC01081577 | -7.8 | -0.312 | -6.9 | -0.276 | -6.9 | -0.276 | -6.5 | -0.26 |
| ZINC01082213 | -7.8 | -0.487 | -6 | -0.375 | -5.7 | -0.356 | -6.2 | -0.388 |
| ZINC01163259 | -8.6 | -0.331 | -7.8 | -0.3 | -7.3 | -0.281 | -7.2 | -0.277 |
| ZINC01196937 | -6.9 | -0.288 | -7.3 | -0.304 | -6.6 | -0.275 | -6.5 | -0.271 |
| ZINC01323776 | -7.8 | -0.3 | -7.2 | -0.277 | -7 | -0.269 | -6.5 | -0.25 |
| ZINC01481770 | -6.9 | -0.314 | -8.1 | -0.368 | -6.7 | -0.305 | -6.7 | -0.305 |
| ZINC01481981_1 | -6.7 | -0.447 | -5.2 | -0.347 | -4.7 | -0.313 | -5.1 | -0.34 |
| ZINC01481981 | -6.7 | -0.447 | -5.3 | -0.353 | -4.9 | -0.327 | -6 | -0.4 |
| ZINC01530795 | -6.4 | -0.427 | -5.4 | -0.36 | -5 | -0.333 | -4.7 | -0.313 |
| ZINC01530836 | -7.7 | -0.266 | -8.4 | -0.29 | -6.6 | -0.228 | -6.8 | -0.234 |
| ZINC01532548 | -6.7 | -0.419 | -5.3 | -0.331 | -5.8 | -0.362 | -5.5 | -0.344 |
| ZINC01555262 | -6.2 | -0.517 | -5.3 | -0.442 | -4.8 | -0.4 | -5 | -0.417 |
| ZINC01555547 | -7.9 | -0.395 | -7.1 | -0.355 | -6.1 | -0.305 | -6.5 | -0.325 |
| ZINC01555676 | -6.4 | -0.457 | -5.6 | -0.4 | -5.2 | -0.371 | -4.9 | -0.35 |
| ZINC01555979 | -5.4 | -0.54 | -4.6 | -0.46 | -4.1 | -0.41 | -4.2 | -0.42 |
| ZINC01556940 | -8.2 | -0.342 | -6.7 | -0.279 | -6.7 | -0.279 | -6.5 | -0.271 |
| ZINC01558226 | -6.3 | -0.394 | -5.4 | -0.338 | -5.4 | -0.338 | -5 | -0.312 |
| ZINC01559756 | -8.3 | -0.296 | -8.1 | -0.289 | -7.4 | -0.264 | -7 | -0.25 |
| ZINC01560078 | -5.3 | -0.482 | -4.3 | -0.391 | -4.3 | -0.391 | -4.4 | -0.4 |
| ZINC01560636 | -8.5 | -0.405 | -6.7 | -0.319 | -6.5 | -0.31 | -6.3 | -0.3 |
| ZINC01561127 | -8.2 | -0.432 | -6.2 | -0.326 | -6.4 | -0.337 | -5.5 | -0.289 |
| ZINC01561462 | -7.8 | -0.459 | -6.4 | -0.376 | -6.2 | -0.365 | -6.3 | -0.371 |
| ZINC01561576 | -7.8 | -0.371 | -6.4 | -0.305 | -5.7 | -0.271 | -6.4 | -0.305 |
| ZINC01561637 | -8.2 | -0.357 | -6.2 | -0.27 | -6 | -0.261 | -5.9 | -0.257 |
| ZINC01561931 | -6.5 | -0.26 | -7.3 | -0.292 | -5.6 | -0.224 | -5.6 | -0.224 |
| ZINC01562022 | -6.2 | -0.443 | -5.1 | -0.364 | -4.7 | -0.336 | -4.9 | -0.35 |
| ZINC01562304 | -9.1 | -0.35 | -7.1 | -0.273 | -7.1 | -0.273 | -6.7 | -0.258 |
| ZINC01562340 | -6.3 | -0.394 | -5.1 | -0.319 | -4.9 | -0.306 | -5.4 | -0.338 |
| ZINC01562352 | -7.7 | -0.453 | -6 | -0.353 | -5.8 | -0.341 | -5.9 | -0.347 |
| ZINC01562357 | -7.5 | -0.441 | -5.8 | -0.341 | -5.6 | -0.329 | -5.4 | -0.318 |
| ZINC01562359 | -8 | -0.5 | -5.6 | -0.35 | -5.6 | -0.35 | -5.3 | -0.331 |
| ZINC01562368 | -8 | -0.421 | -6.4 | -0.337 | -6.1 | -0.321 | -5.8 | -0.305 |
| ZINC01562588 | -8 | -0.348 | -6.8 | -0.296 | -6.7 | -0.291 | -6.3 | -0.274 |
| ZINC01563104 | -7.8 | -0.371 | -6 | -0.286 | -5.5 | -0.262 | -5.6 | -0.267 |
| ZINC01563302 | -7.2 | -0.424 | -5.4 | -0.318 | -5.2 | -0.306 | -5.2 | -0.306 |
| ZINC01563324 | -7.5 | -0.242 | -7 | -0.226 | -6.2 | -0.2 | -6.2 | -0.2 |
| ZINC01563325 | -7.4 | -0.239 | -7.5 | -0.242 | -6.4 | -0.206 | -5.8 | -0.187 |
| ZINC01563326 | -7.3 | -0.235 | -7 | -0.226 | -6 | -0.194 | -5.9 | -0.19 |
| ZINC01563327 | -6.6 | -0.213 | -7.1 | -0.229 | -6 | -0.194 | -6.1 | -0.197 |
| ZINC01563973 | -7.6 | -0.345 | -6.7 | -0.305 | -6.3 | -0.286 | -5.5 | -0.25 |
| ZINC01563997 | -7.6 | -0.317 | -6.6 | -0.275 | -6.2 | -0.258 | -6.2 | -0.258 |
| ZINC01563998 | -7.5 | -0.341 | -6.3 | -0.286 | -6.1 | -0.277 | -5.9 | -0.268 |
| ZINC01564760 | -7.8 | -0.39 | -7.5 | -0.375 | -7.5 | -0.375 | -6.4 | -0.32 |
| ZINC01566093 | -9.1 | -0.396 | -7.5 | -0.326 | -7 | -0.304 | -6.9 | -0.3 |
| ZINC01566193 | -6.4 | -0.427 | -5.2 | -0.347 | -5 | -0.333 | -5.3 | -0.353 |
| ZINC01566453_1 | -8.6 | -0.41 | -6.5 | -0.31 | -6 | -0.286 | -6.1 | -0.29 |
| ZINC01566453 | -8.8 | -0.419 | -6.5 | -0.31 | -6.1 | -0.29 | -6.1 | -0.29 |
| ZINC01566685 | -6.4 | -0.376 | -5.4 | -0.318 | -5.4 | -0.318 | -4.9 | -0.288 |
| ZINC01566905 | -6.6 | -0.44 | -5.2 | -0.347 | -5 | -0.333 | -5.1 | -0.34 |
| ZINC01566917 | -7 | -0.212 | -8.2 | -0.248 | -7 | -0.212 | -6.8 | -0.206 |
| ZINC01567155 | -7.4 | -0.389 | -6.1 | -0.321 | -6.1 | -0.321 | -5.6 | -0.295 |
| ZINC01567413 | -6.2 | -0.443 | -4.9 | -0.35 | -5.1 | -0.364 | -5.1 | -0.364 |
| ZINC01567421 | -6.2 | -0.27 | -7.3 | -0.317 | -6.3 | -0.274 | -6.3 | -0.274 |
| ZINC01567747 | -7 | -0.368 | -5.2 | -0.274 | -5.4 | -0.284 | -4.7 | -0.247 |
| ZINC01568035 | -8.5 | -0.304 | -7.5 | -0.268 | -7.4 | -0.264 | -7.2 | -0.257 |
| ZINC01568661 | -7.4 | -0.411 | -6.4 | -0.356 | -6.5 | -0.361 | -5.9 | -0.328 |
| ZINC01568793 | -10 | -0.278 | -8.1 | -0.225 | -8.6 | -0.239 | -7.9 | -0.219 |
| ZINC01568966 | -8.8 | -0.314 | -7.9 | -0.282 | -7.2 | -0.257 | -7.6 | -0.271 |
| ZINC01569015 | -6.5 | -0.433 | -4.9 | -0.327 | -4.7 | -0.313 | -4.7 | -0.313 |
| ZINC01569236 | -6.7 | -0.209 | -7.9 | -0.247 | -6.7 | -0.209 | -6.6 | -0.206 |
| ZINC01569237 | -7.1 | -0.229 | -7.7 | -0.248 | -7 | -0.226 | -6.7 | -0.216 |
| ZINC01569416 | -7 | -0.28 | -6.1 | -0.244 | -6.5 | -0.26 | -6.2 | -0.248 |
| ZINC01569482 | -8.5 | -0.447 | -6.8 | -0.358 | -6.8 | -0.358 | -6.3 | -0.332 |
| ZINC01569495 | -7.2 | -0.45 | -5.9 | -0.369 | -5.8 | -0.362 | -5.4 | -0.338 |
| ZINC01569498 | -5.7 | -0.475 | -4.8 | -0.4 | -4.3 | -0.358 | -4.7 | -0.392 |
| ZINC01569586 | -6.7 | -0.447 | -5.2 | -0.347 | -4.9 | -0.327 | -5.2 | -0.347 |
| ZINC01570210 | -7.1 | -0.444 | -6.1 | -0.381 | -5.7 | -0.356 | -5.9 | -0.369 |
| ZINC01570216 | -7.8 | -0.459 | -7.3 | -0.429 | -6.5 | -0.382 | -6.1 | -0.359 |
| ZINC01570386 | -6.6 | -0.44 | -5.6 | -0.373 | -5.1 | -0.34 | -4.9 | -0.327 |
| ZINC01570468 | -6.4 | -0.305 | -6.1 | -0.29 | -5.4 | -0.257 | -5.2 | -0.248 |
| ZINC01571105 | -6.4 | -0.457 | -6.1 | -0.436 | -5.5 | -0.393 | -5.5 | -0.393 |
| ZINC01571135 | -7.5 | -0.417 | -6.4 | -0.356 | -6 | -0.333 | -5.9 | -0.328 |
| ZINC01571136 | -7.4 | -0.411 | -6.4 | -0.356 | -6.4 | -0.356 | -5.8 | -0.322 |
| ZINC01572056 | -5.8 | -0.29 | -5.3 | -0.265 | -5.2 | -0.26 | -5.3 | -0.265 |
| ZINC01572309 | -8.7 | -0.264 | -7.9 | -0.239 | -8.5 | -0.258 | -7.4 | -0.224 |
| ZINC01572767 | -7 | -0.241 | -8 | -0.276 | -6.6 | -0.228 | -6.6 | -0.228 |
| ZINC01572960 | -7.5 | -0.312 | -7.2 | -0.3 | -6.3 | -0.263 | -6.3 | -0.263 |
| ZINC01572961 | -7.8 | -0.325 | -7 | -0.292 | -6.4 | -0.267 | -6.3 | -0.263 |
| ZINC01572962 | -7.4 | -0.308 | -7 | -0.292 | -6.4 | -0.267 | -6.1 | -0.254 |
| ZINC01572963 | -7.9 | -0.329 | -7.4 | -0.308 | -6.5 | -0.271 | -6.3 | -0.263 |
| ZINC01573467 | -9.3 | -0.404 | -7.3 | -0.317 | -7.3 | -0.317 | -7 | -0.304 |
| ZINC01573517 | -6.5 | -0.241 | -7.5 | -0.278 | -6.3 | -0.233 | -6.3 | -0.233 |
| ZINC01573531 | -8 | -0.348 | -6 | -0.261 | -6.6 | -0.287 | -6.5 | -0.283 |
| ZINC01573541 | -7.1 | -0.338 | -6.2 | -0.295 | -6.2 | -0.295 | -5.8 | -0.276 |
| ZINC01573829 | -9.1 | -0.35 | -8.2 | -0.315 | -7.9 | -0.304 | -7.6 | -0.292 |
| ZINC01573978 | -9.1 | -0.433 | -7.3 | -0.348 | -6.9 | -0.329 | -6.4 | -0.305 |
| ZINC01574246 | -6.8 | -0.378 | -6.9 | -0.383 | -6.9 | -0.383 | -6.3 | -0.35 |
| ZINC01574310 | -6.9 | -0.493 | -5.3 | -0.379 | -4.9 | -0.35 | -4.9 | -0.35 |
| ZINC01574615 | -8 | -0.348 | -7.1 | -0.309 | -7.3 | -0.317 | -7.4 | -0.322 |
| ZINC01574620 | -7.7 | -0.35 | -6.4 | -0.291 | -6.5 | -0.295 | -6.4 | -0.291 |
| ZINC01574971 | -6.9 | -0.406 | -5.4 | -0.318 | -5.6 | -0.329 | -5.1 | -0.3 |
| ZINC01575013 | -6.2 | -0.477 | -5.7 | -0.438 | -5.5 | -0.423 | -5.6 | -0.431 |
| ZINC01575102 | -7.4 | -0.389 | -6.4 | -0.337 | -6.4 | -0.337 | -5.8 | -0.305 |
| ZINC01576406 | -7.2 | -0.313 | -6.1 | -0.265 | -6.2 | -0.27 | -6.4 | -0.278 |
| ZINC01577602 | -6.8 | -0.358 | -6 | -0.316 | -5.8 | -0.305 | -5.7 | -0.3 |
| ZINC01577889 | -7.4 | -0.247 | -8.2 | -0.273 | -8 | -0.267 | -6.3 | -0.21 |
| ZINC01578220 | -8.9 | -0.297 | -6.8 | -0.227 | -7.1 | -0.237 | -6.5 | -0.217 |
| ZINC01578333 | -7.6 | -0.253 | -7.3 | -0.243 | -7.4 | -0.247 | -7.4 | -0.247 |
| ZINC01579461 | -7.6 | -0.345 | -6.3 | -0.286 | -5.8 | -0.264 | -5.7 | -0.259 |
| ZINC01579687 | -6.7 | -0.479 | -5.4 | -0.386 | -5.1 | -0.364 | -5.1 | -0.364 |
| ZINC01579723 | -7.1 | -0.418 | -5.9 | -0.347 | -5.6 | -0.329 | -5.6 | -0.329 |
| ZINC01579734 | -7.3 | -0.429 | -5.8 | -0.341 | -5.5 | -0.324 | -5.6 | -0.329 |
| ZINC01579740 | -7.6 | -0.422 | -6.3 | -0.35 | -6.6 | -0.367 | -5.6 | -0.311 |
| ZINC01579877 | -6.4 | -0.427 | -4.9 | -0.327 | -4.5 | -0.3 | -5.2 | -0.347 |
| ZINC01580992 | -9.1 | -0.379 | -8.4 | -0.35 | -7.4 | -0.308 | -7.4 | -0.308 |
| ZINC01581342 | -8.8 | -0.463 | -6.2 | -0.326 | -6.1 | -0.321 | -5.7 | -0.3 |
| ZINC01581709 | -6.4 | -0.457 | -5.4 | -0.386 | -5.2 | -0.371 | -5.6 | -0.4 |
| ZINC01582372 | -8.1 | -0.368 | -6.6 | -0.3 | -6.7 | -0.305 | -6.3 | -0.286 |
| ZINC01583123 | -6.9 | -0.493 | -5.3 | -0.379 | -5.3 | -0.379 | -5.3 | -0.379 |
| ZINC01583649 | -7.6 | -0.447 | -6.2 | -0.365 | -5.4 | -0.318 | -5.4 | -0.318 |
| ZINC01583786 | -6.8 | -0.378 | -6.8 | -0.378 | -6 | -0.333 | -6.4 | -0.356 |
| ZINC01584497 | -8.7 | -0.322 | -7.3 | -0.27 | -7.2 | -0.267 | -7.2 | -0.267 |
| ZINC01584862 | -7.6 | -0.4 | -6 | -0.316 | -6 | -0.316 | -5.8 | -0.305 |
| ZINC01585729 | -6.3 | -0.286 | -7.2 | -0.327 | -6.2 | -0.282 | -5.7 | -0.259 |
| ZINC01586128 | -7.5 | -0.326 | -6.3 | -0.274 | -6.4 | -0.278 | -6.5 | -0.283 |
| ZINC01586252 | -9.1 | -0.379 | -7.3 | -0.304 | -7 | -0.292 | -6.9 | -0.288 |
| ZINC01586330 | -7.8 | -0.371 | -6.7 | -0.319 | -6.6 | -0.314 | -6.1 | -0.29 |
| ZINC01586453 | -7.3 | -0.487 | -6.3 | -0.42 | -6.1 | -0.407 | -6.1 | -0.407 |
| ZINC01587328 | -6.4 | -0.492 | -5.2 | -0.4 | -4.5 | -0.346 | -5.1 | -0.392 |
| ZINC01587338_1 | -5.6 | -0.509 | -4.5 | -0.409 | -4.4 | -0.4 | -5.1 | -0.464 |
| ZINC01587338 | -5.5 | -0.5 | -4.5 | -0.409 | -4.5 | -0.409 | -5 | -0.455 |
| ZINC01588812 | -8.1 | -0.386 | -6.8 | -0.324 | -6.4 | -0.305 | -6.1 | -0.29 |
| ZINC01589060 | -7.6 | -0.447 | -6.3 | -0.371 | -6.3 | -0.371 | -5.9 | -0.347 |
| ZINC01589436 | -6.8 | -0.34 | -5.7 | -0.285 | -5 | -0.25 | -4.9 | -0.245 |
| ZINC01589497 | -6.6 | -0.3 | -7 | -0.318 | -6.3 | -0.286 | -6.2 | -0.282 |
| ZINC01589627 | -8.7 | -0.348 | -7.6 | -0.304 | -6.8 | -0.272 | -6.4 | -0.256 |
| ZINC01590480 | -9.2 | -0.368 | -7.4 | -0.296 | -6.9 | -0.276 | -6.7 | -0.268 |
| ZINC01590864 | -8.6 | -0.358 | -7 | -0.292 | -6.3 | -0.263 | -6.4 | -0.267 |
| ZINC01590927 | -6.6 | -0.254 | -7 | -0.269 | -6.6 | -0.254 | -5.6 | -0.215 |
| ZINC01591881 | -6.8 | -0.4 | -5.7 | -0.335 | -5.5 | -0.324 | -5.4 | -0.318 |
| ZINC01591915 | -7.1 | -0.296 | -6.1 | -0.254 | -6.4 | -0.267 | -6 | -0.25 |
| ZINC01591950 | -7.5 | -0.469 | -5.9 | -0.369 | -5.6 | -0.35 | -5.7 | -0.356 |
| ZINC01591957 | -6.7 | -0.353 | -6 | -0.316 | -5 | -0.263 | -5.1 | -0.268 |
| ZINC01592023 | -7.5 | -0.441 | -5.7 | -0.335 | -5.7 | -0.335 | -5.4 | -0.318 |
| ZINC01592410 | -7.4 | -0.463 | -5.7 | -0.356 | -5.4 | -0.338 | -5.3 | -0.331 |
| ZINC01592974 | -8.3 | -0.437 | -6.6 | -0.347 | -6.6 | -0.347 | -5.5 | -0.289 |
| ZINC01593556 | -8 | -0.381 | -7 | -0.333 | -7.4 | -0.352 | -7.4 | -0.352 |
| ZINC01593739 | -7.4 | -0.411 | -7 | -0.389 | -6.3 | -0.35 | -6.7 | -0.372 |
| ZINC01595620 | -6.6 | -0.471 | -6.1 | -0.436 | -5.5 | -0.393 | -5.6 | -0.4 |
| ZINC01596285 | -6.1 | -0.436 | -5.2 | -0.371 | -4.9 | -0.35 | -5.3 | -0.379 |
| ZINC01596698 | -9.2 | -0.383 | -6.8 | -0.283 | -6.6 | -0.275 | -6.9 | -0.288 |
| ZINC01597168 | -7.2 | -0.4 | -6.1 | -0.339 | -5.7 | -0.317 | -5.3 | -0.294 |
| ZINC01599183 | -8 | -0.364 | -6.5 | -0.295 | -6.1 | -0.277 | -5.6 | -0.255 |
| ZINC01601483 | -7.8 | -0.339 | -6.9 | -0.3 | -6.1 | -0.265 | -6 | -0.261 |
| ZINC01602509 | -8.2 | -0.456 | -6.8 | -0.378 | -6.4 | -0.356 | -5.6 | -0.311 |
| ZINC01602510 | -7.5 | -0.441 | -6.2 | -0.365 | -6.2 | -0.365 | -5.8 | -0.341 |
| ZINC01602855 | -7.2 | -0.4 | -5.9 | -0.328 | -5.4 | -0.3 | -5.2 | -0.289 |
| ZINC01602856 | -7.5 | -0.395 | -5.6 | -0.295 | -5.4 | -0.284 | -5.3 | -0.279 |
| ZINC01602938 | -5.9 | -0.536 | -4.6 | -0.418 | -4.6 | -0.418 | -4.7 | -0.427 |
| ZINC01603961 | -8.2 | -0.373 | -6.6 | -0.3 | -6.4 | -0.291 | -6.1 | -0.277 |
| ZINC01604104 | -4.4 | -0.4 | -3.6 | -0.327 | -3.5 | -0.318 | -3.8 | -0.345 |
| ZINC01604214 | -7.4 | -0.336 | -7.3 | -0.332 | -6.8 | -0.309 | -6.5 | -0.295 |
| ZINC01605775 | -7.7 | -0.385 | -5.7 | -0.285 | -5.7 | -0.285 | -5 | -0.25 |
| ZINC01606051 | -6.1 | -0.469 | -5.4 | -0.415 | -4.7 | -0.362 | -4.9 | -0.377 |
| ZINC01607786 | -9.2 | -0.368 | -7.5 | -0.3 | -7.5 | -0.3 | -7 | -0.28 |
| ZINC01608678 | -7.8 | -0.487 | -5.9 | -0.369 | -5.5 | -0.344 | -6.2 | -0.388 |
| ZINC01608684 | -6.1 | -0.469 | -5 | -0.385 | -4.7 | -0.362 | -4.9 | -0.377 |
| ZINC01608764 | -6.5 | -0.433 | -5.8 | -0.387 | -5.3 | -0.353 | -5.2 | -0.347 |
| ZINC01608817 | -8.7 | -0.362 | -6.8 | -0.283 | -7.1 | -0.296 | -7.1 | -0.296 |
| ZINC01608819 | -7.4 | -0.435 | -6.6 | -0.388 | -6.6 | -0.388 | -6.3 | -0.371 |
| ZINC01609078 | -7.2 | -0.45 | -6.3 | -0.394 | -5.9 | -0.369 | -6 | -0.375 |
| ZINC01609695 | -6.6 | -0.347 | -6.3 | -0.332 | -6 | -0.316 | -5.6 | -0.295 |
| ZINC01615121 | -7.2 | -0.36 | -7.1 | -0.355 | -5.9 | -0.295 | -5.9 | -0.295 |
| ZINC01616692 | -6.8 | -0.453 | -5.5 | -0.367 | -5.2 | -0.347 | -5 | -0.333 |
| ZINC01617170 | -6.6 | -0.412 | -5.4 | -0.338 | -5.4 | -0.338 | -5.2 | -0.325 |
| ZINC01617301 | -6.6 | -0.254 | -6.7 | -0.258 | -6.1 | -0.235 | -5.9 | -0.227 |
| ZINC01620955 | -6 | -0.462 | -5.5 | -0.423 | -5.1 | -0.392 | -6 | -0.462 |
| ZINC01621536 | -9.3 | -0.465 | -7.1 | -0.355 | -6.8 | -0.34 | -6.9 | -0.345 |
| ZINC01621931 | -6.2 | -0.413 | -5.4 | -0.36 | -5 | -0.333 | -4.8 | -0.32 |
| ZINC01621981 | -7.7 | -0.35 | -6.9 | -0.314 | -6.3 | -0.286 | -6.2 | -0.282 |
| ZINC01621997 | -7.7 | -0.285 | -7.5 | -0.278 | -7.5 | -0.278 | -6.9 | -0.256 |
| ZINC01622198 | -7.4 | -0.322 | -7 | -0.304 | -6.5 | -0.283 | -6.4 | -0.278 |
| ZINC01622269_1 | -8.6 | -0.391 | -6.6 | -0.3 | -6.5 | -0.295 | -5.7 | -0.259 |
| ZINC01622269 | -8.6 | -0.391 | -6.6 | -0.3 | -6.5 | -0.295 | -5.5 | -0.25 |
| ZINC01625052 | -6.7 | -0.353 | -5.4 | -0.284 | -5.4 | -0.284 | -5.1 | -0.268 |
| ZINC01625094 | -7 | -0.438 | -6 | -0.375 | -5.3 | -0.331 | -5.5 | -0.344 |
| ZINC01625106 | -6.7 | -0.353 | -5.3 | -0.279 | -5.2 | -0.274 | -5 | -0.263 |
| ZINC01625114 | -6.9 | -0.329 | -5.6 | -0.267 | -5.4 | -0.257 | -5 | -0.238 |
| ZINC01625418 | -7.2 | -0.45 | -6.4 | -0.4 | -5.9 | -0.369 | -5.4 | -0.338 |
| ZINC01625444 | -6.9 | -0.46 | -5.7 | -0.38 | -5.5 | -0.367 | -5.7 | -0.38 |
| ZINC01625746 | -8 | -0.258 | -7.5 | -0.242 | -7.3 | -0.235 | -7.4 | -0.239 |
| ZINC01626802 | -7.4 | -0.352 | -7.1 | -0.338 | -6.6 | -0.314 | -6.7 | -0.319 |
| ZINC01626803 | -7.5 | -0.341 | -7.4 | -0.336 | -6.6 | -0.3 | -6.4 | -0.291 |
| ZINC01627101 | -5.1 | -0.464 | -4.5 | -0.409 | -4.3 | -0.391 | -4.5 | -0.409 |
| ZINC01627302 | -6.9 | -0.406 | -6 | -0.353 | -5.6 | -0.329 | -5.6 | -0.329 |
| ZINC01627329 | -8.4 | -0.35 | -7.2 | -0.3 | -6.6 | -0.275 | -6.3 | -0.263 |
| ZINC01627377 | -7.2 | -0.45 | -5.3 | -0.331 | -5.3 | -0.331 | -5.6 | -0.35 |
| ZINC01627439 | -6.6 | -0.471 | -5.5 | -0.393 | -5.4 | -0.386 | -5.4 | -0.386 |
| ZINC01628211 | -6.6 | -0.388 | -6 | -0.353 | -5.7 | -0.335 | -5.4 | -0.318 |
| ZINC01629421 | -6.7 | -0.268 | -7 | -0.28 | -6.2 | -0.248 | -6.1 | -0.244 |
| ZINC01629569 | -7.6 | -0.362 | -7.6 | -0.362 | -6.4 | -0.305 | -6.7 | -0.319 |
| ZINC01629851 | -7.4 | -0.411 | -5.5 | -0.306 | -5.4 | -0.3 | -5.6 | -0.311 |
| ZINC01632739 | -5.1 | -0.464 | -4.5 | -0.409 | -4.3 | -0.391 | -4.3 | -0.391 |
| ZINC01633473 | -7.3 | -0.348 | -6.7 | -0.319 | -6.2 | -0.295 | -5.8 | -0.276 |
| ZINC01633669 | -7.1 | -0.507 | -5.7 | -0.407 | -5.4 | -0.386 | -6.2 | -0.443 |
| ZINC01635676 | -8.2 | -0.273 | -7.7 | -0.257 | -6.3 | -0.21 | -6 | -0.2 |
| ZINC01635813 | -7.9 | -0.416 | -6.4 | -0.337 | -5.9 | -0.311 | -5.8 | -0.305 |
| ZINC01635835 | -8.4 | -0.35 | -6.9 | -0.288 | -7 | -0.292 | -6.6 | -0.275 |
| ZINC01635869 | -7.5 | -0.341 | -7.4 | -0.336 | -6.9 | -0.314 | -6.6 | -0.3 |
| ZINC01637135 | -8.5 | -0.386 | -7 | -0.318 | -6.6 | -0.3 | -6.2 | -0.282 |
| ZINC01637139 | -7.6 | -0.447 | -6.7 | -0.394 | -6.3 | -0.371 | -6 | -0.353 |
| ZINC01637206 | -8 | -0.364 | -6.4 | -0.291 | -6.8 | -0.309 | -6.6 | -0.3 |
| ZINC01637653 | -7.8 | -0.371 | -5.9 | -0.281 | -5.5 | -0.262 | -5.7 | -0.271 |
| ZINC01638088 | -7.8 | -0.371 | -7.4 | -0.352 | -6.6 | -0.314 | -6.6 | -0.314 |
| ZINC01638353 | -7.5 | -0.341 | -6.8 | -0.309 | -6.1 | -0.277 | -6 | -0.273 |
| ZINC01638368 | -6.6 | -0.388 | -5.3 | -0.312 | -5.4 | -0.318 | -5 | -0.294 |
| ZINC01639633 | -8.5 | -0.243 | -7.7 | -0.22 | -8.6 | -0.246 | -8 | -0.229 |
| ZINC01639634 | -9.5 | -0.257 | -7.5 | -0.203 | -7 | -0.189 | -8.3 | -0.224 |
| ZINC01640145 | -6.8 | -0.425 | -5.9 | -0.369 | -5.9 | -0.369 | -5.1 | -0.319 |
| ZINC01640193 | -8.4 | -0.35 | -7.2 | -0.3 | -7.5 | -0.312 | -7 | -0.292 |
| ZINC01640205 | -8.9 | -0.468 | -6.6 | -0.347 | -6.6 | -0.347 | -5.8 | -0.305 |
| ZINC01640219 | -7 | -0.467 | -5.8 | -0.387 | -5.8 | -0.387 | -5.3 | -0.353 |
| ZINC01641160 | -7.6 | -0.422 | -5.9 | -0.328 | -5.5 | -0.306 | -5.9 | -0.328 |
| ZINC01641244 | -6.4 | -0.305 | -7.1 | -0.338 | -6.1 | -0.29 | -6.2 | -0.295 |
| ZINC01641376 | -6.7 | -0.515 | -5.7 | -0.438 | -5.1 | -0.392 | -5.2 | -0.4 |
| ZINC01641466 | -7.1 | -0.473 | -5.9 | -0.393 | -5.5 | -0.367 | -5.4 | -0.36 |
| ZINC01641644 | -6 | -0.462 | -4.9 | -0.377 | -4.7 | -0.362 | -4.6 | -0.354 |
| ZINC01644016 | -6.5 | -0.433 | -5.3 | -0.353 | -5.1 | -0.34 | -5.1 | -0.34 |
| ZINC01644254 | -9.2 | -0.249 | -7.4 | -0.2 | -6.9 | -0.186 | -7.3 | -0.197 |
| ZINC01645454 | -7.4 | -0.274 | -7.7 | -0.285 | -7.1 | -0.263 | -7.1 | -0.263 |
| ZINC01646278 | -7.2 | -0.313 | -7.1 | -0.309 | -6.2 | -0.27 | -6.1 | -0.265 |
| ZINC01646387 | -7.8 | -0.487 | -5.9 | -0.369 | -5.8 | -0.362 | -5.8 | -0.362 |
| ZINC01646690 | -6.2 | -0.517 | -5.3 | -0.442 | -4.7 | -0.392 | -5.4 | -0.45 |
| ZINC01648249 | -7 | -0.389 | -5.5 | -0.306 | -5.3 | -0.294 | -4.9 | -0.272 |
| ZINC01649010 | -7.7 | -0.367 | -6.5 | -0.31 | -6.6 | -0.314 | -6.2 | -0.295 |
| ZINC01649013 | -7.4 | -0.296 | -7.5 | -0.3 | -6.7 | -0.268 | -6 | -0.24 |
| ZINC01649032 | -8.3 | -0.415 | -6.3 | -0.315 | -6 | -0.3 | -5.8 | -0.29 |
| ZINC01652261 | -5.9 | -0.393 | -5.3 | -0.353 | -5.2 | -0.347 | -5.5 | -0.367 |
| ZINC01652969 | -7.3 | -0.384 | -5.6 | -0.295 | -5.5 | -0.289 | -5.1 | -0.268 |
| ZINC01652984 | -8.6 | -0.41 | -6.2 | -0.295 | -6.1 | -0.29 | -6 | -0.286 |
| ZINC01652986 | -7.5 | -0.341 | -6.3 | -0.286 | -6.3 | -0.286 | -6.2 | -0.282 |
| ZINC01653222_1 | -7.6 | -0.507 | -6 | -0.4 | -5.9 | -0.393 | -6.3 | -0.42 |
| ZINC01653222 | -7.8 | -0.52 | -6.1 | -0.407 | -5.9 | -0.393 | -6.2 | -0.413 |
| ZINC01655764 | -7 | -0.5 | -5.4 | -0.386 | -5 | -0.357 | -5 | -0.357 |
| ZINC01655914 | -8 | -0.381 | -6.3 | -0.3 | -6.3 | -0.3 | -5.7 | -0.271 |
| ZINC01656025 | -6.4 | -0.492 | -5.4 | -0.415 | -5 | -0.385 | -5.4 | -0.415 |
| ZINC01656026 | -6.1 | -0.469 | -5.6 | -0.431 | -5.3 | -0.408 | -5.9 | -0.454 |
| ZINC01656027 | -6.2 | -0.477 | -5.4 | -0.415 | -5.4 | -0.415 | -5.6 | -0.431 |
| ZINC01656028 | -6.2 | -0.477 | -5.2 | -0.4 | -5 | -0.385 | -5.6 | -0.431 |
| ZINC01656323 | -7.3 | -0.317 | -6.6 | -0.287 | -6.4 | -0.278 | -6.1 | -0.265 |
| ZINC01657060 | -7.4 | -0.389 | -6.2 | -0.326 | -6.1 | -0.321 | -5.7 | -0.3 |
| ZINC01659930 | -7.1 | -0.374 | -5.6 | -0.295 | -5.5 | -0.289 | -5.5 | -0.289 |
| ZINC01661542 | -6.8 | -0.34 | -7 | -0.35 | -6.4 | -0.32 | -6 | -0.3 |
| ZINC01661545 | -7.6 | -0.345 | -7.1 | -0.323 | -6.2 | -0.282 | -6.2 | -0.282 |
| ZINC01662442 | -8.4 | -0.35 | -7.1 | -0.296 | -6.8 | -0.283 | -6 | -0.25 |
| ZINC01663950 | -7.2 | -0.36 | -7.1 | -0.355 | -6.8 | -0.34 | -6.8 | -0.34 |
| ZINC01663959 | -6.8 | -0.567 | -5.1 | -0.425 | -5.4 | -0.45 | -5.6 | -0.467 |
| ZINC01665042 | -7.5 | -0.3 | -7.4 | -0.296 | -7.4 | -0.296 | -6.7 | -0.268 |
| ZINC01665187 | -7.9 | -0.527 | -6.5 | -0.433 | -6.1 | -0.407 | -6.6 | -0.44 |
| ZINC01665192 | -6.7 | -0.447 | -5.9 | -0.393 | -5.5 | -0.367 | -5.8 | -0.387 |
| ZINC01665194 | -7.2 | -0.48 | -5.8 | -0.387 | -5.4 | -0.36 | -6.1 | -0.407 |
| ZINC01665334 | -6.9 | -0.383 | -6.5 | -0.361 | -6.4 | -0.356 | -5.9 | -0.328 |
| ZINC01665703 | -7.9 | -0.343 | -6.6 | -0.287 | -6.7 | -0.291 | -6 | -0.261 |
| ZINC01665708 | -8 | -0.333 | -6.6 | -0.275 | -6.6 | -0.275 | -6.3 | -0.263 |
| ZINC01665801 | -8.7 | -0.335 | -7.6 | -0.292 | -6.9 | -0.265 | -7.1 | -0.273 |
| ZINC01665928 | -8 | -0.364 | -6.7 | -0.305 | -6.7 | -0.305 | -6.2 | -0.282 |
| ZINC01665940 | -7.7 | -0.35 | -6.5 | -0.295 | -6.3 | -0.286 | -5.9 | -0.268 |
| ZINC01666192 | -5.4 | -0.491 | -4.4 | -0.4 | -4.3 | -0.391 | -4.3 | -0.391 |
| ZINC01666568 | -8 | -0.333 | -7.2 | -0.3 | -6.7 | -0.279 | -6.2 | -0.258 |
| ZINC01666997 | -5.9 | -0.454 | -5.3 | -0.408 | -5.1 | -0.392 | -5.4 | -0.415 |
| ZINC01668114 | -7.1 | -0.355 | -5.8 | -0.29 | -5.8 | -0.29 | -5.3 | -0.265 |
| ZINC01668253 | -7.2 | -0.343 | -7 | -0.333 | -6.1 | -0.29 | -6.5 | -0.31 |
| ZINC01668429 | -6.8 | -0.184 | -7.1 | -0.192 | -7.4 | -0.2 | -7.3 | -0.197 |
| ZINC01668491 | -7 | -0.25 | -6.7 | -0.239 | -6.7 | -0.239 | -6 | -0.214 |
| ZINC01668601 | -8.4 | -0.467 | -7.4 | -0.411 | -6 | -0.333 | -5.7 | -0.317 |
| ZINC01668706 | -6.9 | -0.246 | -6.4 | -0.229 | -6 | -0.214 | -6.1 | -0.218 |
| ZINC01668896_1 | -6.7 | -0.394 | -6.3 | -0.371 | -5.1 | -0.3 | -5.8 | -0.341 |
| ZINC01668896 | -7.2 | -0.424 | -5.7 | -0.335 | -5 | -0.294 | -5.3 | -0.312 |
| ZINC01669376 | -7.3 | -0.456 | -5.7 | -0.356 | -5.8 | -0.362 | -6 | -0.375 |
| ZINC01669572 | -7.3 | -0.27 | -7.1 | -0.263 | -6.6 | -0.244 | -6.5 | -0.241 |
| ZINC01669828 | -9.3 | -0.344 | -6.2 | -0.23 | -5.6 | -0.207 | -4.9 | -0.181 |
| ZINC01670127 | -8 | -0.4 | -6.9 | -0.345 | -6.8 | -0.34 | -6.3 | -0.315 |
| ZINC01670214 | -5.7 | -0.475 | -4.9 | -0.408 | -4.3 | -0.358 | -4.8 | -0.4 |
| ZINC01670291 | -4.9 | -0.408 | -4.2 | -0.35 | -4 | -0.333 | -4.5 | -0.375 |
| ZINC01670341 | -6 | -0.316 | -5.5 | -0.289 | -5.2 | -0.274 | -5.6 | -0.295 |
| ZINC01670393 | -6.4 | -0.376 | -5.5 | -0.324 | -5.7 | -0.335 | -5.6 | -0.329 |
| ZINC01670696 | -7.1 | -0.418 | -5.6 | -0.329 | -5.5 | -0.324 | -5.5 | -0.324 |
| ZINC01670877 | -7.4 | -0.411 | -6.6 | -0.367 | -6.2 | -0.344 | -5.8 | -0.322 |
| ZINC01671311 | -6.6 | -0.44 | -5.1 | -0.34 | -5 | -0.333 | -4.9 | -0.327 |
| ZINC01671321 | -8.9 | -0.405 | -7.2 | -0.327 | -6.5 | -0.295 | -6.8 | -0.309 |
| ZINC01671640 | -6.8 | -0.34 | -7.8 | -0.39 | -6.4 | -0.32 | -6.2 | -0.31 |
| ZINC01671654 | -7.6 | -0.345 | -6.8 | -0.309 | -6.6 | -0.3 | -6.8 | -0.309 |
| ZINC01671866 | -6.7 | -0.353 | -6.9 | -0.363 | -6.9 | -0.363 | -6.6 | -0.347 |
| ZINC01671868 | -8.9 | -0.445 | -7 | -0.35 | -6.6 | -0.33 | -6.2 | -0.31 |
| ZINC01671896 | -8 | -0.381 | -7.4 | -0.352 | -6.8 | -0.324 | -6.5 | -0.31 |
| ZINC01672033 | -6.3 | -0.485 | -5 | -0.385 | -4.6 | -0.354 | -4.9 | -0.377 |
| ZINC01672062 | -6.5 | -0.382 | -5.4 | -0.318 | -5.3 | -0.312 | -5 | -0.294 |
| ZINC01672214 | -6.3 | -0.45 | -5.2 | -0.371 | -4.8 | -0.343 | -4.9 | -0.35 |
| ZINC01672597 | -5.4 | -0.491 | -4.5 | -0.409 | -4.1 | -0.373 | -4.5 | -0.409 |
| ZINC01672599 | -5.8 | -0.483 | -4.7 | -0.392 | -4.4 | -0.367 | -4.7 | -0.392 |
| ZINC01673377 | -7.3 | -0.332 | -6.9 | -0.314 | -5.5 | -0.25 | -5.5 | -0.25 |
| ZINC01673464 | -7.7 | -0.35 | -6.4 | -0.291 | -6.8 | -0.309 | -6.2 | -0.282 |
| ZINC01674390 | -6.2 | -0.517 | -5.5 | -0.458 | -4.9 | -0.408 | -4.5 | -0.375 |
| ZINC01674410 | -8 | -0.333 | -7.2 | -0.3 | -5.8 | -0.242 | -6.2 | -0.258 |
| ZINC01674943 | -5.8 | -0.446 | -4.9 | -0.377 | -4.8 | -0.369 | -4.8 | -0.369 |
| ZINC01675432 | -5.4 | -0.491 | -4.5 | -0.409 | -4.3 | -0.391 | -4 | -0.364 |
| ZINC01675804 | -4.9 | -0.49 | -4.1 | -0.41 | -4 | -0.4 | -3.9 | -0.39 |
| ZINC01675857 | -8.9 | -0.387 | -7 | -0.304 | -6.9 | -0.3 | -6.5 | -0.283 |
| ZINC01675858 | -7.8 | -0.325 | -7.1 | -0.296 | -6.7 | -0.279 | -6.7 | -0.279 |
| ZINC01675990 | -7.8 | -0.433 | -6.7 | -0.372 | -6.1 | -0.339 | -6.4 | -0.356 |
| ZINC01676023 | -7.6 | -0.345 | -6 | -0.273 | -5.4 | -0.245 | -6.2 | -0.282 |
| ZINC01676138 | -7.1 | -0.394 | -6 | -0.333 | -5.6 | -0.311 | -5.8 | -0.322 |
| ZINC01676139 | -6.9 | -0.383 | -6.3 | -0.35 | -6.1 | -0.339 | -5.8 | -0.322 |
| ZINC01676213 | -7.4 | -0.493 | -5.8 | -0.387 | -5.3 | -0.353 | -5.6 | -0.373 |
| ZINC01676257 | -6.3 | -0.485 | -5.2 | -0.4 | -4.9 | -0.377 | -5.4 | -0.415 |
| ZINC01676311 | -6.3 | -0.225 | -6.5 | -0.232 | -6.2 | -0.221 | -6.1 | -0.218 |
| ZINC01677276 | -6.7 | -0.447 | -5.2 | -0.347 | -5.1 | -0.34 | -5 | -0.333 |
| ZINC01677554 | -8.3 | -0.437 | -6.5 | -0.342 | -6.3 | -0.332 | -6.3 | -0.332 |
| ZINC01677761 | -7.2 | -0.277 | -7 | -0.269 | -6.9 | -0.265 | -6 | -0.231 |
| ZINC01677892 | -8.2 | -0.357 | -6.6 | -0.287 | -7 | -0.304 | -6.5 | -0.283 |
| ZINC01679310 | -7.4 | -0.463 | -5.7 | -0.356 | -5.7 | -0.356 | -5.7 | -0.356 |
| ZINC01679777 | -6.4 | -0.492 | -5.5 | -0.423 | -5.1 | -0.392 | -5.6 | -0.431 |
| ZINC01680284 | -7.3 | -0.429 | -5.8 | -0.341 | -5.5 | -0.324 | -5.4 | -0.318 |
| ZINC01680565 | -6.3 | -0.485 | -5.1 | -0.392 | -5.1 | -0.392 | -4.8 | -0.369 |
| ZINC01681130 | -7.9 | -0.494 | -6.1 | -0.381 | -6.2 | -0.388 | -5.9 | -0.369 |
| ZINC01681557 | -7 | -0.412 | -5.9 | -0.347 | -6 | -0.353 | -5.8 | -0.341 |
| ZINC01682029 | -6.4 | -0.376 | -5.8 | -0.341 | -5.2 | -0.306 | -5.3 | -0.312 |
| ZINC01682117 | -6.6 | -0.471 | -5.5 | -0.393 | -5.3 | -0.379 | -5.7 | -0.407 |
| ZINC01682520 | -7.8 | -0.459 | -6.5 | -0.382 | -6.1 | -0.359 | -6.1 | -0.359 |
| ZINC01682534 | -8.1 | -0.386 | -6.5 | -0.31 | -6 | -0.286 | -6 | -0.286 |
| ZINC01682798 | -6.4 | -0.291 | -7 | -0.318 | -6.1 | -0.277 | -5.9 | -0.268 |
| ZINC01683295 | -8.4 | -0.382 | -6.7 | -0.305 | -6.7 | -0.305 | -6.4 | -0.291 |
| ZINC01683317 | -7.2 | -0.514 | -6.1 | -0.436 | -5.4 | -0.386 | -6.4 | -0.457 |
| ZINC01683345 | -7.4 | -0.37 | -6.4 | -0.32 | -6.2 | -0.31 | -5.5 | -0.275 |
| ZINC01683531 | -7.7 | -0.453 | -6.4 | -0.376 | -6 | -0.353 | -6 | -0.353 |
| ZINC01683553 | -7.3 | -0.348 | -6.3 | -0.3 | -6.2 | -0.295 | -6 | -0.286 |
| ZINC01683648 | -8.3 | -0.437 | -6.2 | -0.326 | -5.9 | -0.311 | -5.7 | -0.3 |
| ZINC01685254 | -7.7 | -0.367 | -6.8 | -0.324 | -6.1 | -0.29 | -6.4 | -0.305 |
| ZINC01685697 | -6.9 | -0.3 | -6.4 | -0.278 | -6 | -0.261 | -5.9 | -0.257 |
| ZINC01685876 | -7.6 | -0.4 | -6 | -0.316 | -5.7 | -0.3 | -5.3 | -0.279 |
| ZINC01685881 | -5.5 | -0.458 | -5 | -0.417 | -4.4 | -0.367 | -5.5 | -0.458 |
| ZINC01685966 | -5.9 | -0.492 | -5 | -0.417 | -4.5 | -0.375 | -4.9 | -0.408 |
| ZINC01686251 | -6.5 | -0.433 | -5.7 | -0.38 | -5 | -0.333 | -5 | -0.333 |
| ZINC01686363 | -6.9 | -0.493 | -6.2 | -0.443 | -5.6 | -0.4 | -5.9 | -0.421 |
| ZINC01686467 | -8.2 | -0.357 | -6.6 | -0.287 | -5.9 | -0.257 | -5.6 | -0.243 |
| ZINC01686683 | -6.3 | -0.45 | -5.7 | -0.407 | -4.9 | -0.35 | -5.1 | -0.364 |
| ZINC01686969 | -9.1 | -0.396 | -7.2 | -0.313 | -6.6 | -0.287 | -6.5 | -0.283 |
| ZINC01687247 | -8.2 | -0.265 | -8.8 | -0.284 | -6.8 | -0.219 | -7.2 | -0.232 |
| ZINC01687845 | -6.8 | -0.486 | -5.7 | -0.407 | -5.2 | -0.371 | -5.1 | -0.364 |
| ZINC01687910 | -6.2 | -0.388 | -5.5 | -0.344 | -4.7 | -0.294 | -4.7 | -0.294 |
| ZINC01688151 | -6.1 | -0.218 | -6.2 | -0.221 | -6.3 | -0.225 | -6.2 | -0.221 |
| ZINC01688347 | -6.8 | -0.358 | -5.8 | -0.305 | -5.1 | -0.268 | -5 | -0.263 |
| ZINC01688628 | -8.1 | -0.476 | -6.5 | -0.382 | -5.9 | -0.347 | -5.6 | -0.329 |
| ZINC01688646 | -6.9 | -0.46 | -5.7 | -0.38 | -5.2 | -0.347 | -5.6 | -0.373 |
| ZINC01688662_1 | -5.2 | -0.578 | -4.3 | -0.478 | -4 | -0.444 | -4.6 | -0.511 |
| ZINC01688662 | -5.2 | -0.578 | -4.4 | -0.489 | -4.2 | -0.467 | -4.6 | -0.511 |
| ZINC01688752 | -6.7 | -0.479 | -5.4 | -0.386 | -4.9 | -0.35 | -6 | -0.429 |
| ZINC01689447 | -6.7 | -0.419 | -5.3 | -0.331 | -5.2 | -0.325 | -5 | -0.312 |
| ZINC01689932 | -7.2 | -0.379 | -6.3 | -0.332 | -6 | -0.316 | -5.5 | -0.289 |
| ZINC01690169 | -6 | -0.462 | -5 | -0.385 | -4.8 | -0.369 | -4.6 | -0.354 |
| ZINC01690171 | -5.4 | -0.45 | -4.7 | -0.392 | -4.6 | -0.383 | -4.4 | -0.367 |
| ZINC01690194 | -8 | -0.5 | -6.3 | -0.394 | -6.2 | -0.388 | -5.7 | -0.356 |
| ZINC01690208 | -7.5 | -0.341 | -6.6 | -0.3 | -6.2 | -0.282 | -5.9 | -0.268 |
| ZINC01690699 | -8.5 | -0.202 | -9.1 | -0.217 | -9.7 | -0.231 | -8.9 | -0.212 |
| ZINC01690722 | -7.2 | -0.277 | -6.8 | -0.262 | -6.3 | -0.242 | -6.1 | -0.235 |
| ZINC01691075 | -6.4 | -0.492 | -5 | -0.385 | -4.6 | -0.354 | -4.6 | -0.354 |
| ZINC01691548 | -6.3 | -0.45 | -5.3 | -0.379 | -5.1 | -0.364 | -4.7 | -0.336 |
| ZINC01691551 | -6.2 | -0.443 | -5.4 | -0.386 | -5.2 | -0.371 | -4.9 | -0.35 |
| ZINC01691651 | -8.3 | -0.296 | -6.6 | -0.236 | -7.6 | -0.271 | -7.5 | -0.268 |
| ZINC01691782 | -6.5 | -0.283 | -7.3 | -0.317 | -6.2 | -0.27 | -6.2 | -0.27 |
| ZINC01691943 | -6.4 | -0.237 | -8 | -0.296 | -6.5 | -0.241 | -6.1 | -0.226 |
| ZINC01691989 | -8.4 | -0.442 | -6.9 | -0.363 | -6.5 | -0.342 | -5.6 | -0.295 |
| ZINC01692470 | -6.7 | -0.394 | -5.5 | -0.324 | -5.5 | -0.324 | -5.6 | -0.329 |
| ZINC01693279 | -8 | -0.333 | -6.6 | -0.275 | -6.6 | -0.275 | -6 | -0.25 |
| ZINC01694053 | -9.3 | -0.266 | -7.4 | -0.211 | -7.6 | -0.217 | -7.8 | -0.223 |
| ZINC01694371 | -5.3 | -0.482 | -4.3 | -0.391 | -4.1 | -0.373 | -4.1 | -0.373 |
| ZINC01695372 | -8.4 | -0.42 | -6.3 | -0.315 | -6.9 | -0.345 | -5.9 | -0.295 |
| ZINC01695559 | -6.9 | -0.329 | -6.9 | -0.329 | -6.4 | -0.305 | -6.4 | -0.305 |
| ZINC01696555 | -6.7 | -0.515 | -5.7 | -0.438 | -5.6 | -0.431 | -5.5 | -0.423 |
| ZINC01697077 | -7 | -0.412 | -6 | -0.353 | -5.8 | -0.341 | -5.7 | -0.335 |
| ZINC01697110 | -8 | -0.471 | -6.2 | -0.365 | -5.9 | -0.347 | -5.8 | -0.341 |
| ZINC01697295 | -7.5 | -0.417 | -6 | -0.333 | -6.1 | -0.339 | -6.1 | -0.339 |
| ZINC01697912 | -6.4 | -0.457 | -5.2 | -0.371 | -5.1 | -0.364 | -5.4 | -0.386 |
| ZINC01699287 | -9.4 | -0.261 | -7.3 | -0.203 | -7.4 | -0.206 | -7.6 | -0.211 |
| ZINC01699937 | -7.4 | -0.435 | -5.6 | -0.329 | -5.7 | -0.335 | -5.4 | -0.318 |
| ZINC01700219 | -7.2 | -0.4 | -6.6 | -0.367 | -6.2 | -0.344 | -6.2 | -0.344 |
| ZINC01700953 | -8.5 | -0.447 | -7 | -0.368 | -6.1 | -0.321 | -6.5 | -0.342 |
| ZINC01701112 | -7.7 | -0.513 | -6.3 | -0.42 | -5.4 | -0.36 | -6.1 | -0.407 |
| ZINC01701460 | -7.5 | -0.3 | -7.8 | -0.312 | -7.3 | -0.292 | -6.7 | -0.268 |
| ZINC01701513 | -6.2 | -0.443 | -5.1 | -0.364 | -4.9 | -0.35 | -5.5 | -0.393 |
| ZINC01702739 | -8.5 | -0.354 | -7 | -0.292 | -6.9 | -0.288 | -5.9 | -0.246 |
| ZINC01702835 | -7.3 | -0.456 | -5.6 | -0.35 | -5.2 | -0.325 | -5.8 | -0.362 |
| ZINC01703050 | -7.6 | -0.447 | -6.7 | -0.394 | -5.6 | -0.329 | -5.4 | -0.318 |
| ZINC01703105 | -7.5 | -0.536 | -5.6 | -0.4 | -5.3 | -0.379 | -5.3 | -0.379 |
| ZINC01703109 | -5.8 | -0.322 | -5.3 | -0.294 | -5.2 | -0.289 | -4.8 | -0.267 |
| ZINC01703286 | -7.4 | -0.493 | -6.2 | -0.413 | -5.7 | -0.38 | -6 | -0.4 |
| ZINC01705443 | -7.9 | -0.272 | -8.1 | -0.279 | -6.5 | -0.224 | -6.5 | -0.224 |
| ZINC01705919 | -8 | -0.286 | -7.7 | -0.275 | -7.7 | -0.275 | -7.3 | -0.261 |
| ZINC01705925 | -8.7 | -0.322 | -8.1 | -0.3 | -7 | -0.259 | -7 | -0.259 |
| ZINC01706083 | -7.3 | -0.365 | -6.1 | -0.305 | -5.9 | -0.295 | -5.8 | -0.29 |
| ZINC01706126 | -7.9 | -0.359 | -7.9 | -0.359 | -6.3 | -0.286 | -6.3 | -0.286 |
| ZINC01706223 | -7.3 | -0.384 | -7.9 | -0.416 | -6.6 | -0.347 | -6.3 | -0.332 |
| ZINC01706234 | -6.8 | -0.486 | -5.7 | -0.407 | -5.3 | -0.379 | -5.6 | -0.4 |
| ZINC01706740 | -5.7 | -0.475 | -5 | -0.417 | -4.9 | -0.408 | -5.1 | -0.425 |
| ZINC01706914 | -7.8 | -0.433 | -5.9 | -0.328 | -5.5 | -0.306 | -5.4 | -0.3 |
| ZINC01706915 | -6.6 | -0.367 | -5.8 | -0.322 | -5.8 | -0.322 | -5.3 | -0.294 |
| ZINC01707109 | -7.9 | -0.316 | -7.2 | -0.288 | -6.3 | -0.252 | -6.5 | -0.26 |
| ZINC01707130 | -10 | -0.455 | -7.2 | -0.327 | -7 | -0.318 | -7.3 | -0.332 |
| ZINC01707215 | -7 | -0.28 | -6.9 | -0.276 | -6.4 | -0.256 | -5.9 | -0.236 |
| ZINC01707482 | -6.9 | -0.363 | -7.1 | -0.374 | -6.3 | -0.332 | -6 | -0.316 |
| ZINC01707724 | -7.4 | -0.493 | -5.4 | -0.36 | -5.2 | -0.347 | -6.2 | -0.413 |
| ZINC01708688 | -6.3 | -0.485 | -5.4 | -0.415 | -4.9 | -0.377 | -5.5 | -0.423 |
| ZINC01709784 | -6.8 | -0.262 | -6.4 | -0.246 | -6.4 | -0.246 | -5.6 | -0.215 |
| ZINC01709785 | -7 | -0.318 | -6.9 | -0.314 | -6.2 | -0.282 | -6.2 | -0.282 |
| ZINC01710091 | -8 | -0.348 | -5.6 | -0.243 | -5.7 | -0.248 | -6.1 | -0.265 |
| ZINC01710099 | -7.8 | -0.411 | -5.4 | -0.284 | -5.9 | -0.311 | -4.9 | -0.258 |
| ZINC01710435 | -7.1 | -0.355 | -5.6 | -0.28 | -5.7 | -0.285 | -5 | -0.25 |
| ZINC01710961 | -7.6 | -0.422 | -6.2 | -0.344 | -5.9 | -0.328 | -5.5 | -0.306 |
| ZINC01711028 | -7.4 | -0.285 | -7.9 | -0.304 | -7.4 | -0.285 | -6.8 | -0.262 |
| ZINC01711796 | -7.6 | -0.447 | -5.9 | -0.347 | -5.6 | -0.329 | -5.9 | -0.347 |
| ZINC01712457 | -6.5 | -0.361 | -5.6 | -0.311 | -5.2 | -0.289 | -4.9 | -0.272 |
| ZINC01712458 | -6.2 | -0.477 | -5.3 | -0.408 | -5.2 | -0.4 | -5.7 | -0.438 |
| ZINC01712637 | -6.1 | -0.469 | -5.4 | -0.415 | -5 | -0.385 | -5 | -0.385 |
| ZINC01712658 | -7.5 | -0.375 | -6.8 | -0.34 | -6.8 | -0.34 | -6 | -0.3 |
| ZINC01712744 | -7 | -0.438 | -6 | -0.375 | -5.7 | -0.356 | -5.7 | -0.356 |
| ZINC01712829 | -7.1 | -0.374 | -5.7 | -0.3 | -5.2 | -0.274 | -5 | -0.263 |
| ZINC01712830 | -7.2 | -0.379 | -5.6 | -0.295 | -5.4 | -0.284 | -5.2 | -0.274 |
| ZINC01712866 | -8 | -0.348 | -6.6 | -0.287 | -6.5 | -0.283 | -5.9 | -0.257 |
| ZINC01713460 | -6.6 | -0.44 | -5.4 | -0.36 | -5.1 | -0.34 | -5.4 | -0.36 |
| ZINC01714961 | -7.3 | -0.384 | -5.7 | -0.3 | -5.6 | -0.295 | -5.4 | -0.284 |
| ZINC01714962 | -7.9 | -0.359 | -6.3 | -0.286 | -5.7 | -0.259 | -5.2 | -0.236 |
| ZINC01716722 | -6.1 | -0.359 | -5.7 | -0.335 | -5.1 | -0.3 | -5.1 | -0.3 |
| ZINC01717579 | -8.2 | -0.39 | -6.5 | -0.31 | -6.6 | -0.314 | -6.3 | -0.3 |
| ZINC01718481 | -6.9 | -0.3 | -6.2 | -0.27 | -5.8 | -0.252 | -6 | -0.261 |
| ZINC01718482 | -7 | -0.318 | -6.5 | -0.295 | -6.2 | -0.282 | -6.2 | -0.282 |
| ZINC01718485 | -8.6 | -0.331 | -6.9 | -0.265 | -6.7 | -0.258 | -6.4 | -0.246 |
| ZINC01718486 | -8 | -0.308 | -7.2 | -0.277 | -6.9 | -0.265 | -6.7 | -0.258 |
| ZINC01718899 | -5.6 | -0.509 | -4.4 | -0.4 | -4.5 | -0.409 | -5 | -0.455 |
| ZINC01718905 | -7.8 | -0.371 | -6.7 | -0.319 | -6.9 | -0.329 | -6.2 | -0.295 |
| ZINC01719297 | -6.7 | -0.558 | -5.5 | -0.458 | -5.2 | -0.433 | -5.3 | -0.442 |
| ZINC01719567 | -8.1 | -0.245 | -7.7 | -0.233 | -7.8 | -0.236 | -6.9 | -0.209 |
| ZINC01719937 | -6.9 | -0.345 | -5 | -0.25 | -5.1 | -0.255 | -4.7 | -0.235 |
| ZINC01719953 | -7.9 | -0.465 | -5.6 | -0.329 | -5.6 | -0.329 | -5.5 | -0.324 |
| ZINC01721304 | -6.2 | -0.517 | -4.8 | -0.4 | -4.6 | -0.383 | -5.1 | -0.425 |
| ZINC01722140 | -7.1 | -0.507 | -5.9 | -0.421 | -5.7 | -0.407 | -6.3 | -0.45 |
| ZINC01722585 | -8.2 | -0.273 | -7.4 | -0.247 | -6.5 | -0.217 | -6 | -0.2 |
| ZINC01724003 | -7.6 | -0.422 | -5.7 | -0.317 | -5.7 | -0.317 | -5.6 | -0.311 |
| ZINC01724376 | -7 | -0.412 | -6.2 | -0.365 | -6 | -0.353 | -6.1 | -0.359 |
| ZINC01724593 | -8.8 | -0.419 | -7 | -0.333 | -7.2 | -0.343 | -6.4 | -0.305 |
| ZINC01724660 | -8.5 | -0.472 | -6.4 | -0.356 | -6.3 | -0.35 | -5.9 | -0.328 |
| ZINC01725942 | -8.1 | -0.405 | -6.9 | -0.345 | -6.3 | -0.315 | -6.8 | -0.34 |
| ZINC01726729 | -7.1 | -0.374 | -5.9 | -0.311 | -5.9 | -0.311 | -5.8 | -0.305 |
| ZINC01726736 | -7.6 | -0.345 | -6.4 | -0.291 | -6.3 | -0.286 | -6.2 | -0.282 |
| ZINC01726776 | -8.4 | -0.29 | -7.9 | -0.272 | -7.4 | -0.255 | -7.9 | -0.272 |
| ZINC01728118 | -7.6 | -0.475 | -5.7 | -0.356 | -5.2 | -0.325 | -6.4 | -0.4 |
| ZINC01728119 | -6.3 | -0.394 | -5.5 | -0.344 | -4.9 | -0.306 | -5.4 | -0.338 |
| ZINC01728120 | -6.6 | -0.412 | -5.6 | -0.35 | -5 | -0.312 | -5.3 | -0.331 |
| ZINC01728503 | -8.1 | -0.405 | -6.1 | -0.305 | -6.1 | -0.305 | -6.1 | -0.305 |
| ZINC01728821 | -8 | -0.333 | -6.5 | -0.271 | -6.5 | -0.271 | -6.5 | -0.271 |
| ZINC01728888 | -8.1 | -0.352 | -7.1 | -0.309 | -6.5 | -0.283 | -6.5 | -0.283 |
| ZINC01729467 | -6.6 | -0.314 | -6.3 | -0.3 | -5.8 | -0.276 | -5.7 | -0.271 |
| ZINC01729523 | -7.6 | -0.304 | -7.3 | -0.292 | -7.1 | -0.284 | -6.7 | -0.268 |
| ZINC01729524 | -7.8 | -0.312 | -7.1 | -0.284 | -6.9 | -0.276 | -6.8 | -0.272 |
| ZINC01729525 | -7.6 | -0.304 | -7.1 | -0.284 | -6.7 | -0.268 | -6.5 | -0.26 |
| ZINC01729526 | -7.6 | -0.304 | -7 | -0.28 | -6.8 | -0.272 | -6.8 | -0.272 |
| ZINC01729549 | -7.3 | -0.429 | -5.4 | -0.318 | -5.7 | -0.335 | -5.7 | -0.335 |
| ZINC01729578 | -8.4 | -0.365 | -6.7 | -0.291 | -6.6 | -0.287 | -6.1 | -0.265 |
| ZINC01729618 | -7.4 | -0.389 | -5.9 | -0.311 | -5.4 | -0.284 | -5 | -0.263 |
| ZINC01730238 | -7.2 | -0.379 | -5.8 | -0.305 | -5.7 | -0.3 | -5.7 | -0.3 |
| ZINC01731085 | -6.2 | -0.31 | -6.4 | -0.32 | -5.6 | -0.28 | -5.6 | -0.28 |
| ZINC01732051 | -6.3 | -0.485 | -6 | -0.462 | -5 | -0.385 | -6.3 | -0.485 |
| ZINC01732509 | -5.8 | -0.446 | -5.1 | -0.392 | -4.9 | -0.377 | -4.5 | -0.346 |
| ZINC01733235 | -7.7 | -0.275 | -6.8 | -0.243 | -6.6 | -0.236 | -6 | -0.214 |
| ZINC01733511 | -5.3 | -0.408 | -4.4 | -0.338 | -4 | -0.308 | -4.2 | -0.323 |
| ZINC01734080 | -7.4 | -0.435 | -6.2 | -0.365 | -5.9 | -0.347 | -5.4 | -0.318 |
| ZINC01734092 | -8.8 | -0.314 | -7.7 | -0.275 | -7.1 | -0.254 | -6.8 | -0.243 |
| ZINC01734413 | -7.5 | -0.326 | -7.5 | -0.326 | -7 | -0.304 | -7 | -0.304 |
| ZINC01734846 | -6.1 | -0.469 | -4.8 | -0.369 | -4.5 | -0.346 | -4.6 | -0.354 |
| ZINC01735469 | -8.1 | -0.261 | -7.6 | -0.245 | -7 | -0.226 | -6.9 | -0.223 |
| ZINC01735767 | -7.9 | -0.316 | -7.7 | -0.308 | -7 | -0.28 | -6.9 | -0.276 |
| ZINC01735773 | -9.1 | -0.379 | -6.5 | -0.271 | -6.9 | -0.288 | -6.7 | -0.279 |
| ZINC01736227 | -8.9 | -0.297 | -9.1 | -0.303 | -8.2 | -0.273 | -8 | -0.267 |
| ZINC01736228 | -9.1 | -0.303 | -9 | -0.3 | -8.5 | -0.283 | -7.9 | -0.263 |
| ZINC01737161 | -7.9 | -0.395 | -6.7 | -0.335 | -6.7 | -0.335 | -6.4 | -0.32 |
| ZINC01737351 | -6.4 | -0.427 | -5.1 | -0.34 | -4.9 | -0.327 | -4.9 | -0.327 |
| ZINC01738764 | -8.3 | -0.277 | -7.9 | -0.263 | -7.5 | -0.25 | -7.4 | -0.247 |
| ZINC01738918 | -8.6 | -0.374 | -7.1 | -0.309 | -7.1 | -0.309 | -6.7 | -0.291 |
| ZINC01739349 | -8 | -0.381 | -6.4 | -0.305 | -6.1 | -0.29 | -5.7 | -0.271 |
| ZINC01739733 | -7.6 | -0.292 | -7 | -0.269 | -6.4 | -0.246 | -6.5 | -0.25 |
| ZINC01740602 | -7 | -0.438 | -5.8 | -0.362 | -5.5 | -0.344 | -5.3 | -0.331 |
| ZINC01741812 | -7.8 | -0.325 | -6.2 | -0.258 | -6 | -0.25 | -5.8 | -0.242 |
| ZINC01742542 | -5.9 | -0.492 | -4.8 | -0.4 | -4.7 | -0.392 | -5.2 | -0.433 |
| ZINC01744749 | -7.7 | -0.335 | -7 | -0.304 | -6.5 | -0.283 | -6.5 | -0.283 |
| ZINC01744953 | -7.4 | -0.493 | -6.6 | -0.44 | -5.9 | -0.393 | -6 | -0.4 |
| ZINC01745539 | -8 | -0.421 | -6.3 | -0.332 | -6.4 | -0.337 | -5.7 | -0.3 |
| ZINC01747258 | -6.7 | -0.394 | -5.5 | -0.324 | -4.8 | -0.282 | -5.1 | -0.3 |
| ZINC01747274 | -6.7 | -0.353 | -5.6 | -0.295 | -5.1 | -0.268 | -5 | -0.263 |
| ZINC01747299 | -8.1 | -0.368 | -6.7 | -0.305 | -6.3 | -0.286 | -6 | -0.273 |
| ZINC01747332 | -7.1 | -0.273 | -6.5 | -0.25 | -5.8 | -0.223 | -5.7 | -0.219 |
| ZINC01748006 | -7.3 | -0.456 | -5.8 | -0.362 | -5.2 | -0.325 | -5.3 | -0.331 |
| ZINC01748056 | -6.6 | -0.412 | -5.7 | -0.356 | -4.9 | -0.306 | -4.9 | -0.306 |
| ZINC01748097 | -8.1 | -0.337 | -6.7 | -0.279 | -6.5 | -0.271 | -6.4 | -0.267 |
| ZINC01748594 | -7.2 | -0.4 | -5.6 | -0.311 | -5.5 | -0.306 | -5.5 | -0.306 |
| ZINC01748908 | -7.3 | -0.209 | -7.7 | -0.22 | -7.3 | -0.209 | -7 | -0.2 |
| ZINC01749571 | -7.5 | -0.312 | -6.2 | -0.258 | -5.4 | -0.225 | -5.6 | -0.233 |
| ZINC01751425 | -8.6 | -0.41 | -6.9 | -0.329 | -6.7 | -0.319 | -6.4 | -0.305 |
| ZINC01751437 | -7.8 | -0.433 | -6.7 | -0.372 | -6.7 | -0.372 | -6.2 | -0.344 |
| ZINC01752308 | -5.5 | -0.393 | -4.8 | -0.343 | -4.1 | -0.293 | -4.2 | -0.3 |
| ZINC01752784 | -9.8 | -0.35 | -7.4 | -0.264 | -7.7 | -0.275 | -7.1 | -0.254 |
| ZINC01752973 | -7.7 | -0.428 | -6.7 | -0.372 | -6.7 | -0.372 | -5.9 | -0.328 |
| ZINC01753336 | -7.9 | -0.376 | -7.3 | -0.348 | -7.1 | -0.338 | -6.6 | -0.314 |
| ZINC01753723 | -7.6 | -0.362 | -5.8 | -0.276 | -6.2 | -0.295 | -5.9 | -0.281 |
| ZINC01753725 | -8 | -0.348 | -6.2 | -0.27 | -6.3 | -0.274 | -6.1 | -0.265 |
| ZINC01753735 | -7.1 | -0.394 | -5.4 | -0.3 | -5.8 | -0.322 | -5.9 | -0.328 |
| ZINC01753761 | -7.6 | -0.292 | -6.7 | -0.258 | -6.1 | -0.235 | -6.7 | -0.258 |
| ZINC01754899 | -7.3 | -0.487 | -6.5 | -0.433 | -5.5 | -0.367 | -5.6 | -0.373 |
| ZINC01755448 | -7.3 | -0.243 | -8.3 | -0.277 | -7.8 | -0.26 | -7.5 | -0.25 |
| ZINC01755627 | -7.7 | -0.385 | -6.2 | -0.31 | -5.8 | -0.29 | -5.7 | -0.285 |
| ZINC01757986 | -8.7 | -0.435 | -6.6 | -0.33 | -6.4 | -0.32 | -5.9 | -0.295 |
| ZINC01761347 | -6.7 | -0.479 | -5.3 | -0.379 | -5.7 | -0.407 | -5.5 | -0.393 |
| ZINC01761361 | -7.6 | -0.447 | -6.4 | -0.376 | -6.4 | -0.376 | -5.9 | -0.347 |
| ZINC01761642 | -6.6 | -0.471 | -5.6 | -0.4 | -5.5 | -0.393 | -6 | -0.429 |
| ZINC01761875 | -6.6 | -0.367 | -7.4 | -0.411 | -6.3 | -0.35 | -6.1 | -0.339 |
| ZINC01761888 | -6.3 | -0.371 | -6.5 | -0.382 | -5.6 | -0.329 | -5.5 | -0.324 |
| ZINC01764014 | -7.6 | -0.345 | -6.2 | -0.282 | -6.2 | -0.282 | -5.8 | -0.264 |
| ZINC01820901 | -5.1 | -0.51 | -4.5 | -0.45 | -4.1 | -0.41 | -4.6 | -0.46 |
| ZINC01834023 | -7.4 | -0.264 | -7.7 | -0.275 | -6.6 | -0.236 | -7 | -0.25 |
| ZINC01843029_1 | -5.8 | -0.527 | -5.2 | -0.473 | -5.6 | -0.509 | -5.6 | -0.509 |
| ZINC01843029 | -5.8 | -0.527 | -5.3 | -0.482 | -5.6 | -0.509 | -5.6 | -0.509 |
| ZINC01843030_1 | -6.4 | -0.582 | -5.6 | -0.509 | -5.4 | -0.491 | -5.5 | -0.5 |
| ZINC01843030 | -6 | -0.545 | -5.5 | -0.5 | -5.4 | -0.491 | -5.4 | -0.491 |
| ZINC01843071 | -7.5 | -0.536 | -5.7 | -0.407 | -5.3 | -0.379 | -5.1 | -0.364 |
| ZINC01845586 | -8.2 | -0.39 | -6.9 | -0.329 | -6.7 | -0.319 | -6.3 | -0.3 |
| ZINC01845598 | -7.3 | -0.384 | -6 | -0.316 | -5.8 | -0.305 | -5.5 | -0.289 |
| ZINC01846592 | -7 | -0.368 | -7.1 | -0.374 | -6.1 | -0.321 | -6.2 | -0.326 |
| ZINC01848197 | -6.3 | -0.525 | -5.2 | -0.433 | -4.8 | -0.4 | -5.6 | -0.467 |
| ZINC01848198 | -6.4 | -0.492 | -5.2 | -0.4 | -5 | -0.385 | -6.1 | -0.469 |
| ZINC01855333 | -7.9 | -0.293 | -8.4 | -0.311 | -8.4 | -0.311 | -8.1 | -0.3 |
| ZINC01856534 | -7.1 | -0.284 | -6.4 | -0.256 | -6.3 | -0.252 | -5.5 | -0.22 |
| ZINC01865671 | -7.2 | -0.36 | -5.5 | -0.275 | -5.6 | -0.28 | -5.5 | -0.275 |
| ZINC01867048 | -6.3 | -0.45 | -5.3 | -0.379 | -5 | -0.357 | -5.3 | -0.379 |
| ZINC01868209 | -8.4 | -0.35 | -6.9 | -0.288 | -6.5 | -0.271 | -6.5 | -0.271 |
| ZINC01871103 | -8.2 | -0.39 | -6.7 | -0.319 | -6.5 | -0.31 | -6.2 | -0.295 |
| ZINC01871223 | -6.7 | -0.319 | -7.4 | -0.352 | -6.3 | -0.3 | -6.1 | -0.29 |
| ZINC01871349 | -6.4 | -0.457 | -5.1 | -0.364 | -4.8 | -0.343 | -4.4 | -0.314 |
| ZINC01872881 | -7.3 | -0.521 | -5.9 | -0.421 | -5.2 | -0.371 | -5.3 | -0.379 |
| ZINC01873142 | -7 | -0.538 | -5.4 | -0.415 | -5.2 | -0.4 | -5.2 | -0.4 |
| ZINC02011568 | -7 | -0.304 | -7.2 | -0.313 | -6 | -0.261 | -6 | -0.261 |
| ZINC02015922 | -8 | -0.333 | -6.9 | -0.288 | -6.2 | -0.258 | -6.3 | -0.263 |
| ZINC02018030_1 | -7.1 | -0.444 | -6.2 | -0.388 | -5.7 | -0.356 | -5.8 | -0.362 |
| ZINC02018030 | -7.1 | -0.444 | -6.1 | -0.381 | -5.7 | -0.356 | -5.8 | -0.362 |
| ZINC02029995 | -8 | -0.421 | -5.9 | -0.311 | -6.4 | -0.337 | -5.5 | -0.289 |
| ZINC02030136 | -6.6 | -0.347 | -6.3 | -0.332 | -6.2 | -0.326 | -6.3 | -0.332 |
| ZINC02033748 | -7.5 | -0.375 | -5.8 | -0.29 | -5.7 | -0.285 | -5.5 | -0.275 |
| ZINC02034999 | -7.8 | -0.223 | -7.6 | -0.217 | -6.9 | -0.197 | -7.2 | -0.206 |
| ZINC02035165 | -7.4 | -0.322 | -6.1 | -0.265 | -6 | -0.261 | -5.8 | -0.252 |
| ZINC02036484 | -7.7 | -0.367 | -6.7 | -0.319 | -6.5 | -0.31 | -5.5 | -0.262 |
| ZINC02036556 | -6.8 | -0.453 | -5.2 | -0.347 | -4.8 | -0.32 | -5.4 | -0.36 |
| ZINC02038558 | -7.3 | -0.487 | -5.9 | -0.393 | -5.3 | -0.353 | -5.8 | -0.387 |
| ZINC02042220 | -7.4 | -0.435 | -6.3 | -0.371 | -5.9 | -0.347 | -5.2 | -0.306 |
| ZINC02042648 | -7.5 | -0.312 | -6.8 | -0.283 | -6.1 | -0.254 | -5.7 | -0.238 |
| ZINC02042811 | -6.7 | -0.394 | -6.3 | -0.371 | -6.1 | -0.359 | -6 | -0.353 |
| ZINC02042840 | -8.2 | -0.39 | -6.6 | -0.314 | -6.5 | -0.31 | -6.1 | -0.29 |
| ZINC02042931 | -7.3 | -0.429 | -5.6 | -0.329 | -5.5 | -0.324 | -5.5 | -0.324 |
| ZINC02043260 | -7.4 | -0.411 | -6.4 | -0.356 | -6.1 | -0.339 | -6.2 | -0.344 |
| ZINC02045340 | -6.5 | -0.433 | -5.4 | -0.36 | -4.9 | -0.327 | -4.9 | -0.327 |
| ZINC02046452 | -9.5 | -0.452 | -7.8 | -0.371 | -6.9 | -0.329 | -6.8 | -0.324 |
| ZINC02046726 | -7.2 | -0.313 | -7.3 | -0.317 | -6.1 | -0.265 | -5.7 | -0.248 |
| ZINC02048098 | -5.3 | -0.53 | -4.7 | -0.47 | -4.1 | -0.41 | -4.3 | -0.43 |
| ZINC02048214 | -7.3 | -0.456 | -6 | -0.375 | -5.7 | -0.356 | -5.5 | -0.344 |
| ZINC02048272 | -5.6 | -0.509 | -4.4 | -0.4 | -4.3 | -0.391 | -4.2 | -0.382 |
| ZINC02048695 | -8 | -0.348 | -7 | -0.304 | -5.8 | -0.252 | -6.4 | -0.278 |
| ZINC02154763 | -7.5 | -0.375 | -5.1 | -0.255 | -5.7 | -0.285 | -5.1 | -0.255 |
| ZINC02169824 | -6.6 | -0.412 | -5.5 | -0.344 | -5.9 | -0.369 | -5.7 | -0.356 |
| ZINC02476372 | -8.8 | -0.4 | -7.6 | -0.345 | -7.2 | -0.327 | -6.8 | -0.309 |
| ZINC02515938 | -7 | -0.438 | -5.8 | -0.362 | -5.7 | -0.356 | -5.6 | -0.35 |
| ZINC02670038 | -7 | -0.304 | -6.1 | -0.265 | -6.3 | -0.274 | -6.1 | -0.265 |
| ZINC02952832 | -7.3 | -0.406 | -7.3 | -0.406 | -5.9 | -0.328 | -6.3 | -0.35 |
| ZINC03100631 | -8.8 | -0.44 | -6.7 | -0.335 | -6.8 | -0.34 | -6.8 | -0.34 |
| ZINC03157611 | -7.8 | -0.355 | -6.2 | -0.282 | -5.9 | -0.268 | -5.6 | -0.255 |
| ZINC03258337 | -8.3 | -0.553 | -6.4 | -0.427 | -5.9 | -0.393 | -6.6 | -0.44 |
| ZINC03310974 | -6.2 | -0.413 | -5.3 | -0.353 | -5.5 | -0.367 | -5.3 | -0.353 |
| ZINC03647770 | -7.7 | -0.335 | -6.8 | -0.296 | -6.2 | -0.27 | -5.8 | -0.252 |
| ZINC03830315 | -6.4 | -0.4 | -5.4 | -0.338 | -6 | -0.375 | -5.7 | -0.356 |
| ZINC03830316 | -6.7 | -0.419 | -5.3 | -0.331 | -5.8 | -0.362 | -5.1 | -0.319 |
| ZINC03831067 | -7.5 | -0.3 | -7 | -0.28 | -6.7 | -0.268 | -6.9 | -0.276 |
| ZINC03831068 | -7.3 | -0.292 | -7.1 | -0.284 | -6.5 | -0.26 | -7 | -0.28 |
| ZINC03832421_1 | -8.9 | -0.524 | -6.4 | -0.376 | -6.1 | -0.359 | -6.1 | -0.359 |
| ZINC03832421 | -8.5 | -0.5 | -6.8 | -0.4 | -6.5 | -0.382 | -6 | -0.353 |
| ZINC03845141 | -7.6 | -0.422 | -6.3 | -0.35 | -6 | -0.333 | -5.9 | -0.328 |
| ZINC03850501_1 | -6.8 | -0.486 | -6 | -0.429 | -5.3 | -0.379 | -6.3 | -0.45 |
| ZINC03850501 | -7.2 | -0.514 | -5.8 | -0.414 | -5.4 | -0.386 | -5.8 | -0.414 |
| ZINC03860856 | -7 | -0.333 | -7 | -0.333 | -6.2 | -0.295 | -6 | -0.286 |
| ZINC03860920 | -6.9 | -0.216 | -8.6 | -0.269 | -8.3 | -0.259 | -6.6 | -0.206 |
| ZINC03860960 | -8.1 | -0.476 | -6 | -0.353 | -6.1 | -0.359 | -5.6 | -0.329 |
| ZINC03875548 | -7.7 | -0.257 | -7.4 | -0.247 | -7.2 | -0.24 | -7.1 | -0.237 |
| ZINC03878829 | -6.9 | -0.431 | -5.5 | -0.344 | -5.7 | -0.356 | -5.4 | -0.338 |
| ZINC03881813 | -6.8 | -0.324 | -7.2 | -0.343 | -6.1 | -0.29 | -6.9 | -0.329 |
| ZINC03881918 | -7.9 | -0.329 | -7 | -0.292 | -6.7 | -0.279 | -6 | -0.25 |
| ZINC03884497 | -5.4 | -0.54 | -4.6 | -0.46 | -4.6 | -0.46 | -4.7 | -0.47 |
| ZINC03898665 | -7.4 | -0.285 | -7.9 | -0.304 | -7 | -0.269 | -7.2 | -0.277 |
| ZINC03898710 | -7.6 | -0.447 | -6.1 | -0.359 | -6.1 | -0.359 | -5.7 | -0.335 |
| ZINC03898822 | -8.2 | -0.39 | -6.9 | -0.329 | -6.7 | -0.319 | -6.6 | -0.314 |
| ZINC03947435 | -6.6 | -0.314 | -7.4 | -0.352 | -6.2 | -0.295 | -6.1 | -0.29 |
| ZINC03953394 | -7.5 | -0.357 | -7.6 | -0.362 | -6.8 | -0.324 | -6.1 | -0.29 |
| ZINC03953472 | -7.1 | -0.296 | -7.9 | -0.329 | -6.7 | -0.279 | -6.2 | -0.258 |
| ZINC03953805 | -7.6 | -0.447 | -6.2 | -0.365 | -5.5 | -0.324 | -5.6 | -0.329 |
| ZINC03953810 | -6.7 | -0.239 | -6.7 | -0.239 | -5.9 | -0.211 | -5.9 | -0.211 |
| ZINC03953920 | -8 | -0.364 | -6.7 | -0.305 | -6.3 | -0.286 | -5.6 | -0.255 |
| ZINC03954247 | -7.1 | -0.418 | -5.3 | -0.312 | -5.1 | -0.3 | -5 | -0.294 |
| ZINC03954311 | -7 | -0.259 | -7.5 | -0.278 | -6.6 | -0.244 | -6.7 | -0.248 |
| ZINC03954351 | -7.3 | -0.281 | -7.6 | -0.292 | -7.1 | -0.273 | -6.7 | -0.258 |
| ZINC03954397 | -8.3 | -0.319 | -7.3 | -0.281 | -6.7 | -0.258 | -6.9 | -0.265 |
| ZINC03954507 | -7.6 | -0.317 | -7 | -0.292 | -6.3 | -0.263 | -5.7 | -0.238 |
| ZINC03954520 | -7.5 | -0.278 | -7.7 | -0.285 | -7.9 | -0.293 | -7.6 | -0.281 |
| ZINC03954552 | -7.2 | -0.313 | -6.6 | -0.287 | -6 | -0.261 | -5.6 | -0.243 |
| ZINC03954626 | -7.3 | -0.429 | -6.5 | -0.382 | -6.6 | -0.388 | -6.1 | -0.359 |
| ZINC03958471 | -6.9 | -0.431 | -5.8 | -0.362 | -5.8 | -0.362 | -5.4 | -0.338 |
| ZINC04006098 | -5.9 | -0.328 | -5.5 | -0.306 | -4.9 | -0.272 | -4.8 | -0.267 |
| ZINC04015433 | -8.7 | -0.458 | -6.6 | -0.347 | -6.4 | -0.337 | -6.1 | -0.321 |
| ZINC04015715 | -7 | -0.438 | -5.5 | -0.344 | -5.5 | -0.344 | -4.9 | -0.306 |
| ZINC04027061 | -7 | -0.438 | -5.9 | -0.369 | -5.5 | -0.344 | -5.4 | -0.338 |
| ZINC04095812 | -7.5 | -0.469 | -6.3 | -0.394 | -5.6 | -0.35 | -5.9 | -0.369 |
| ZINC04127920 | -7 | -0.318 | -7.2 | -0.327 | -7 | -0.318 | -6.4 | -0.291 |
| ZINC04164657 | -7.3 | -0.348 | -6.1 | -0.29 | -6 | -0.286 | -6.1 | -0.29 |
| ZINC04212654 | -6.5 | -0.31 | -6.4 | -0.305 | -5.5 | -0.262 | -5.9 | -0.281 |
| ZINC04213833_1 | -7.5 | -0.197 | -7.2 | -0.189 | -6.2 | -0.163 | -6.6 | -0.174 |
| ZINC04213833 | -7.1 | -0.187 | -7.2 | -0.189 | -6.2 | -0.163 | -6.6 | -0.174 |
| ZINC04214344 | -7.8 | -0.3 | -8.7 | -0.335 | -7 | -0.269 | -7 | -0.269 |
| ZINC04214836 | -7.1 | -0.338 | -6.7 | -0.319 | -5.7 | -0.271 | -5.6 | -0.267 |
| ZINC04217305 | -7.7 | -0.308 | -7.7 | -0.308 | -7.3 | -0.292 | -6.5 | -0.26 |
| ZINC04217587 | -6.1 | -0.321 | -5.8 | -0.305 | -5.8 | -0.305 | -5.4 | -0.284 |
| ZINC04237573_1 | -5.1 | -0.567 | -4.3 | -0.478 | -3.8 | -0.422 | -4.6 | -0.511 |
| ZINC04237573 | -5.1 | -0.567 | -4.3 | -0.478 | -3.9 | -0.433 | -4 | -0.444 |
| ZINC04347718 | -6.7 | -0.239 | -6.7 | -0.239 | -6.4 | -0.229 | -6.3 | -0.225 |
| ZINC04353020 | -8.4 | -0.42 | -6.7 | -0.335 | -6.3 | -0.315 | -6.2 | -0.31 |
| ZINC04365786 | -8.4 | -0.382 | -6.7 | -0.305 | -6.7 | -0.305 | -6.7 | -0.305 |
| ZINC04366202 | -7.5 | -0.395 | -6.6 | -0.347 | -6.4 | -0.337 | -6.1 | -0.321 |
| ZINC04366261 | -7.5 | -0.357 | -6.2 | -0.295 | -6.1 | -0.29 | -5.9 | -0.281 |
| ZINC04366520 | -6.6 | -0.228 | -8.3 | -0.286 | -6.5 | -0.224 | -6.3 | -0.217 |
| ZINC04366893 | -8.6 | -0.43 | -6.4 | -0.32 | -5.8 | -0.29 | -5.7 | -0.285 |
| ZINC04367141 | -8.6 | -0.41 | -6.5 | -0.31 | -6.5 | -0.31 | -5.7 | -0.271 |
| ZINC04376856 | -7.2 | -0.267 | -8 | -0.296 | -8 | -0.296 | -7.4 | -0.274 |
| ZINC04403652 | -7.4 | -0.435 | -6 | -0.353 | -5.7 | -0.335 | -5.7 | -0.335 |
| ZINC04403653 | -7.5 | -0.441 | -5.7 | -0.335 | -5.6 | -0.329 | -5.6 | -0.329 |
| ZINC04409180 | -7.5 | -0.375 | -6.7 | -0.335 | -6.4 | -0.32 | -5.9 | -0.295 |
| ZINC04416197 | -7.1 | -0.254 | -7.4 | -0.264 | -6.9 | -0.246 | -6.8 | -0.243 |
| ZINC04416613 | -6.7 | -0.335 | -5.8 | -0.29 | -6 | -0.3 | -5.2 | -0.26 |
| ZINC04428312_1 | -7.3 | -0.429 | -6.1 | -0.359 | -5.8 | -0.341 | -5.6 | -0.329 |
| ZINC04428312 | -7.5 | -0.441 | -6 | -0.353 | -5.8 | -0.341 | -5.6 | -0.329 |
| ZINC04428581 | -7.6 | -0.422 | -6.4 | -0.356 | -6.5 | -0.361 | -5.7 | -0.317 |
| ZINC04428843 | -7.9 | -0.329 | -7.6 | -0.317 | -6.9 | -0.288 | -6.9 | -0.288 |
| ZINC04428858 | -6.3 | -0.42 | -6 | -0.4 | -5 | -0.333 | -5.1 | -0.34 |
| ZINC04428999 | -6.3 | -0.525 | -4.9 | -0.408 | -4.8 | -0.4 | -5.2 | -0.433 |
| ZINC04429620 | -7.2 | -0.379 | -5.9 | -0.311 | -5.9 | -0.311 | -5.6 | -0.295 |
| ZINC04430482 | -6.1 | -0.436 | -5.1 | -0.364 | -5.3 | -0.379 | -5.4 | -0.386 |
| ZINC04430753_1 | -7 | -0.467 | -5.5 | -0.367 | -5.2 | -0.347 | -4.9 | -0.327 |
| ZINC04430753 | -6.7 | -0.447 | -5.3 | -0.353 | -5.4 | -0.36 | -5.1 | -0.34 |
| ZINC04473135 | -7.7 | -0.428 | -6.7 | -0.372 | -6.7 | -0.372 | -5.9 | -0.328 |
| ZINC04522231 | -5 | -0.5 | -3.9 | -0.39 | -3.9 | -0.39 | -4.3 | -0.43 |
| ZINC04522477 | -6.3 | -0.274 | -6.5 | -0.283 | -6.1 | -0.265 | -6.1 | -0.265 |
| ZINC04531729 | -7.5 | -0.357 | -6.3 | -0.3 | -6.3 | -0.3 | -6.2 | -0.295 |
| ZINC04536747 | -6.1 | -0.407 | -5.3 | -0.353 | -4.9 | -0.327 | -4.9 | -0.327 |
| ZINC04536750 | -6.4 | -0.427 | -5.4 | -0.36 | -5.1 | -0.34 | -4.8 | -0.32 |
| ZINC04536752 | -6.1 | -0.407 | -5.1 | -0.34 | -4.8 | -0.32 | -4.8 | -0.32 |
| ZINC04537227 | -7.7 | -0.385 | -6.1 | -0.305 | -5.9 | -0.295 | -5.4 | -0.27 |
| ZINC04537229 | -7.5 | -0.375 | -5.5 | -0.275 | -5.7 | -0.285 | -5.2 | -0.26 |
| ZINC04537816 | -8.5 | -0.386 | -6.9 | -0.314 | -6 | -0.273 | -6.4 | -0.291 |
| ZINC04538450 | -7.6 | -0.345 | -7.2 | -0.327 | -6.4 | -0.291 | -6.3 | -0.286 |
| ZINC04547321 | -7.1 | -0.338 | -6.8 | -0.324 | -7.2 | -0.343 | -6.5 | -0.31 |
| ZINC04557220 | -6.8 | -0.453 | -5.6 | -0.373 | -5.7 | -0.38 | -5.6 | -0.373 |
| ZINC04558887 | -7.3 | -0.406 | -5.8 | -0.322 | -5.7 | -0.317 | -5.6 | -0.311 |
| ZINC04578913 | -7.7 | -0.428 | -5.7 | -0.317 | -5.8 | -0.322 | -5.3 | -0.294 |
| ZINC04628938 | -8.5 | -0.304 | -7.4 | -0.264 | -7.3 | -0.261 | -7.2 | -0.257 |
| ZINC04683414 | -6.9 | -0.431 | -5.9 | -0.369 | -5.5 | -0.344 | -5.3 | -0.331 |
| ZINC04691860 | -6.5 | -0.433 | -5.7 | -0.38 | -5.3 | -0.353 | -5.6 | -0.373 |
| ZINC04692015 | -7.4 | -0.224 | -7.7 | -0.233 | -6.8 | -0.206 | -6.8 | -0.206 |
| ZINC04692016 | -7.2 | -0.218 | -7.6 | -0.23 | -6.8 | -0.206 | -6.5 | -0.197 |
| ZINC04701260 | -7.4 | -0.37 | -7.2 | -0.36 | -6.4 | -0.32 | -6.3 | -0.315 |
| ZINC04701343 | -6.5 | -0.31 | -6.9 | -0.329 | -6.3 | -0.3 | -5.6 | -0.267 |
| ZINC04705802 | -7.9 | -0.343 | -6.9 | -0.3 | -7 | -0.304 | -6.5 | -0.283 |
| ZINC04706168 | -9.1 | -0.414 | -6.9 | -0.314 | -6.6 | -0.3 | -6.5 | -0.295 |
| ZINC04707417 | -6.3 | -0.45 | -5.2 | -0.371 | -5.1 | -0.364 | -4.9 | -0.35 |
| ZINC04707457 | -6.4 | -0.457 | -5.2 | -0.371 | -5.2 | -0.371 | -4.9 | -0.35 |
| ZINC04707664 | -8.1 | -0.337 | -6.9 | -0.288 | -6.4 | -0.267 | -6.7 | -0.279 |
| ZINC04707767 | -7.9 | -0.439 | -6.6 | -0.367 | -5.8 | -0.322 | -6.1 | -0.339 |
| ZINC04707792 | -6.7 | -0.258 | -6.6 | -0.254 | -5.6 | -0.215 | -5.9 | -0.227 |
| ZINC04707806 | -9.3 | -0.423 | -7.1 | -0.323 | -6.6 | -0.3 | -6 | -0.273 |
| ZINC04715161_1 | -7.2 | -0.514 | -6.2 | -0.443 | -5.6 | -0.4 | -5.8 | -0.414 |
| ZINC04715161 | -7.2 | -0.514 | -6.2 | -0.443 | -5.9 | -0.421 | -5.5 | -0.393 |
| ZINC04720969 | -8.7 | -0.322 | -6.8 | -0.252 | -6.6 | -0.244 | -6.9 | -0.256 |
| ZINC04720972 | -8.8 | -0.303 | -8 | -0.276 | -7.4 | -0.255 | -7.4 | -0.255 |
| ZINC04726283 | -7.9 | -0.376 | -6.6 | -0.314 | -5.9 | -0.281 | -5.4 | -0.257 |
| ZINC04726284 | -8.2 | -0.39 | -6.9 | -0.329 | -6.2 | -0.295 | -5.8 | -0.276 |
| ZINC04726655 | -6.5 | -0.464 | -5 | -0.357 | -4.8 | -0.343 | -4.7 | -0.336 |
| ZINC04726869 | -7.3 | -0.261 | -5.4 | -0.193 | -5.1 | -0.182 | -5.5 | -0.196 |
| ZINC04742623 | -7.5 | -0.375 | -6.7 | -0.335 | -5.8 | -0.29 | -5.8 | -0.29 |
| ZINC04743527 | -6.7 | -0.419 | -6.9 | -0.431 | -6.1 | -0.381 | -5.7 | -0.356 |
| ZINC04744282 | -6 | -0.4 | -5 | -0.333 | -4.8 | -0.32 | -4.4 | -0.293 |
| ZINC04747919 | -9.6 | -0.457 | -6.8 | -0.324 | -6.8 | -0.324 | -6 | -0.286 |
| ZINC04748128 | -6.9 | -0.46 | -5.4 | -0.36 | -5.4 | -0.36 | -5.5 | -0.367 |
| ZINC04748141 | -8.5 | -0.327 | -7 | -0.269 | -6.8 | -0.262 | -6.6 | -0.254 |
| ZINC04758411 | -8.5 | -0.386 | -7.3 | -0.332 | -6.5 | -0.295 | -5.9 | -0.268 |
| ZINC04760065 | -6.5 | -0.433 | -5.6 | -0.373 | -5.3 | -0.353 | -4.8 | -0.32 |
| ZINC04769176 | -7.8 | -0.355 | -7.1 | -0.323 | -6.7 | -0.305 | -6.1 | -0.277 |
| ZINC04769447 | -7 | -0.467 | -5.8 | -0.387 | -5.3 | -0.353 | -5.9 | -0.393 |
| ZINC04769448 | -6.4 | -0.427 | -5.9 | -0.393 | -5.9 | -0.393 | -5.9 | -0.393 |
| ZINC04769600 | -7.7 | -0.321 | -6.9 | -0.288 | -6.9 | -0.288 | -6 | -0.25 |
| ZINC04771299 | -7.5 | -0.375 | -6.2 | -0.31 | -6 | -0.3 | -6.2 | -0.31 |
| ZINC04773602 | -7.8 | -0.289 | -8.3 | -0.307 | -7.6 | -0.281 | -7 | -0.259 |
| ZINC04775687 | -6.1 | -0.469 | -5.2 | -0.4 | -5.2 | -0.4 | -4.7 | -0.362 |
| ZINC04775707 | -6.3 | -0.485 | -5.1 | -0.392 | -5 | -0.385 | -5.3 | -0.408 |
| ZINC04775893 | -6.6 | -0.412 | -5.6 | -0.35 | -5.8 | -0.362 | -5.2 | -0.325 |
| ZINC04776634 | -7.7 | -0.405 | -5.9 | -0.311 | -5.9 | -0.311 | -5.6 | -0.295 |
| ZINC04777715 | -6.2 | -0.477 | -5.2 | -0.4 | -4.8 | -0.369 | -5.4 | -0.415 |
| ZINC04777716 | -5.9 | -0.454 | -5.3 | -0.408 | -4.9 | -0.377 | -5.7 | -0.438 |
| ZINC04777790 | -8.1 | -0.405 | -6.4 | -0.32 | -6.2 | -0.31 | -6.2 | -0.31 |
| ZINC04777791 | -7.7 | -0.385 | -6.9 | -0.345 | -6.3 | -0.315 | -5.8 | -0.29 |
| ZINC04777934 | -6.9 | -0.23 | -7.5 | -0.25 | -6.6 | -0.22 | -6.5 | -0.217 |
| ZINC04777935 | -7.8 | -0.26 | -7.3 | -0.243 | -6.6 | -0.22 | -6.4 | -0.213 |
| ZINC04777936 | -7.3 | -0.243 | -6.6 | -0.22 | -6.5 | -0.217 | -6.3 | -0.21 |
| ZINC04777937 | -7.3 | -0.243 | -7.2 | -0.24 | -7 | -0.233 | -6.4 | -0.213 |
| ZINC04783080 | -7.3 | -0.384 | -6 | -0.316 | -6 | -0.316 | -5.2 | -0.274 |
| ZINC04783140 | -7.2 | -0.45 | -5.6 | -0.35 | -5.5 | -0.344 | -5 | -0.312 |
| ZINC04783204 | -7.4 | -0.389 | -6.7 | -0.353 | -6.1 | -0.321 | -6.3 | -0.332 |
| ZINC04783229 | -8.9 | -0.247 | -8.4 | -0.233 | -8.3 | -0.231 | -8.5 | -0.236 |
| ZINC04783478 | -7.4 | -0.336 | -6.6 | -0.3 | -5.8 | -0.264 | -5.8 | -0.264 |
| ZINC04783479 | -7.6 | -0.345 | -6.6 | -0.3 | -6.1 | -0.277 | -6 | -0.273 |
| ZINC04783480 | -7.5 | -0.341 | -7.1 | -0.323 | -6.5 | -0.295 | -5.6 | -0.255 |
| ZINC04783481 | -7.7 | -0.35 | -6.7 | -0.305 | -5.5 | -0.25 | -5.8 | -0.264 |
| ZINC04786808 | -6 | -0.429 | -5.8 | -0.414 | -5 | -0.357 | -5.2 | -0.371 |
| ZINC04786811 | -6.2 | -0.443 | -5.4 | -0.386 | -5 | -0.357 | -5.4 | -0.386 |
| ZINC04786814 | -6.2 | -0.443 | -5.3 | -0.379 | -4.9 | -0.35 | -5.5 | -0.393 |
| ZINC04791992 | -8.8 | -0.463 | -7 | -0.368 | -6.4 | -0.337 | -6.1 | -0.321 |
| ZINC04797042 | -8.3 | -0.332 | -6.7 | -0.268 | -6 | -0.24 | -6.2 | -0.248 |
| ZINC04800001 | -8.1 | -0.386 | -7.3 | -0.348 | -7.3 | -0.348 | -6.8 | -0.324 |
| ZINC04821670 | -7.3 | -0.406 | -6.2 | -0.344 | -6.1 | -0.339 | -5.8 | -0.322 |
| ZINC04821957 | -6.6 | -0.347 | -6.8 | -0.358 | -6.1 | -0.321 | -5.9 | -0.311 |
| ZINC04822288_1 | -8.2 | -0.357 | -7.5 | -0.326 | -6.9 | -0.3 | -6.4 | -0.278 |
| ZINC04822288 | -8.4 | -0.365 | -7.2 | -0.313 | -7.1 | -0.309 | -6.4 | -0.278 |
| ZINC04822383 | -7.7 | -0.367 | -6.3 | -0.3 | -6.4 | -0.305 | -6.4 | -0.305 |
| ZINC04822746 | -7.9 | -0.359 | -6 | -0.273 | -5.9 | -0.268 | -5.6 | -0.255 |
| ZINC04822747 | -7.8 | -0.355 | -6 | -0.273 | -5.5 | -0.25 | -5.4 | -0.245 |
| ZINC04823467 | -7.3 | -0.261 | -7.4 | -0.264 | -6.8 | -0.243 | -6.6 | -0.236 |
| ZINC04824022_1 | -7.6 | -0.447 | -6.2 | -0.365 | -5.6 | -0.329 | -6.1 | -0.359 |
| ZINC04824022 | -7.7 | -0.453 | -6.3 | -0.371 | -5.5 | -0.324 | -6.1 | -0.359 |
| ZINC04824024_1 | -7.5 | -0.441 | -5.8 | -0.341 | -5.8 | -0.341 | -5.4 | -0.318 |
| ZINC04824024 | -7.6 | -0.447 | -5.8 | -0.341 | -5.8 | -0.341 | -5.8 | -0.341 |
| ZINC04824645 | -7.8 | -0.26 | -8.7 | -0.29 | -8 | -0.267 | -7.6 | -0.253 |
| ZINC04824902 | -7.8 | -0.312 | -6.5 | -0.26 | -6.6 | -0.264 | -6 | -0.24 |
| ZINC04830956 | -6.9 | -0.345 | -6.5 | -0.325 | -6.2 | -0.31 | -5.9 | -0.295 |
| ZINC04831099 | -7.5 | -0.441 | -5.6 | -0.329 | -5.9 | -0.347 | -5.8 | -0.341 |
| ZINC04877299_1 | -6.9 | -0.46 | -6.6 | -0.44 | -5.7 | -0.38 | -5.9 | -0.393 |
| ZINC04877299 | -7.3 | -0.487 | -6.6 | -0.44 | -5.7 | -0.38 | -5.9 | -0.393 |
| ZINC04878491_1 | -8.4 | -0.35 | -7.2 | -0.3 | -6.9 | -0.288 | -6.6 | -0.275 |
| ZINC04878491 | -8.5 | -0.354 | -7.3 | -0.304 | -6.8 | -0.283 | -6.4 | -0.267 |
| ZINC04879418_1 | -6.3 | -0.525 | -4.9 | -0.408 | -5.1 | -0.425 | -5.4 | -0.45 |
| ZINC04879418 | -6.2 | -0.517 | -4.8 | -0.4 | -5 | -0.417 | -5.3 | -0.442 |
| ZINC04887389 | -7.8 | -0.355 | -7.7 | -0.35 | -6.6 | -0.3 | -6.5 | -0.295 |
| ZINC04887558 | -9.5 | -0.432 | -6.7 | -0.305 | -6.9 | -0.314 | -6.3 | -0.286 |
| ZINC04896131 | -5.9 | -0.227 | -6.5 | -0.25 | -6.1 | -0.235 | -6.4 | -0.246 |
| ZINC04896472 | -9.7 | -0.388 | -8.6 | -0.344 | -8.1 | -0.324 | -7.3 | -0.292 |
| ZINC04896601 | -9.5 | -0.244 | -7.9 | -0.203 | -7.7 | -0.197 | -7.8 | -0.2 |
| ZINC04900076 | -6.4 | -0.427 | -5.2 | -0.347 | -5 | -0.333 | -4.9 | -0.327 |
| ZINC04900874 | -9 | -0.346 | -7.8 | -0.3 | -7.3 | -0.281 | -7.2 | -0.277 |
| ZINC04901375 | -8.3 | -0.437 | -7.2 | -0.379 | -7.1 | -0.374 | -6.5 | -0.342 |
| ZINC04918956 | -8.2 | -0.222 | -8.3 | -0.224 | -6.8 | -0.184 | -7.1 | -0.192 |
| ZINC04918963 | -7.8 | -0.211 | -8.1 | -0.219 | -6.8 | -0.184 | -6.9 | -0.186 |
| ZINC04918969 | -7.7 | -0.208 | -7.2 | -0.195 | -6.4 | -0.173 | -6.6 | -0.178 |
| ZINC04934182 | -7.5 | -0.395 | -6.7 | -0.353 | -6.1 | -0.321 | -5.8 | -0.305 |
| ZINC04962003 | -7.2 | -0.4 | -6.2 | -0.344 | -5.4 | -0.3 | -5.4 | -0.3 |
| ZINC04963162 | -6.5 | -0.464 | -5.2 | -0.371 | -4.9 | -0.35 | -5.3 | -0.379 |
| ZINC04963453 | -8.4 | -0.467 | -6.5 | -0.361 | -6.5 | -0.361 | -6.2 | -0.344 |
| ZINC04963778 | -7.3 | -0.348 | -7.1 | -0.338 | -7 | -0.333 | -6.5 | -0.31 |
| ZINC04963779 | -7.2 | -0.343 | -7 | -0.333 | -7.1 | -0.338 | -6.2 | -0.295 |
| ZINC04974338 | -4.9 | -0.445 | -4.3 | -0.391 | -4.2 | -0.382 | -3.9 | -0.355 |
| ZINC04974725 | -7.2 | -0.343 | -6.4 | -0.305 | -5.9 | -0.281 | -5.6 | -0.267 |
| ZINC04984366 | -7.8 | -0.371 | -5.8 | -0.276 | -5.6 | -0.267 | -5.3 | -0.252 |
| ZINC04990829 | -5.5 | -0.458 | -5.3 | -0.442 | -4.6 | -0.383 | -4.7 | -0.392 |
| ZINC05011996 | -6.6 | -0.388 | -6.1 | -0.359 | -5.6 | -0.329 | -5.7 | -0.335 |
| ZINC05011999 | -6.1 | -0.407 | -5.6 | -0.373 | -5.4 | -0.36 | -5.2 | -0.347 |
| ZINC05015095 | -7.4 | -0.285 | -8 | -0.308 | -7 | -0.269 | -6.4 | -0.246 |
| ZINC05027641_1 | -6.3 | -0.525 | -5.3 | -0.442 | -4.9 | -0.408 | -6.1 | -0.508 |
| ZINC05027641 | -6.4 | -0.533 | -5.1 | -0.425 | -4.9 | -0.408 | -6.3 | -0.525 |
| ZINC05030617 | -5.8 | -0.483 | -4.5 | -0.375 | -4.5 | -0.375 | -5 | -0.417 |
| ZINC05033796 | -7 | -0.292 | -7.7 | -0.321 | -6.8 | -0.283 | -6.6 | -0.275 |
| ZINC05064462 | -7.9 | -0.395 | -6.4 | -0.32 | -6.1 | -0.305 | -5.8 | -0.29 |
| ZINC05086138 | -7.1 | -0.338 | -7.5 | -0.357 | -6.3 | -0.3 | -6.2 | -0.295 |
| ZINC05086225 | -7.1 | -0.444 | -5.4 | -0.338 | -5 | -0.312 | -4.8 | -0.3 |
| ZINC05105895 | -6.8 | -0.243 | -6.5 | -0.232 | -5.8 | -0.207 | -5.8 | -0.207 |
| ZINC05114817_1 | -7.5 | -0.326 | -6.7 | -0.291 | -6.5 | -0.283 | -6.6 | -0.287 |
| ZINC05114817 | -7.8 | -0.339 | -6.6 | -0.287 | -6.4 | -0.278 | -6.4 | -0.278 |
| ZINC05115990_1 | -6.7 | -0.394 | -4.9 | -0.288 | -5.5 | -0.324 | -5.1 | -0.3 |
| ZINC05115990 | -6.8 | -0.4 | -4.9 | -0.288 | -5.5 | -0.324 | -5.1 | -0.3 |
| ZINC05120864 | -8.6 | -0.43 | -6.5 | -0.325 | -6.2 | -0.31 | -6.3 | -0.315 |
| ZINC05120916 | -8.7 | -0.378 | -6.3 | -0.274 | -5.8 | -0.252 | -6.3 | -0.274 |
| ZINC05120920_1 | -8.4 | -0.467 | -6.7 | -0.372 | -6.1 | -0.339 | -5.9 | -0.328 |
| ZINC05120920 | -8.6 | -0.478 | -6.6 | -0.367 | -6.1 | -0.339 | -6.2 | -0.344 |
| ZINC05124931 | -8.8 | -0.293 | -7.7 | -0.257 | -7.1 | -0.237 | -6.4 | -0.213 |
| ZINC05124957 | -8.9 | -0.342 | -7.6 | -0.292 | -7.3 | -0.281 | -7 | -0.269 |
| ZINC05124960 | -8.7 | -0.322 | -7.8 | -0.289 | -6.9 | -0.256 | -6.7 | -0.248 |
| ZINC05137909 | -6.6 | -0.412 | -5.4 | -0.338 | -5.4 | -0.338 | -5 | -0.312 |
| ZINC05176310 | -5.8 | -0.446 | -5 | -0.385 | -4.9 | -0.377 | -5.6 | -0.431 |
| ZINC05180959 | -7.8 | -0.39 | -6.2 | -0.31 | -6.4 | -0.32 | -5.7 | -0.285 |
| ZINC05193477 | -4.5 | -0.45 | -4.4 | -0.44 | -3.8 | -0.38 | -4.1 | -0.41 |
| ZINC05201470 | -6.7 | -0.353 | -5.4 | -0.284 | -4.9 | -0.258 | -5 | -0.263 |
| ZINC05208303 | -7.8 | -0.339 | -6.3 | -0.274 | -5.8 | -0.252 | -5.8 | -0.252 |
| ZINC05217440 | -6.2 | -0.477 | -5 | -0.385 | -4.7 | -0.362 | -4.5 | -0.346 |
| ZINC05217831 | -7.7 | -0.275 | -6.6 | -0.236 | -6.6 | -0.236 | -6.1 | -0.218 |
| ZINC05220390 | -6.5 | -0.464 | -5.6 | -0.4 | -5 | -0.357 | -5.1 | -0.364 |
| ZINC05279843_1 | -6.7 | -0.447 | -6.6 | -0.44 | -5.5 | -0.367 | -5.5 | -0.367 |
| ZINC05279843 | -7 | -0.467 | -6.3 | -0.42 | -5.5 | -0.367 | -5.4 | -0.36 |
| ZINC05328871 | -8.8 | -0.367 | -6.6 | -0.275 | -6.4 | -0.267 | -6.3 | -0.263 |
| ZINC05369992 | -7.1 | -0.296 | -7.1 | -0.296 | -7 | -0.292 | -6.3 | -0.263 |
| ZINC05386901 | -8.3 | -0.415 | -6.9 | -0.345 | -7.1 | -0.355 | -6.4 | -0.32 |
| ZINC05390066 | -7.4 | -0.493 | -6.2 | -0.413 | -5.7 | -0.38 | -5.7 | -0.38 |
| ZINC05390471 | -9.1 | -0.314 | -7.4 | -0.255 | -8 | -0.276 | -8.1 | -0.279 |
| ZINC05392913 | -6.2 | -0.295 | -6.7 | -0.319 | -6.2 | -0.295 | -6.2 | -0.295 |
| ZINC05420911 | -6.9 | -0.363 | -5.5 | -0.289 | -5.4 | -0.284 | -5.2 | -0.274 |
| ZINC05421282 | -7.2 | -0.424 | -6.6 | -0.388 | -5.8 | -0.341 | -5.9 | -0.347 |
| ZINC05421739 | -5.6 | -0.431 | -5.1 | -0.392 | -4.6 | -0.354 | -4.9 | -0.377 |
| ZINC05422109_1 | -8.2 | -0.547 | -6.1 | -0.407 | -5.6 | -0.373 | -5.7 | -0.38 |
| ZINC05422109 | -8.4 | -0.56 | -6.1 | -0.407 | -5.7 | -0.38 | -5.7 | -0.38 |
| ZINC05424472 | -7.4 | -0.389 | -5.9 | -0.311 | -5.5 | -0.289 | -5.2 | -0.274 |
| ZINC05424474 | -7.4 | -0.389 | -5.7 | -0.3 | -5.4 | -0.284 | -5.2 | -0.274 |
| ZINC05424691 | -6.4 | -0.427 | -5.1 | -0.34 | -5.2 | -0.347 | -5.3 | -0.353 |
| ZINC05430677 | -7.1 | -0.418 | -5.8 | -0.341 | -5.7 | -0.335 | -6.1 | -0.359 |
| ZINC05434498 | -7.5 | -0.441 | -6.3 | -0.371 | -5.8 | -0.341 | -5.6 | -0.329 |
| ZINC05439444 | -5.4 | -0.45 | -4.7 | -0.392 | -4.3 | -0.358 | -4.1 | -0.342 |
| ZINC05452603 | -6.7 | -0.419 | -5.4 | -0.338 | -5.1 | -0.319 | -5.7 | -0.356 |
| ZINC05459826 | -7.6 | -0.345 | -6.2 | -0.282 | -5.8 | -0.264 | -5.5 | -0.25 |
| ZINC05462670 | -8.4 | -0.21 | -8.6 | -0.215 | -6.8 | -0.17 | -7 | -0.175 |
| ZINC05462674 | -8.5 | -0.212 | -8.6 | -0.215 | -6.9 | -0.173 | -6.9 | -0.173 |
| ZINC05479118 | -9.2 | -0.46 | -6.5 | -0.325 | -6.8 | -0.34 | -6.4 | -0.32 |
| ZINC05479451 | -7.6 | -0.38 | -5.7 | -0.285 | -6.1 | -0.305 | -5.6 | -0.28 |
| ZINC05487838_1 | -8.6 | -0.319 | -7.1 | -0.263 | -7.3 | -0.27 | -7.1 | -0.263 |
| ZINC05487838 | -8.9 | -0.33 | -7.7 | -0.285 | -7.3 | -0.27 | -7.3 | -0.27 |
| ZINC05492794 | -8.2 | -0.182 | -8.4 | -0.187 | -7.9 | -0.176 | -7.9 | -0.176 |
| ZINC05493068 | -7 | -0.35 | -5.6 | -0.28 | -5.7 | -0.285 | -5.5 | -0.275 |
| ZINC05493736 | -8.4 | -0.365 | -7.6 | -0.33 | -6.6 | -0.287 | -6.5 | -0.283 |
| ZINC05498636 | -7.2 | -0.288 | -6.8 | -0.272 | -6.1 | -0.244 | -6.1 | -0.244 |
| ZINC05499702 | -7.4 | -0.285 | -6.5 | -0.25 | -6.4 | -0.246 | -6.1 | -0.235 |
| ZINC05499876 | -6.8 | -0.34 | -6.2 | -0.31 | -5.6 | -0.28 | -5.5 | -0.275 |
| ZINC05502368_1 | -6.6 | -0.508 | -5.4 | -0.415 | -4.9 | -0.377 | -6.3 | -0.485 |
| ZINC05502368 | -6.5 | -0.5 | -5.1 | -0.392 | -4.9 | -0.377 | -5.7 | -0.438 |
| ZINC05513820 | -4.7 | -0.47 | -4.1 | -0.41 | -3.8 | -0.38 | -4.2 | -0.42 |
| ZINC05518987 | -8.2 | -0.41 | -6.6 | -0.33 | -6.3 | -0.315 | -6 | -0.3 |
| ZINC05536814 | -7.3 | -0.27 | -7.5 | -0.278 | -6.8 | -0.252 | -7 | -0.259 |
| ZINC05541927 | -5.8 | -0.58 | -4.7 | -0.47 | -4.5 | -0.45 | -4.9 | -0.49 |
| ZINC05541929 | -5.6 | -0.56 | -4.7 | -0.47 | -4.4 | -0.44 | -5.2 | -0.52 |
| ZINC05541931 | -5.8 | -0.58 | -4.6 | -0.46 | -4.4 | -0.44 | -5.2 | -0.52 |
| ZINC05541933 | -5.8 | -0.58 | -4.4 | -0.44 | -4.6 | -0.46 | -4.9 | -0.49 |
| ZINC05550368 | -6.8 | -0.358 | -6 | -0.316 | -5.7 | -0.3 | -5.7 | -0.3 |
| ZINC05550663 | -7.1 | -0.338 | -6.1 | -0.29 | -5.4 | -0.257 | -5.4 | -0.257 |
| ZINC05550666 | -7.3 | -0.348 | -5.5 | -0.262 | -5.7 | -0.271 | -5.4 | -0.257 |
| ZINC05551091_1 | -6.3 | -0.371 | -5.4 | -0.318 | -5.2 | -0.306 | -4.8 | -0.282 |
| ZINC05551091 | -6.4 | -0.376 | -5.7 | -0.335 | -5.5 | -0.324 | -5.1 | -0.3 |
| ZINC05551760_1 | -6.4 | -0.376 | -5.3 | -0.312 | -5.1 | -0.3 | -5.1 | -0.3 |
| ZINC05551760 | -6.8 | -0.4 | -5.4 | -0.318 | -5.3 | -0.312 | -5.2 | -0.306 |
| ZINC05552274_1 | -6.3 | -0.485 | -5 | -0.385 | -5.1 | -0.392 | -5.3 | -0.408 |
| ZINC05552274 | -6.3 | -0.485 | -4.9 | -0.377 | -4.8 | -0.369 | -4.8 | -0.369 |
| ZINC05554415 | -7.7 | -0.405 | -6.2 | -0.326 | -5.5 | -0.289 | -5.8 | -0.305 |
| ZINC05554417 | -7.9 | -0.416 | -5.7 | -0.3 | -5.7 | -0.3 | -5.2 | -0.274 |
| ZINC05575116 | -5.6 | -0.467 | -4.6 | -0.383 | -4.5 | -0.375 | -4.9 | -0.408 |
| ZINC05576272 | -7 | -0.412 | -5.8 | -0.341 | -6 | -0.353 | -5.4 | -0.318 |
| ZINC05580556 | -7.9 | -0.439 | -6.1 | -0.339 | -6 | -0.333 | -6.1 | -0.339 |
| ZINC05580600 | -8 | -0.333 | -7.2 | -0.3 | -7.1 | -0.296 | -6.6 | -0.275 |
| ZINC05580712 | -7.7 | -0.428 | -6.4 | -0.356 | -6.1 | -0.339 | -5.9 | -0.328 |
| ZINC05581222 | -6.7 | -0.319 | -5.9 | -0.281 | -5.9 | -0.281 | -5.7 | -0.271 |
| ZINC05581974 | -7.1 | -0.394 | -6.1 | -0.339 | -6.2 | -0.344 | -5.8 | -0.322 |
| ZINC05594334 | -6.7 | -0.479 | -5.8 | -0.414 | -5.1 | -0.364 | -5 | -0.357 |
| ZINC05640172 | -6.8 | -0.309 | -7.1 | -0.323 | -6.3 | -0.286 | -5.8 | -0.264 |
| ZINC05640410 | -7.8 | -0.411 | -6.4 | -0.337 | -6.2 | -0.326 | -6.4 | -0.337 |
| ZINC05640600 | -7.5 | -0.441 | -6.1 | -0.359 | -6 | -0.353 | -5.6 | -0.329 |
| ZINC05640620 | -7.6 | -0.33 | -7.3 | -0.317 | -6.3 | -0.274 | -6.2 | -0.27 |
| ZINC05641037 | -8.1 | -0.337 | -6.8 | -0.283 | -7.3 | -0.304 | -6.4 | -0.267 |
| ZINC05647206_1 | -8.5 | -0.425 | -7 | -0.35 | -6.7 | -0.335 | -6.7 | -0.335 |
| ZINC05647206 | -9 | -0.45 | -7 | -0.35 | -6.7 | -0.335 | -6.6 | -0.33 |
| ZINC05662861 | -8.5 | -0.354 | -7 | -0.292 | -7.1 | -0.296 | -5.8 | -0.242 |
| ZINC05664697 | -9 | -0.243 | -8.2 | -0.222 | -8 | -0.216 | -7.6 | -0.205 |
| ZINC05665089 | -7.6 | -0.33 | -7 | -0.304 | -7 | -0.304 | -7 | -0.304 |
| ZINC05709639 | -7.4 | -0.285 | -7.3 | -0.281 | -6.4 | -0.246 | -6.4 | -0.246 |
| ZINC05723045 | -7.6 | -0.4 | -6.4 | -0.337 | -6.3 | -0.332 | -6.3 | -0.332 |
| ZINC05723051 | -8 | -0.4 | -6.7 | -0.335 | -6.4 | -0.32 | -6.1 | -0.305 |
| ZINC05732213 | -6.4 | -0.305 | -5.4 | -0.257 | -5.5 | -0.262 | -5.1 | -0.243 |
| ZINC05782788 | -7.7 | -0.35 | -6.3 | -0.286 | -6.2 | -0.282 | -5.3 | -0.241 |
| ZINC05785035 | -5.6 | -0.509 | -4.6 | -0.418 | -4.5 | -0.409 | -5.2 | -0.473 |
| ZINC05810714 | -7.8 | -0.459 | -6.2 | -0.365 | -5.9 | -0.347 | -6 | -0.353 |
| ZINC05810741 | -6.7 | -0.372 | -5.4 | -0.3 | -5.2 | -0.289 | -5.3 | -0.294 |
| ZINC05824155 | -7.5 | -0.417 | -6.2 | -0.344 | -6.2 | -0.344 | -5.9 | -0.328 |
| ZINC05834946 | -5.5 | -0.458 | -4.4 | -0.367 | -3.9 | -0.325 | -4.2 | -0.35 |
| ZINC05844151 | -6.7 | -0.319 | -6.6 | -0.314 | -6.3 | -0.3 | -6.2 | -0.295 |
| ZINC05979393 | -7.7 | -0.35 | -6.1 | -0.277 | -6 | -0.273 | -5.5 | -0.25 |
| ZINC05993141 | -7 | -0.467 | -5.4 | -0.36 | -5 | -0.333 | -5.1 | -0.34 |
| ZINC05997896 | -7.6 | -0.422 | -7.2 | -0.4 | -6.1 | -0.339 | -6.4 | -0.356 |
| ZINC06092501 | -7.5 | -0.417 | -6.2 | -0.344 | -5.8 | -0.322 | -5.8 | -0.322 |
| ZINC06092503 | -7.9 | -0.439 | -6 | -0.333 | -5.9 | -0.328 | -5.8 | -0.322 |
| ZINC06095736 | -6 | -0.5 | -5.4 | -0.45 | -5.1 | -0.425 | -5.7 | -0.475 |
| ZINC06188101_1 | -6.5 | -0.406 | -5.4 | -0.338 | -5.6 | -0.35 | -5.8 | -0.362 |
| ZINC06188101 | -6.8 | -0.425 | -5.5 | -0.344 | -5.6 | -0.35 | -5.9 | -0.369 |
| ZINC06343280 | -8.1 | -0.324 | -7.9 | -0.316 | -7.1 | -0.284 | -7 | -0.28 |
| ZINC06381592 | -7.6 | -0.281 | -6.9 | -0.256 | -7.1 | -0.263 | -7 | -0.259 |
| ZINC06511573 | -7.1 | -0.338 | -6.5 | -0.31 | -6.5 | -0.31 | -5.8 | -0.276 |
| ZINC06511656_1 | -8.9 | -0.356 | -7 | -0.28 | -6.6 | -0.264 | -7.1 | -0.284 |
| ZINC06511656 | -9 | -0.36 | -7 | -0.28 | -7.1 | -0.284 | -7.1 | -0.284 |
| ZINC06511739 | -5.7 | -0.518 | -4.9 | -0.445 | -5.1 | -0.464 | -5 | -0.455 |
| ZINC06511825 | -8.3 | -0.377 | -6.6 | -0.3 | -6.7 | -0.305 | -6.4 | -0.291 |
| ZINC06513204 | -5.9 | -0.536 | -5 | -0.455 | -4.8 | -0.436 | -4.7 | -0.427 |
| ZINC06513918 | -7.5 | -0.357 | -6.7 | -0.319 | -6.8 | -0.324 | -6.2 | -0.295 |
| ZINC06520320 | -6.6 | -0.3 | -7.2 | -0.327 | -6.3 | -0.286 | -6.1 | -0.277 |
| ZINC06576021 | -8.3 | -0.415 | -7.2 | -0.36 | -6.6 | -0.33 | -6.2 | -0.31 |
| ZINC06576323 | -8.2 | -0.373 | -7 | -0.318 | -6.9 | -0.314 | -6.5 | -0.295 |
| ZINC06576501 | -9 | -0.429 | -6.7 | -0.319 | -6.7 | -0.319 | -6.3 | -0.3 |
| ZINC06857068 | -6.6 | -0.471 | -5.3 | -0.379 | -5.1 | -0.364 | -6 | -0.429 |
| ZINC07996353 | -7.5 | -0.395 | -5.9 | -0.311 | -6.1 | -0.321 | -5 | -0.263 |
| ZINC08553055 | -6.3 | -0.42 | -5.6 | -0.373 | -5.2 | -0.347 | -5.2 | -0.347 |
| ZINC08560067_1 | -8.6 | -0.41 | -6.2 | -0.295 | -6.6 | -0.314 | -6.6 | -0.314 |
| ZINC08560067 | -8.5 | -0.405 | -6 | -0.286 | -6.6 | -0.314 | -5.7 | -0.271 |
| ZINC08580873 | -5.8 | -0.414 | -5.2 | -0.371 | -5 | -0.357 | -4.9 | -0.35 |
| ZINC08581164 | -7.7 | -0.481 | -5.7 | -0.356 | -5.2 | -0.325 | -5.5 | -0.344 |
| ZINC08581166 | -7.5 | -0.469 | -5.6 | -0.35 | -5.1 | -0.319 | -5.4 | -0.338 |
| ZINC08581317 | -8.7 | -0.414 | -7 | -0.333 | -6.4 | -0.305 | -6.3 | -0.3 |
| ZINC08581421 | -6.4 | -0.457 | -5.1 | -0.364 | -5.2 | -0.371 | -4.9 | -0.35 |
| ZINC08581460 | -6.8 | -0.234 | -7.8 | -0.269 | -6.2 | -0.214 | -6.2 | -0.214 |
| ZINC08585953 | -8.2 | -0.315 | -7.4 | -0.285 | -6.7 | -0.258 | -6.8 | -0.262 |
| ZINC08603298 | -5.2 | -0.578 | -4.5 | -0.5 | -4.7 | -0.522 | -5.1 | -0.567 |
| ZINC08613703 | -6.9 | -0.46 | -5.8 | -0.387 | -5.6 | -0.373 | -5.4 | -0.36 |
| ZINC08615383 | -6.5 | -0.464 | -4.7 | -0.336 | -4.8 | -0.343 | -4.8 | -0.343 |
| ZINC08615531 | -8.1 | -0.45 | -6.6 | -0.367 | -6.6 | -0.367 | -6.3 | -0.35 |
| ZINC08615799 | -6.7 | -0.372 | -6.3 | -0.35 | -5.4 | -0.3 | -5.3 | -0.294 |
| ZINC08616208 | -4.9 | -0.613 | -4.1 | -0.512 | -4.2 | -0.525 | -4.5 | -0.562 |
| ZINC08617619 | -6.7 | -0.479 | -6.1 | -0.436 | -5.8 | -0.414 | -5.9 | -0.421 |
| ZINC08618545 | -8.6 | -0.43 | -6.7 | -0.335 | -6.5 | -0.325 | -6 | -0.3 |
| ZINC08618968 | -6.2 | -0.477 | -5.1 | -0.392 | -5.1 | -0.392 | -4.8 | -0.369 |
| ZINC08622475 | -6.1 | -0.469 | -5 | -0.385 | -5.1 | -0.392 | -5.4 | -0.415 |
| ZINC08627485 | -8 | -0.444 | -6 | -0.333 | -6 | -0.333 | -5.5 | -0.306 |
| ZINC08627502 | -8.2 | -0.41 | -6.6 | -0.33 | -6.7 | -0.335 | -6.6 | -0.33 |
| ZINC08637986_1 | -6 | -0.429 | -4.8 | -0.343 | -4.8 | -0.343 | -5.7 | -0.407 |
| ZINC08637986 | -6 | -0.429 | -4.9 | -0.35 | -4.8 | -0.343 | -5.6 | -0.4 |
| ZINC08648354 | -8.8 | -0.314 | -7.1 | -0.254 | -6.9 | -0.246 | -6.6 | -0.236 |
| ZINC08649723 | -8 | -0.364 | -7.5 | -0.341 | -6.3 | -0.286 | -6.5 | -0.295 |
| ZINC08649758 | -7.4 | -0.336 | -6.6 | -0.3 | -6.5 | -0.295 | -6.3 | -0.286 |
| ZINC08652230 | -7.9 | -0.527 | -6.6 | -0.44 | -6 | -0.4 | -6.9 | -0.46 |
| ZINC08655995 | -7.9 | -0.416 | -6.4 | -0.337 | -6 | -0.316 | -6 | -0.316 |
| ZINC08660420 | -7.5 | -0.259 | -7.2 | -0.248 | -6.9 | -0.238 | -6.9 | -0.238 |
| ZINC08672844 | -5.2 | -0.578 | -4.5 | -0.5 | -4.1 | -0.456 | -4.4 | -0.489 |
| ZINC08772958 | -7.2 | -0.343 | -6.8 | -0.324 | -6.4 | -0.305 | -6.3 | -0.3 |
| ZINC09211296 | -6.9 | -0.431 | -5.9 | -0.369 | -5.4 | -0.338 | -5.2 | -0.325 |
| ZINC09230252 | -8.2 | -0.283 | -7 | -0.241 | -6.2 | -0.214 | -6.2 | -0.214 |
| ZINC11525688 | -7.3 | -0.521 | -6.3 | -0.45 | -6 | -0.429 | -6.8 | -0.486 |
| ZINC11535692 | -6.8 | -0.486 | -5.7 | -0.407 | -5 | -0.357 | -5 | -0.357 |
| ZINC11535830 | -7 | -0.5 | -5.7 | -0.407 | -5.1 | -0.364 | -5.2 | -0.371 |
| ZINC11535850 | -6 | -0.5 | -4.8 | -0.4 | -4.6 | -0.383 | -5.2 | -0.433 |
| ZINC11616854 | -8.8 | -0.191 | -8.1 | -0.176 | -7.8 | -0.17 | -7 | -0.152 |
| ZINC11616855 | -7.7 | -0.167 | -8 | -0.174 | -7.1 | -0.154 | -7.1 | -0.154 |
| ZINC11616856 | -8.4 | -0.183 | -8.1 | -0.176 | -7.4 | -0.161 | -7.5 | -0.163 |
| ZINC11616857 | -7.8 | -0.17 | -7.5 | -0.163 | -7 | -0.152 | -7 | -0.152 |
| ZINC11677161 | -7.1 | -0.197 | -7.7 | -0.214 | -7 | -0.194 | -6.8 | -0.189 |
| ZINC11677168 | -7.2 | -0.2 | -7.7 | -0.214 | -7.2 | -0.2 | -6.8 | -0.189 |
| ZINC11677172 | -7.1 | -0.197 | -8 | -0.222 | -7 | -0.194 | -7.4 | -0.206 |
| ZINC11677178 | -8 | -0.222 | -7.9 | -0.219 | -8 | -0.222 | -7 | -0.194 |
| ZINC11681161 | -7.4 | -0.19 | -8 | -0.205 | -7.5 | -0.192 | -7.2 | -0.185 |
| ZINC11681164 | -7.4 | -0.19 | -8 | -0.205 | -7.4 | -0.19 | -7.3 | -0.187 |
| ZINC11681166 | -7.7 | -0.197 | -7.9 | -0.203 | -7.8 | -0.2 | -7.3 | -0.187 |
| ZINC11681170 | -7.4 | -0.19 | -7.8 | -0.2 | -7.4 | -0.19 | -7.4 | -0.19 |
| ZINC12153441 | -9.4 | -0.285 | -7.4 | -0.224 | -6.4 | -0.194 | -6.3 | -0.191 |
| ZINC12153442 | -8.3 | -0.252 | -7.4 | -0.224 | -6.7 | -0.203 | -6.6 | -0.2 |
| ZINC12340155 | -6.2 | -0.388 | -5.6 | -0.35 | -5.3 | -0.331 | -5.3 | -0.331 |
| ZINC12340238 | -7.3 | -0.429 | -6.2 | -0.365 | -5.9 | -0.347 | -5.8 | -0.341 |
| ZINC12341271 | -6.5 | -0.464 | -6.1 | -0.436 | -5.3 | -0.379 | -5.3 | -0.379 |
| ZINC12341321 | -6.3 | -0.485 | -5.4 | -0.415 | -5.4 | -0.415 | -5.4 | -0.415 |
| ZINC12341336 | -6.6 | -0.44 | -5.4 | -0.36 | -5.3 | -0.353 | -5.3 | -0.353 |
| ZINC12358863 | -8.8 | -0.463 | -7.1 | -0.374 | -6.6 | -0.347 | -6.2 | -0.326 |
| ZINC12363224 | -5.7 | -0.475 | -4.9 | -0.408 | -4.4 | -0.367 | -5.6 | -0.467 |
| ZINC12367936_1 | -6 | -0.286 | -7.2 | -0.343 | -6 | -0.286 | -5.9 | -0.281 |
| ZINC12367936 | -7.3 | -0.348 | -7.2 | -0.343 | -6 | -0.286 | -5.9 | -0.281 |
| ZINC12368248 | -8.4 | -0.4 | -6.9 | -0.329 | -6.6 | -0.314 | -6.3 | -0.3 |
| ZINC12368665 | -8.5 | -0.405 | -7.1 | -0.338 | -6.4 | -0.305 | -6.2 | -0.295 |
| ZINC12417345 | -6.4 | -0.457 | -5.5 | -0.393 | -4.9 | -0.35 | -5.3 | -0.379 |
| ZINC12504140 | -7.7 | -0.405 | -5.8 | -0.305 | -6.2 | -0.326 | -5.3 | -0.279 |
| ZINC12504141 | -7 | -0.368 | -6.1 | -0.321 | -5.4 | -0.284 | -5.3 | -0.279 |
| ZINC12504142 | -7.1 | -0.374 | -5.5 | -0.289 | -5.9 | -0.311 | -5.3 | -0.279 |
| ZINC12670903 | -7.7 | -0.241 | -7.4 | -0.231 | -6.8 | -0.212 | -7 | -0.219 |
| ZINC12670909 | -7.1 | -0.222 | -7.7 | -0.241 | -7.3 | -0.228 | -7 | -0.219 |
| ZINC12670914 | -7.2 | -0.225 | -7.9 | -0.247 | -7.5 | -0.234 | -7.1 | -0.222 |
| ZINC12670920 | -6.7 | -0.209 | -7.8 | -0.244 | -6.9 | -0.216 | -7 | -0.219 |
| ZINC12670933 | -7.7 | -0.453 | -5.8 | -0.341 | -5.8 | -0.341 | -5.6 | -0.329 |
| ZINC12671128 | -8 | -0.421 | -6.7 | -0.353 | -5.9 | -0.311 | -5.7 | -0.3 |
| ZINC12671501 | -7.8 | -0.433 | -5.9 | -0.328 | -6 | -0.333 | -5.4 | -0.3 |
| ZINC12671506 | -7.1 | -0.394 | -5.9 | -0.328 | -5.5 | -0.306 | -6.1 | -0.339 |
| ZINC12671886 | -7.2 | -0.24 | -8.6 | -0.287 | -7.7 | -0.257 | -7.1 | -0.237 |
| ZINC12671893 | -7.4 | -0.247 | -8.6 | -0.287 | -7.4 | -0.247 | -7.2 | -0.24 |
| ZINC12671898 | -7.1 | -0.237 | -8 | -0.267 | -7.2 | -0.24 | -7.2 | -0.24 |
| ZINC12671904 | -8.1 | -0.27 | -8.4 | -0.28 | -7.2 | -0.24 | -7.3 | -0.243 |
| ZINC12672222 | -8.7 | -0.3 | -7.2 | -0.248 | -6.5 | -0.224 | -6.2 | -0.214 |
| ZINC12672225 | -8 | -0.276 | -7.4 | -0.255 | -6.6 | -0.228 | -6 | -0.207 |
| ZINC12672231 | -7.6 | -0.262 | -7.1 | -0.245 | -6.6 | -0.228 | -6.6 | -0.228 |
| ZINC12672242_1 | -7.9 | -0.272 | -8.8 | -0.303 | -7.3 | -0.252 | -6.8 | -0.234 |
| ZINC12672242 | -8.2 | -0.283 | -8.8 | -0.303 | -7.3 | -0.252 | -7.9 | -0.272 |
| ZINC12672252_1 | -7.9 | -0.272 | -8.7 | -0.3 | -7.2 | -0.248 | -7.7 | -0.266 |
| ZINC12672252 | -8.2 | -0.283 | -8.7 | -0.3 | -7.2 | -0.248 | -7.3 | -0.252 |
| ZINC12959181 | -6.5 | -0.283 | -6.7 | -0.291 | -6 | -0.261 | -6 | -0.261 |
| ZINC13000556 | -7.2 | -0.232 | -7.4 | -0.239 | -7.2 | -0.232 | -7.1 | -0.229 |
| ZINC13042892 | -7.7 | -0.453 | -6.3 | -0.371 | -5.7 | -0.335 | -5.8 | -0.341 |
| ZINC13086327 | -6.8 | -0.486 | -5.2 | -0.371 | -4.9 | -0.35 | -5.7 | -0.407 |
| ZINC13099024 | -7 | -0.292 | -7.8 | -0.325 | -7.1 | -0.296 | -6.9 | -0.288 |
| ZINC13099025 | -7.4 | -0.308 | -8.3 | -0.346 | -7.2 | -0.3 | -7.2 | -0.3 |
| ZINC13099027 | -7.5 | -0.312 | -7.4 | -0.308 | -6.9 | -0.288 | -6.8 | -0.283 |
| ZINC13099047 | -7.4 | -0.231 | -8.3 | -0.259 | -7.3 | -0.228 | -7.4 | -0.231 |
| ZINC13099048 | -7.7 | -0.241 | -8.2 | -0.256 | -8.4 | -0.263 | -7.6 | -0.237 |
| ZINC13099050 | -7.5 | -0.234 | -8.6 | -0.269 | -7.5 | -0.234 | -6.9 | -0.216 |
| ZINC13099051 | -7.7 | -0.241 | -8.1 | -0.253 | -8.2 | -0.256 | -7.3 | -0.228 |
| ZINC13099284 | -7.9 | -0.316 | -7.1 | -0.284 | -6.8 | -0.272 | -5.9 | -0.236 |
| ZINC13099549_1 | -6.9 | -0.406 | -5.2 | -0.306 | -5.5 | -0.324 | -5.1 | -0.3 |
| ZINC13099549 | -7.1 | -0.418 | -5.1 | -0.3 | -5.5 | -0.324 | -5 | -0.294 |
| ZINC13099550_1 | -6.7 | -0.394 | -5 | -0.294 | -5.5 | -0.324 | -5.4 | -0.318 |
| ZINC13099550 | -6.8 | -0.4 | -4.9 | -0.288 | -5.4 | -0.318 | -5.2 | -0.306 |
| ZINC13099551_1 | -6.9 | -0.406 | -5 | -0.294 | -5.4 | -0.318 | -5.4 | -0.318 |
| ZINC13099551 | -6.8 | -0.4 | -5.2 | -0.306 | -5.4 | -0.318 | -5.1 | -0.3 |
| ZINC13110586 | -7 | -0.368 | -6.8 | -0.358 | -6.1 | -0.321 | -5.9 | -0.311 |
| ZINC13110587 | -7 | -0.412 | -6 | -0.353 | -5.5 | -0.324 | -5.5 | -0.324 |
| ZINC13115350 | -6.3 | -0.485 | -4.9 | -0.377 | -5.1 | -0.392 | -5.3 | -0.408 |
| ZINC13125763_1 | -6.6 | -0.388 | -6.2 | -0.365 | -6 | -0.353 | -6.1 | -0.359 |
| ZINC13125763 | -8.4 | -0.494 | -6.2 | -0.365 | -6.2 | -0.365 | -6.2 | -0.365 |
| ZINC13125795_1 | -8.7 | -0.458 | -7.2 | -0.379 | -6.6 | -0.347 | -6.2 | -0.326 |
| ZINC13125795 | -8.7 | -0.458 | -7.2 | -0.379 | -6.6 | -0.347 | -6.2 | -0.326 |
| ZINC13130011_1 | -8.7 | -0.458 | -6.6 | -0.347 | -6.6 | -0.347 | -6.1 | -0.321 |
| ZINC13130011 | -8.7 | -0.458 | -6.4 | -0.337 | -6.6 | -0.347 | -6 | -0.316 |
| ZINC13130015_1 | -6.9 | -0.46 | -5.9 | -0.393 | -5.7 | -0.38 | -5.8 | -0.387 |
| ZINC13130015 | -6.7 | -0.447 | -5.9 | -0.393 | -5.7 | -0.38 | -5.4 | -0.36 |
| ZINC13130016 | -8.4 | -0.467 | -7.1 | -0.394 | -6.3 | -0.35 | -6.2 | -0.344 |
| ZINC13130018 | -9 | -0.375 | -8.3 | -0.346 | -7.9 | -0.329 | -7.5 | -0.312 |
| ZINC13130035 | -7.6 | -0.422 | -5.9 | -0.328 | -5.8 | -0.322 | -6 | -0.333 |
| ZINC13130183 | -7.3 | -0.429 | -5.8 | -0.341 | -5.6 | -0.329 | -6 | -0.353 |
| ZINC13130187_1 | -9.1 | -0.314 | -7.6 | -0.262 | -7.7 | -0.266 | -7 | -0.241 |
| ZINC13130187 | -10.7 | -0.369 | -7.8 | -0.269 | -7.9 | -0.272 | -7.3 | -0.252 |
| ZINC13130211_1 | -7.7 | -0.335 | -6.6 | -0.287 | -6.5 | -0.283 | -5.8 | -0.252 |
| ZINC13130211 | -7.7 | -0.335 | -6.7 | -0.291 | -6.5 | -0.283 | -5.7 | -0.248 |
| ZINC13132551 | -7.6 | -0.475 | -6.5 | -0.406 | -6 | -0.375 | -6.5 | -0.406 |
| ZINC13136204 | -7.4 | -0.308 | -6.7 | -0.279 | -6.7 | -0.279 | -5.7 | -0.238 |
| ZINC13139292 | -5 | -0.455 | -4.3 | -0.391 | -4.3 | -0.391 | -4.3 | -0.391 |
| ZINC13139298 | -6.9 | -0.363 | -6.8 | -0.358 | -5.8 | -0.305 | -5.8 | -0.305 |
| ZINC13139306 | -7 | -0.368 | -6.8 | -0.358 | -5.5 | -0.289 | -5.5 | -0.289 |
| ZINC13139308_1 | -7 | -0.467 | -6.2 | -0.413 | -5.4 | -0.36 | -6.6 | -0.44 |
| ZINC13139308 | -7 | -0.467 | -6.2 | -0.413 | -5.3 | -0.353 | -6.1 | -0.407 |
| ZINC13139316 | -6.6 | -0.388 | -5 | -0.294 | -5 | -0.294 | -4.8 | -0.282 |
| ZINC13139317 | -6.5 | -0.295 | -5.6 | -0.255 | -5.3 | -0.241 | -4.3 | -0.195 |
| ZINC13139331 | -7.6 | -0.362 | -6.4 | -0.305 | -6.3 | -0.3 | -5.8 | -0.276 |
| ZINC13140104 | -6.1 | -0.436 | -5.1 | -0.364 | -5.2 | -0.371 | -5 | -0.357 |
| ZINC13140224 | -6.4 | -0.4 | -5.3 | -0.331 | -5 | -0.312 | -5.1 | -0.319 |
| ZINC13140225 | -6.3 | -0.42 | -5 | -0.333 | -4.8 | -0.32 | -4.5 | -0.3 |
| ZINC13140234_1 | -7 | -0.389 | -5.6 | -0.311 | -5.2 | -0.289 | -4.9 | -0.272 |
| ZINC13140234 | -7.2 | -0.4 | -5.5 | -0.306 | -5.2 | -0.289 | -4.9 | -0.272 |
| ZINC13140243 | -8.2 | -0.432 | -6.4 | -0.337 | -6.4 | -0.337 | -5.9 | -0.311 |
| ZINC13140245 | -7.7 | -0.405 | -6.6 | -0.347 | -6.5 | -0.342 | -5.8 | -0.305 |
| ZINC13140246 | -7.6 | -0.475 | -6.1 | -0.381 | -6.2 | -0.388 | -5.5 | -0.344 |
| ZINC13142971 | -8.3 | -0.437 | -5.5 | -0.289 | -5.4 | -0.284 | -6.1 | -0.321 |
| ZINC13142972 | -7.9 | -0.416 | -6.1 | -0.321 | -5.4 | -0.284 | -5.5 | -0.289 |
| ZINC13142989 | -7.3 | -0.317 | -7.2 | -0.313 | -6.9 | -0.3 | -7 | -0.304 |
| ZINC13142991 | -4.8 | -0.48 | -4 | -0.4 | -3.8 | -0.38 | -4.4 | -0.44 |
| ZINC13142993 | -8.4 | -0.336 | -7.3 | -0.292 | -7.2 | -0.288 | -6.8 | -0.272 |
| ZINC13143008 | -8.3 | -0.296 | -7.5 | -0.268 | -7.2 | -0.257 | -7.1 | -0.254 |
| ZINC13143009 | -8.3 | -0.296 | -7.3 | -0.261 | -7.2 | -0.257 | -7.1 | -0.254 |
| ZINC13143011 | -7.4 | -0.352 | -6.6 | -0.314 | -6.5 | -0.31 | -6.6 | -0.314 |
| ZINC13143017 | -6.3 | -0.263 | -5.9 | -0.246 | -5.4 | -0.225 | -5.4 | -0.225 |
| ZINC13143019 | -8.6 | -0.41 | -8.2 | -0.39 | -6.9 | -0.329 | -6.9 | -0.329 |
| ZINC13143021_1 | -6.4 | -0.457 | -5.7 | -0.407 | -5 | -0.357 | -5.6 | -0.4 |
| ZINC13143021 | -6.5 | -0.464 | -5.9 | -0.421 | -4.9 | -0.35 | -5 | -0.357 |
| ZINC13144115 | -7.4 | -0.336 | -5.8 | -0.264 | -5.7 | -0.259 | -5.7 | -0.259 |
| ZINC13144116 | -7.9 | -0.359 | -6.1 | -0.277 | -5.7 | -0.259 | -5.6 | -0.255 |
| ZINC13144542 | -7 | -0.292 | -7.2 | -0.3 | -7 | -0.292 | -6.9 | -0.288 |
| ZINC13144560_1 | -7.3 | -0.365 | -5.5 | -0.275 | -5.5 | -0.275 | -5.1 | -0.255 |
| ZINC13144560 | -7.5 | -0.375 | -5.6 | -0.28 | -5.5 | -0.275 | -4.9 | -0.245 |
| ZINC13144564 | -7.1 | -0.507 | -5.8 | -0.414 | -5.1 | -0.364 | -5.4 | -0.386 |
| ZINC13144566 | -6.8 | -0.425 | -5.6 | -0.35 | -5.2 | -0.325 | -5.2 | -0.325 |
| ZINC13144568 | -6.7 | -0.419 | -5.7 | -0.356 | -5.7 | -0.356 | -5.2 | -0.325 |
| ZINC13144613_1 | -4.9 | -0.49 | -4.2 | -0.42 | -4.1 | -0.41 | -4.7 | -0.47 |
| ZINC13144613 | -4.7 | -0.47 | -4.2 | -0.42 | -4 | -0.4 | -4.2 | -0.42 |
| ZINC13144614_1 | -6 | -0.333 | -5.1 | -0.283 | -5 | -0.278 | -4.6 | -0.256 |
| ZINC13144614 | -6.1 | -0.339 | -4.9 | -0.272 | -4.7 | -0.261 | -4.9 | -0.272 |
| ZINC13144616 | -6.1 | -0.407 | -5.5 | -0.367 | -5.2 | -0.347 | -5.8 | -0.387 |
| ZINC13144618 | -6.6 | -0.44 | -5.2 | -0.347 | -5.1 | -0.34 | -5.8 | -0.387 |
| ZINC13144621 | -7.9 | -0.439 | -6.5 | -0.361 | -6.3 | -0.35 | -5.9 | -0.328 |
| ZINC13145027 | -7.9 | -0.282 | -6.9 | -0.246 | -6.2 | -0.221 | -6.3 | -0.225 |
| ZINC13145028 | -7.8 | -0.279 | -7.2 | -0.257 | -6.2 | -0.221 | -6.2 | -0.221 |
| ZINC13145029 | -8.1 | -0.289 | -7.3 | -0.261 | -6.1 | -0.218 | -6.1 | -0.218 |
| ZINC13145030 | -7.3 | -0.261 | -6.9 | -0.246 | -6.2 | -0.221 | -6.1 | -0.218 |
| ZINC13146918 | -7.9 | -0.465 | -6.3 | -0.371 | -5.8 | -0.341 | -5.4 | -0.318 |
| ZINC13146942 | -7.3 | -0.429 | -5.8 | -0.341 | -5.7 | -0.335 | -5.6 | -0.329 |
| ZINC13146950 | -7.5 | -0.375 | -6.1 | -0.305 | -6.1 | -0.305 | -5.8 | -0.29 |
| ZINC13146954 | -7.1 | -0.507 | -6 | -0.429 | -5.6 | -0.4 | -6.3 | -0.45 |
| ZINC13146957 | -6.5 | -0.5 | -5.5 | -0.423 | -5.5 | -0.423 | -5.1 | -0.392 |
| ZINC13152217 | -7.1 | -0.355 | -6.7 | -0.335 | -6.1 | -0.305 | -6 | -0.3 |
| ZINC13152219 | -7.2 | -0.48 | -5.6 | -0.373 | -5.6 | -0.373 | -5.7 | -0.38 |
| ZINC13152221 | -7.4 | -0.463 | -5.5 | -0.344 | -5.4 | -0.338 | -5.2 | -0.325 |
| ZINC13152223 | -10.7 | -0.486 | -7 | -0.318 | -7.2 | -0.327 | -6.3 | -0.286 |
| ZINC13152225 | -8.5 | -0.405 | -7.2 | -0.343 | -7.2 | -0.343 | -6.6 | -0.314 |
| ZINC13152226 | -8.7 | -0.378 | -7.3 | -0.317 | -7 | -0.304 | -7 | -0.304 |
| ZINC13152237 | -6.8 | -0.425 | -5.5 | -0.344 | -5.4 | -0.338 | -5.4 | -0.338 |
| ZINC13152238 | -8.1 | -0.405 | -7.1 | -0.355 | -6.2 | -0.31 | -6.2 | -0.31 |
| ZINC13152247_1 | -7.9 | -0.316 | -6.8 | -0.272 | -6.1 | -0.244 | -6.5 | -0.26 |
| ZINC13152247 | -7.9 | -0.316 | -6.8 | -0.272 | -6.1 | -0.244 | -6.1 | -0.244 |
| ZINC13152248 | -6.1 | -0.508 | -5.4 | -0.45 | -5.3 | -0.442 | -5.2 | -0.433 |
| ZINC13152249_1 | -6.4 | -0.376 | -5.9 | -0.347 | -5 | -0.294 | -5.4 | -0.318 |
| ZINC13152249 | -7 | -0.412 | -5.9 | -0.347 | -4.9 | -0.288 | -5.3 | -0.312 |
| ZINC13152277 | -7.9 | -0.527 | -6 | -0.4 | -5.5 | -0.367 | -6 | -0.4 |
| ZINC13152278_1 | -7.7 | -0.453 | -6.2 | -0.365 | -5.6 | -0.329 | -5.6 | -0.329 |
| ZINC13152278 | -7.6 | -0.447 | -6.2 | -0.365 | -5.5 | -0.324 | -5.6 | -0.329 |
| ZINC13152279_1 | -7.6 | -0.447 | -6 | -0.353 | -5.5 | -0.324 | -5.5 | -0.324 |
| ZINC13152279 | -7.6 | -0.447 | -6 | -0.353 | -5.5 | -0.324 | -5.4 | -0.318 |
| ZINC13152282 | -6.8 | -0.425 | -4.9 | -0.306 | -5 | -0.312 | -4.9 | -0.306 |
| ZINC13152284 | -8.9 | -0.297 | -8.6 | -0.287 | -8 | -0.267 | -8.5 | -0.283 |
| ZINC13152291_1 | -8.2 | -0.39 | -6.3 | -0.3 | -6.4 | -0.305 | -5.8 | -0.276 |
| ZINC13152291 | -8.1 | -0.386 | -6.3 | -0.3 | -6.4 | -0.305 | -5.6 | -0.267 |
| ZINC13152292 | -6 | -0.462 | -4.8 | -0.369 | -5 | -0.385 | -5.1 | -0.392 |
| ZINC13152294 | -8.6 | -0.43 | -6.8 | -0.34 | -6.4 | -0.32 | -6.2 | -0.31 |
| ZINC13152611_1 | -6.9 | -0.329 | -5.9 | -0.281 | -5.5 | -0.262 | -5.3 | -0.252 |
| ZINC13152611 | -7.5 | -0.357 | -5.8 | -0.276 | -5.3 | -0.252 | -5 | -0.238 |
| ZINC13152612 | -7.1 | -0.323 | -6.5 | -0.295 | -5.9 | -0.268 | -6.8 | -0.309 |
| ZINC13152613 | -8.3 | -0.377 | -6.3 | -0.286 | -6.3 | -0.286 | -5.3 | -0.241 |
| ZINC13152614 | -7.4 | -0.336 | -6.3 | -0.286 | -5.9 | -0.268 | -6 | -0.273 |
| ZINC13152615 | -7.9 | -0.359 | -6.6 | -0.3 | -5.8 | -0.264 | -5.8 | -0.264 |
| ZINC13152616 | -6.6 | -0.44 | -5.3 | -0.353 | -5.2 | -0.347 | -6.2 | -0.413 |
| ZINC13154271 | -5.4 | -0.491 | -4.6 | -0.418 | -4.3 | -0.391 | -4.1 | -0.373 |
| ZINC13154273 | -7 | -0.333 | -6.2 | -0.295 | -5.8 | -0.276 | -5.7 | -0.271 |
| ZINC13154282 | -7.7 | -0.335 | -7.2 | -0.313 | -6.2 | -0.27 | -5.9 | -0.257 |
| ZINC13154298 | -7.8 | -0.312 | -7.2 | -0.288 | -6.9 | -0.276 | -6.8 | -0.272 |
| ZINC13154300 | -6.9 | -0.256 | -7.7 | -0.285 | -6.8 | -0.252 | -6.5 | -0.241 |
| ZINC13154302 | -7.8 | -0.289 | -7.9 | -0.293 | -6.8 | -0.252 | -6.6 | -0.244 |
| ZINC13154304 | -7.8 | -0.289 | -8.4 | -0.311 | -7.5 | -0.278 | -7.9 | -0.293 |
| ZINC13154305 | -7.3 | -0.27 | -8.6 | -0.319 | -7.8 | -0.289 | -7.3 | -0.27 |
| ZINC13154306 | -7 | -0.259 | -8.2 | -0.304 | -7.5 | -0.278 | -7.1 | -0.263 |
| ZINC13154307 | -7.9 | -0.293 | -8.4 | -0.311 | -7.1 | -0.263 | -6.8 | -0.252 |
| ZINC13154313 | -5.3 | -0.408 | -5 | -0.385 | -4.6 | -0.354 | -5.3 | -0.408 |
| ZINC13154314 | -6 | -0.462 | -5.2 | -0.4 | -4.9 | -0.377 | -4.9 | -0.377 |
| ZINC13154315 | -7.3 | -0.429 | -5.9 | -0.347 | -5.8 | -0.341 | -6 | -0.353 |
| ZINC13154317_1 | -5.9 | -0.454 | -5.1 | -0.392 | -4.9 | -0.377 | -5.5 | -0.423 |
| ZINC13154317 | -6.4 | -0.492 | -5 | -0.385 | -5.2 | -0.4 | -5.5 | -0.423 |
| ZINC13154319 | -6.5 | -0.271 | -6.9 | -0.288 | -6.5 | -0.271 | -6.1 | -0.254 |
| ZINC13154320 | -6.6 | -0.275 | -7.1 | -0.296 | -6.8 | -0.283 | -6.2 | -0.258 |
| ZINC13154321_1 | -6.6 | -0.388 | -6 | -0.353 | -5.1 | -0.3 | -5.2 | -0.306 |
| ZINC13154321 | -7.2 | -0.424 | -5.9 | -0.347 | -5.1 | -0.3 | -5.2 | -0.306 |
| ZINC13154324 | -6.6 | -0.33 | -7.4 | -0.37 | -6.1 | -0.305 | -5.5 | -0.275 |
| ZINC13154325 | -8 | -0.4 | -7.2 | -0.36 | -6 | -0.3 | -5.6 | -0.28 |
| ZINC13154326 | -5.7 | -0.438 | -5.3 | -0.408 | -5 | -0.385 | -5.1 | -0.392 |
| ZINC13154328 | -8.3 | -0.437 | -6.6 | -0.347 | -6.4 | -0.337 | -6.5 | -0.342 |
| ZINC13154329 | -8.2 | -0.432 | -6.7 | -0.353 | -6.3 | -0.332 | -6 | -0.316 |
| ZINC13207617_1 | -5.2 | -0.371 | -4.9 | -0.35 | -4.6 | -0.329 | -4.6 | -0.329 |
| ZINC13207617 | -5.4 | -0.386 | -5 | -0.357 | -4.6 | -0.329 | -4.6 | -0.329 |
| ZINC13208966 | -7.2 | -0.343 | -7.4 | -0.352 | -6.9 | -0.329 | -6.7 | -0.319 |
| ZINC13281671_1 | -7.5 | -0.536 | -6.2 | -0.443 | -5.5 | -0.393 | -5.8 | -0.414 |
| ZINC13281671 | -7.6 | -0.543 | -6.3 | -0.45 | -5.9 | -0.421 | -5.8 | -0.414 |
| ZINC13282591 | -6.2 | -0.477 | -5 | -0.385 | -5 | -0.385 | -4.9 | -0.377 |
| ZINC13284902_1 | -7.6 | -0.447 | -6.1 | -0.359 | -6 | -0.353 | -6.2 | -0.365 |
| ZINC13284902 | -7.5 | -0.441 | -5.9 | -0.347 | -5.8 | -0.341 | -6.1 | -0.359 |
| ZINC13451125 | -9 | -0.243 | -8.3 | -0.224 | -6.8 | -0.184 | -6.7 | -0.181 |
| ZINC13522091 | -7.1 | -0.245 | -7 | -0.241 | -7.4 | -0.255 | -7.4 | -0.255 |
| ZINC13542620_1 | -7.1 | -0.444 | -5.8 | -0.362 | -5.6 | -0.35 | -5.6 | -0.35 |
| ZINC13542620 | -7.2 | -0.45 | -5.7 | -0.356 | -5.7 | -0.356 | -4.9 | -0.306 |
| ZINC13542876 | -7.9 | -0.439 | -6.9 | -0.383 | -6.8 | -0.378 | -6.4 | -0.356 |
| ZINC13597216 | -5.3 | -0.53 | -4.4 | -0.44 | -4.7 | -0.47 | -4.6 | -0.46 |
| ZINC13597218 | -7.4 | -0.463 | -6 | -0.375 | -5.8 | -0.362 | -6.2 | -0.388 |
| ZINC13597219_1 | -7.3 | -0.406 | -6.1 | -0.339 | -6 | -0.333 | -5.5 | -0.306 |
| ZINC13597219 | -7.4 | -0.411 | -6.1 | -0.339 | -6 | -0.333 | -5.7 | -0.317 |
| ZINC13597222 | -6.2 | -0.413 | -4.6 | -0.307 | -5.1 | -0.34 | -4.9 | -0.327 |
| ZINC13597230_1 | -7.2 | -0.343 | -5.6 | -0.267 | -5.4 | -0.257 | -5.7 | -0.271 |
| ZINC13597230 | -7.1 | -0.338 | -5.6 | -0.267 | -5.2 | -0.248 | -5.3 | -0.252 |
| ZINC13597232 | -4.7 | -0.47 | -4.3 | -0.43 | -3.7 | -0.37 | -4.3 | -0.43 |
| ZINC13597237_1 | -5.9 | -0.454 | -4.7 | -0.362 | -4.8 | -0.369 | -4.6 | -0.354 |
| ZINC13597237 | -6 | -0.462 | -4.7 | -0.362 | -4.8 | -0.369 | -4.6 | -0.354 |
| ZINC13597273_1 | -7.5 | -0.441 | -5.9 | -0.347 | -5.7 | -0.335 | -5.4 | -0.318 |
| ZINC13597273 | -7.4 | -0.435 | -6.1 | -0.359 | -5.6 | -0.329 | -5.6 | -0.329 |
| ZINC13597276_1 | -8 | -0.471 | -6.6 | -0.388 | -5.8 | -0.341 | -5.7 | -0.335 |
| ZINC13597276 | -8 | -0.471 | -6.5 | -0.382 | -5.8 | -0.341 | -5.8 | -0.341 |
| ZINC13597278_1 | -7.7 | -0.453 | -6.4 | -0.376 | -5.3 | -0.312 | -5.8 | -0.341 |
| ZINC13597278 | -8 | -0.471 | -6.4 | -0.376 | -5.5 | -0.324 | -5.6 | -0.329 |
| ZINC13597281 | -5.9 | -0.454 | -5 | -0.385 | -4.8 | -0.369 | -4.6 | -0.354 |
| ZINC13597298 | -6.9 | -0.431 | -5.3 | -0.331 | -5.1 | -0.319 | -5.8 | -0.362 |
| ZINC13597334 | -7.5 | -0.341 | -6.1 | -0.277 | -5.6 | -0.255 | -5.6 | -0.255 |
| ZINC13597335 | -8.3 | -0.461 | -6.2 | -0.344 | -5.8 | -0.322 | -5.8 | -0.322 |
| ZINC13597337 | -6.1 | -0.469 | -5.1 | -0.392 | -5.1 | -0.392 | -5.2 | -0.4 |
| ZINC13597346 | -7.6 | -0.281 | -7.9 | -0.293 | -7 | -0.259 | -6.8 | -0.252 |
| ZINC13597348 | -8.4 | -0.311 | -7.5 | -0.278 | -7.2 | -0.267 | -7.3 | -0.27 |
| ZINC13597350 | -6.9 | -0.431 | -5.4 | -0.338 | -5.4 | -0.338 | -5.4 | -0.338 |
| ZINC13597354_1 | -7.9 | -0.439 | -5.9 | -0.328 | -5.7 | -0.317 | -6.2 | -0.344 |
| ZINC13597354 | -7.6 | -0.422 | -5.9 | -0.328 | -5.6 | -0.311 | -6.1 | -0.339 |
| ZINC13597363 | -6.1 | -0.555 | -5.2 | -0.473 | -5.6 | -0.509 | -5.6 | -0.509 |
| ZINC13597368 | -6.9 | -0.246 | -7.6 | -0.271 | -6.8 | -0.243 | -6.2 | -0.221 |
| ZINC13597372 | -7 | -0.467 | -5.6 | -0.373 | -5.4 | -0.36 | -5 | -0.333 |
| ZINC13597383 | -6.7 | -0.372 | -5.5 | -0.306 | -5 | -0.278 | -4.9 | -0.272 |
| ZINC13597390 | -6 | -0.5 | -4.8 | -0.4 | -4.5 | -0.375 | -4.5 | -0.375 |
| ZINC13597410 | -7.2 | -0.288 | -7.7 | -0.308 | -6.7 | -0.268 | -6.7 | -0.268 |
| ZINC13597416_1 | -8.4 | -0.494 | -6.2 | -0.365 | -6.2 | -0.365 | -6.1 | -0.359 |
| ZINC13597416 | -8.4 | -0.494 | -6.2 | -0.365 | -6.2 | -0.365 | -6.1 | -0.359 |
| ZINC13597418 | -7.8 | -0.433 | -6.5 | -0.361 | -6 | -0.333 | -5.6 | -0.311 |
| ZINC13597424 | -8.1 | -0.405 | -6 | -0.3 | -5.2 | -0.26 | -5.7 | -0.285 |
| ZINC13597426 | -7.8 | -0.39 | -6.1 | -0.305 | -5.8 | -0.29 | -5.5 | -0.275 |
| ZINC13597428_1 | -6.1 | -0.436 | -5.3 | -0.379 | -4.6 | -0.329 | -4.5 | -0.321 |
| ZINC13597428 | -6 | -0.429 | -5 | -0.357 | -4.8 | -0.343 | -4.6 | -0.329 |
| ZINC13597430_1 | -6.9 | -0.46 | -5 | -0.333 | -5.2 | -0.347 | -5.4 | -0.36 |
| ZINC13597430 | -6.4 | -0.427 | -5.1 | -0.34 | -5.2 | -0.347 | -5.2 | -0.347 |
| ZINC13597432 | -8.8 | -0.352 | -7.4 | -0.296 | -7 | -0.28 | -6.6 | -0.264 |
| ZINC13597717 | -6.6 | -0.44 | -5.2 | -0.347 | -5.1 | -0.34 | -5.7 | -0.38 |
| ZINC13597721 | -6.4 | -0.305 | -7 | -0.333 | -6.4 | -0.305 | -6.4 | -0.305 |
| ZINC13597728 | -8.6 | -0.478 | -6.1 | -0.339 | -5.6 | -0.311 | -5.7 | -0.317 |
| ZINC13597730_1 | -8.2 | -0.39 | -6.6 | -0.314 | -6.8 | -0.324 | -6.5 | -0.31 |
| ZINC13597730 | -8.2 | -0.39 | -7 | -0.333 | -6.7 | -0.319 | -6.6 | -0.314 |
| ZINC13597732_1 | -8.9 | -0.356 | -6 | -0.24 | -5.9 | -0.236 | -6.3 | -0.252 |
| ZINC13597732 | -8.3 | -0.332 | -5.9 | -0.236 | -6.1 | -0.244 | -6.8 | -0.272 |
| ZINC13597738_1 | -6.3 | -0.332 | -6.4 | -0.337 | -5.9 | -0.311 | -6.3 | -0.332 |
| ZINC13597738 | -6.8 | -0.358 | -6.4 | -0.337 | -5.9 | -0.311 | -5.9 | -0.311 |
| ZINC13597741 | -6.1 | -0.469 | -5.4 | -0.415 | -4.8 | -0.369 | -5.5 | -0.423 |
| ZINC13597743 | -6.5 | -0.342 | -5.5 | -0.289 | -5.8 | -0.305 | -6 | -0.316 |
| ZINC13597746 | -9 | -0.409 | -6.7 | -0.305 | -6.5 | -0.295 | -6.1 | -0.277 |
| ZINC13597758 | -7.6 | -0.447 | -6.1 | -0.359 | -6 | -0.353 | -6 | -0.353 |
| ZINC13597762 | -7.7 | -0.428 | -5.9 | -0.328 | -5.8 | -0.322 | -5.4 | -0.3 |
| ZINC13597764 | -6.8 | -0.378 | -5.8 | -0.322 | -5.3 | -0.294 | -5 | -0.278 |
| ZINC13597767 | -8.6 | -0.41 | -6.7 | -0.319 | -6.7 | -0.319 | -6.9 | -0.329 |
| ZINC13597771 | -6.5 | -0.382 | -5.7 | -0.335 | -5.7 | -0.335 | -5.7 | -0.335 |
| ZINC13597814 | -6 | -0.286 | -6.7 | -0.319 | -6.3 | -0.3 | -5.9 | -0.281 |
| ZINC13597816 | -6.8 | -0.324 | -6.7 | -0.319 | -6.1 | -0.29 | -5.9 | -0.281 |
| ZINC13597818 | -7.8 | -0.371 | -7 | -0.333 | -6 | -0.286 | -5.9 | -0.281 |
| ZINC13634510 | -4.3 | -0.478 | -4.2 | -0.467 | -3.5 | -0.389 | -4.4 | -0.489 |
| ZINC13634517 | -7.8 | -0.459 | -6.1 | -0.359 | -6.2 | -0.365 | -5.5 | -0.324 |
| ZINC13634519 | -7.9 | -0.439 | -6.1 | -0.339 | -6.5 | -0.361 | -5.6 | -0.311 |
| ZINC14717925 | -7.6 | -0.292 | -8.8 | -0.338 | -6.9 | -0.265 | -6.9 | -0.265 |
| ZINC15881824_1 | -4.9 | -0.49 | -4.3 | -0.43 | -4.1 | -0.41 | -4.6 | -0.46 |
| ZINC15881824 | -5.1 | -0.51 | -4.4 | -0.44 | -4.1 | -0.41 | -4.7 | -0.47 |
| ZINC15889046 | -6.1 | -0.555 | -5.3 | -0.482 | -5 | -0.455 | -5.9 | -0.536 |
| ZINC15894745 | -7.1 | -0.394 | -5.9 | -0.328 | -5.7 | -0.317 | -5.6 | -0.311 |
| ZINC15924502 | -7 | -0.25 | -7.7 | -0.275 | -6.8 | -0.243 | -6.4 | -0.229 |
| ZINC15924534 | -7.5 | -0.469 | -6 | -0.375 | -5.7 | -0.356 | -5.6 | -0.35 |
| ZINC15924536 | -7.7 | -0.481 | -5.8 | -0.362 | -5.6 | -0.35 | -5.7 | -0.356 |
| ZINC15924541_1 | -7.7 | -0.385 | -6.2 | -0.31 | -6 | -0.3 | -5.9 | -0.295 |
| ZINC15924541 | -8 | -0.4 | -6 | -0.3 | -5.8 | -0.29 | -5.9 | -0.295 |
| ZINC15952559_1 | -9.1 | -0.455 | -6.9 | -0.345 | -6.7 | -0.335 | -6.6 | -0.33 |
| ZINC15952559 | -8.7 | -0.435 | -7.1 | -0.355 | -6.6 | -0.33 | -6.4 | -0.32 |
| ZINC15952610 | -7.6 | -0.507 | -5.4 | -0.36 | -5.6 | -0.373 | -5.6 | -0.373 |
| ZINC15990220_1 | -7.7 | -0.428 | -5.5 | -0.306 | -5.5 | -0.306 | -5.7 | -0.317 |
| ZINC15990220 | -7.7 | -0.428 | -5.8 | -0.322 | -5.6 | -0.311 | -5.7 | -0.317 |
| ZINC15990245_1 | -8 | -0.471 | -5.7 | -0.335 | -5.8 | -0.341 | -5.7 | -0.335 |
| ZINC15990245 | -8 | -0.471 | -5.7 | -0.335 | -5.8 | -0.341 | -5.8 | -0.341 |
| ZINC15990251_1 | -7.8 | -0.371 | -6.1 | -0.29 | -5.8 | -0.276 | -5.8 | -0.276 |
| ZINC15990251 | -7.8 | -0.371 | -6 | -0.286 | -5.7 | -0.271 | -5.7 | -0.271 |
| ZINC16051261 | -7.1 | -0.418 | -5.6 | -0.329 | -5.8 | -0.341 | -5.8 | -0.341 |
| ZINC16920429_1 | -8.7 | -0.512 | -6.3 | -0.371 | -6.1 | -0.359 | -6 | -0.353 |
| ZINC16920429 | -8.4 | -0.494 | -6.3 | -0.371 | -6.1 | -0.359 | -6.3 | -0.371 |
| ZINC16941278 | -6.2 | -0.443 | -5 | -0.357 | -4.9 | -0.35 | -4.5 | -0.321 |
| ZINC16948978 | -7.8 | -0.355 | -6.7 | -0.305 | -6.7 | -0.305 | -6.7 | -0.305 |
| ZINC16951318 | -8.1 | -0.289 | -6.4 | -0.229 | -5.8 | -0.207 | -5.7 | -0.204 |
| ZINC16951320 | -8.5 | -0.354 | -7.4 | -0.308 | -7.1 | -0.296 | -7 | -0.292 |
| ZINC16954225 | -8.7 | -0.483 | -6.3 | -0.35 | -6 | -0.333 | -5.6 | -0.311 |
| ZINC16969114 | -5.8 | -0.446 | -4.8 | -0.369 | -4.3 | -0.331 | -4.7 | -0.362 |
| ZINC16978136 | -6.7 | -0.419 | -6.1 | -0.381 | -5.5 | -0.344 | -5.1 | -0.319 |
| ZINC17105710 | -6.6 | -0.508 | -5.2 | -0.4 | -5.3 | -0.408 | -5.8 | -0.446 |
| ZINC17108282 | -6.9 | -0.46 | -5.2 | -0.347 | -5.5 | -0.367 | -5 | -0.333 |
| ZINC17125976 | -7.9 | -0.343 | -6.7 | -0.291 | -6.1 | -0.265 | -5.7 | -0.248 |
| ZINC17147424 | -7.2 | -0.288 | -8.1 | -0.324 | -7.2 | -0.288 | -6.5 | -0.26 |
| ZINC17147426 | -6.6 | -0.264 | -7.4 | -0.296 | -6.4 | -0.256 | -6.2 | -0.248 |
| ZINC17147429 | -7.4 | -0.296 | -7.5 | -0.3 | -6.8 | -0.272 | -6.4 | -0.256 |
| ZINC17147431 | -7.5 | -0.3 | -7.8 | -0.312 | -6.9 | -0.276 | -6.3 | -0.252 |
| ZINC17149563 | -7 | -0.333 | -7.4 | -0.352 | -6.4 | -0.305 | -6.4 | -0.305 |
| ZINC17149566 | -8.4 | -0.4 | -7.2 | -0.343 | -6.7 | -0.319 | -6.6 | -0.314 |
| ZINC17149568 | -7.9 | -0.376 | -7.4 | -0.352 | -6.5 | -0.31 | -6.4 | -0.305 |
| ZINC17149570 | -6.9 | -0.329 | -7.6 | -0.362 | -6.3 | -0.3 | -6.3 | -0.3 |
| ZINC17285513 | -6.3 | -0.525 | -5.6 | -0.467 | -5.5 | -0.458 | -6.1 | -0.508 |
| ZINC17287324 | -7.4 | -0.19 | -7.6 | -0.195 | -7 | -0.179 | -7 | -0.179 |
| ZINC17287328 | -8 | -0.205 | -7.3 | -0.187 | -6.8 | -0.174 | -7.1 | -0.182 |
| ZINC17287332 | -7.4 | -0.19 | -7.8 | -0.2 | -6.5 | -0.167 | -6.4 | -0.164 |
| ZINC17287336 | -7.7 | -0.197 | -7.6 | -0.195 | -6.8 | -0.174 | -7 | -0.179 |
| ZINC17303059 | -9.2 | -0.46 | -7.2 | -0.36 | -6.7 | -0.335 | -6.6 | -0.33 |
| ZINC17353911 | -7.8 | -0.223 | -8 | -0.229 | -7.3 | -0.209 | -7.4 | -0.211 |
| ZINC17353912 | -7.7 | -0.22 | -7.9 | -0.226 | -7.2 | -0.206 | -7.3 | -0.209 |
| ZINC17353913 | -7.9 | -0.226 | -7.6 | -0.217 | -7.6 | -0.217 | -7.5 | -0.214 |
| ZINC17353914 | -8 | -0.229 | -7.4 | -0.211 | -7.7 | -0.22 | -7.5 | -0.214 |
| ZINC17375647 | -7.2 | -0.277 | -8.1 | -0.312 | -6.7 | -0.258 | -6.6 | -0.254 |
| ZINC17375649 | -7.4 | -0.285 | -7.5 | -0.288 | -7.1 | -0.273 | -6.6 | -0.254 |
| ZINC17375651 | -7.4 | -0.285 | -7.6 | -0.292 | -7.3 | -0.281 | -6.9 | -0.265 |
| ZINC17375653 | -7.6 | -0.292 | -7.5 | -0.288 | -7.2 | -0.277 | -6.8 | -0.262 |
| ZINC17379198 | -6.8 | -0.324 | -5.7 | -0.271 | -5.4 | -0.257 | -5 | -0.238 |
| ZINC17419163 | -5.1 | -0.51 | -4.2 | -0.42 | -4.1 | -0.41 | -4.4 | -0.44 |
| ZINC17424892_1 | -8 | -0.4 | -7.2 | -0.36 | -7.1 | -0.355 | -6.2 | -0.31 |
| ZINC17424892 | -8.2 | -0.41 | -6.9 | -0.345 | -6.9 | -0.345 | -6.2 | -0.31 |
| ZINC17465817 | -7.8 | -0.325 | -5.9 | -0.246 | -6.5 | -0.271 | -5.3 | -0.221 |
| ZINC17465824 | -6.1 | -0.226 | -5.1 | -0.189 | -4.2 | -0.156 | -4.2 | -0.156 |
| ZINC17465958 | -8 | -0.286 | -8.1 | -0.289 | -7.3 | -0.261 | -7.1 | -0.254 |
| ZINC17465965 | -8 | -0.25 | -7.4 | -0.231 | -6.9 | -0.216 | -7 | -0.219 |
| ZINC17465972 | -8.7 | -0.362 | -7 | -0.292 | -6.9 | -0.288 | -6.9 | -0.288 |
| ZINC17465979 | -8.4 | -0.21 | -8.4 | -0.21 | -6.9 | -0.173 | -6.9 | -0.173 |
| ZINC17465983 | -8.4 | -0.21 | -8.4 | -0.21 | -7 | -0.175 | -7 | -0.175 |
| ZINC17529946_1 | -5.9 | -0.311 | -5.4 | -0.284 | -5.2 | -0.274 | -5.8 | -0.305 |
| ZINC17529946 | -6.5 | -0.342 | -5.9 | -0.311 | -5 | -0.263 | -5 | -0.263 |
| ZINC17729070 | -7.3 | -0.348 | -5.7 | -0.271 | -6 | -0.286 | -5.5 | -0.262 |
| ZINC17820077 | -6 | -0.429 | -5.4 | -0.386 | -4.8 | -0.343 | -4.9 | -0.35 |
| ZINC17858074_1 | -8.9 | -0.405 | -6.3 | -0.286 | -6.8 | -0.309 | -6.3 | -0.286 |
| ZINC17858074 | -8.8 | -0.4 | -6.5 | -0.295 | -6.6 | -0.3 | -6.4 | -0.291 |
| ZINC17860685 | -8.5 | -0.354 | -7.3 | -0.304 | -6.9 | -0.288 | -6.9 | -0.288 |
| ZINC17861701 | -6.4 | -0.427 | -4.9 | -0.327 | -4.6 | -0.307 | -4.6 | -0.307 |
| ZINC17878196 | -6.6 | -0.471 | -6 | -0.429 | -5.4 | -0.386 | -5.5 | -0.393 |
| ZINC17885079 | -7.2 | -0.424 | -6.4 | -0.376 | -5.8 | -0.341 | -5.6 | -0.329 |
| ZINC17886718 | -7.8 | -0.459 | -5.6 | -0.329 | -5.4 | -0.318 | -5.9 | -0.347 |
| ZINC17949075_1 | -8.1 | -0.3 | -7.1 | -0.263 | -7 | -0.259 | -6.9 | -0.256 |
| ZINC17949075 | -8 | -0.296 | -7.2 | -0.267 | -7.1 | -0.263 | -7.1 | -0.263 |
| ZINC17968970 | -9.1 | -0.337 | -6.8 | -0.252 | -6.8 | -0.252 | -6.3 | -0.233 |
| ZINC17970262_1 | -7.6 | -0.447 | -6.1 | -0.359 | -6.5 | -0.382 | -5.9 | -0.347 |
| ZINC17970262 | -7.6 | -0.447 | -6.1 | -0.359 | -6.5 | -0.382 | -6 | -0.353 |
| ZINC17995347_1 | -9.2 | -0.317 | -8 | -0.276 | -8 | -0.276 | -7.4 | -0.255 |
| ZINC17995347 | -8.7 | -0.3 | -7.9 | -0.272 | -8.2 | -0.283 | -7.5 | -0.259 |
| ZINC18006265 | -8.4 | -0.42 | -6.2 | -0.31 | -6.1 | -0.305 | -6.3 | -0.315 |
| ZINC18010927 | -7.6 | -0.4 | -5.9 | -0.311 | -5.9 | -0.311 | -5.3 | -0.279 |
| ZINC18030775 | -7.8 | -0.371 | -6.8 | -0.324 | -6.5 | -0.31 | -6.4 | -0.305 |
| ZINC18038389 | -8.7 | -0.348 | -6.8 | -0.272 | -6.7 | -0.268 | -6.6 | -0.264 |
| ZINC18043993 | -8.2 | -0.432 | -6.7 | -0.353 | -6.6 | -0.347 | -6.4 | -0.337 |
| ZINC18055497 | -8.5 | -0.283 | -8.7 | -0.29 | -7.9 | -0.263 | -8 | -0.267 |
| ZINC18056732 | -8.5 | -0.425 | -6.4 | -0.32 | -6.3 | -0.315 | -6.3 | -0.315 |
| ZINC18056840_1 | -9.1 | -0.455 | -7.2 | -0.36 | -7.1 | -0.355 | -6.7 | -0.335 |
| ZINC18056840 | -8.8 | -0.44 | -6.8 | -0.34 | -6.9 | -0.345 | -6.7 | -0.335 |
| ZINC18057104 | -7.4 | -0.195 | -7.8 | -0.205 | -8 | -0.211 | -7.9 | -0.208 |
| ZINC18057933 | -6.8 | -0.234 | -6.7 | -0.231 | -5.7 | -0.197 | -6.1 | -0.21 |
| ZINC18061739 | -7.8 | -0.312 | -6.8 | -0.272 | -6.6 | -0.264 | -6.4 | -0.256 |
| ZINC18067025 | -8.2 | -0.39 | -6.1 | -0.29 | -5.9 | -0.281 | -6.2 | -0.295 |
| ZINC18068040 | -7.6 | -0.38 | -6.6 | -0.33 | -6.4 | -0.32 | -5.8 | -0.29 |
| ZINC18068788 | -7.4 | -0.322 | -6.7 | -0.291 | -6.4 | -0.278 | -5.9 | -0.257 |
| ZINC18083898_1 | -6.5 | -0.433 | -5.1 | -0.34 | -5.4 | -0.36 | -5.6 | -0.373 |
| ZINC18083898 | -6.6 | -0.44 | -5.4 | -0.36 | -5.3 | -0.353 | -5.4 | -0.36 |
| ZINC18085106_1 | -8.2 | -0.373 | -7 | -0.318 | -7 | -0.318 | -6 | -0.273 |
| ZINC18085106 | -7.9 | -0.359 | -7 | -0.318 | -6.8 | -0.309 | -6.6 | -0.3 |
| ZINC18098114 | -8.3 | -0.307 | -7.3 | -0.27 | -8.2 | -0.304 | -6.9 | -0.256 |
| ZINC18098743 | -7.9 | -0.272 | -6.7 | -0.231 | -6.7 | -0.231 | -6.5 | -0.224 |
| ZINC18107657 | -7.1 | -0.355 | -5.7 | -0.285 | -5.5 | -0.275 | -5.4 | -0.27 |
| ZINC18117772 | -7.6 | -0.507 | -5.8 | -0.387 | -5.8 | -0.387 | -5.9 | -0.393 |
| ZINC18121761 | -6.5 | -0.382 | -5.9 | -0.347 | -5.6 | -0.329 | -5.6 | -0.329 |
| ZINC18124911_1 | -7 | -0.226 | -7.4 | -0.239 | -7 | -0.226 | -6.8 | -0.219 |
| ZINC18124911 | -6.7 | -0.216 | -7.6 | -0.245 | -6.9 | -0.223 | -7 | -0.226 |
| ZINC18126453 | -8.2 | -0.357 | -6.6 | -0.287 | -6.2 | -0.27 | -6.4 | -0.278 |
| ZINC18141142 | -7.5 | -0.417 | -5.3 | -0.294 | -5.3 | -0.294 | -5.4 | -0.3 |
| ZINC18141294 | -9 | -0.265 | -8.5 | -0.25 | -7.8 | -0.229 | -7.6 | -0.224 |
| ZINC18142874_1 | -7.9 | -0.416 | -6.2 | -0.326 | -6.2 | -0.326 | -5.8 | -0.305 |
| ZINC18142874 | -8.3 | -0.437 | -6.2 | -0.326 | -6.2 | -0.326 | -5.8 | -0.305 |
| ZINC18153859 | -7.7 | -0.453 | -5.9 | -0.347 | -5.9 | -0.347 | -5.5 | -0.324 |
| ZINC18154478 | -9.2 | -0.4 | -7.4 | -0.322 | -7.1 | -0.309 | -6.9 | -0.3 |
| ZINC18156788_1 | -8.6 | -0.453 | -7.3 | -0.384 | -7.3 | -0.384 | -6.1 | -0.321 |
| ZINC18156788 | -8.8 | -0.463 | -7 | -0.368 | -7 | -0.368 | -6.2 | -0.326 |
| ZINC18157167 | -7.5 | -0.25 | -7.5 | -0.25 | -7 | -0.233 | -7 | -0.233 |
| ZINC18163183 | -7.1 | -0.418 | -6.2 | -0.365 | -6.2 | -0.365 | -6 | -0.353 |
| ZINC18163421_1 | -7.6 | -0.38 | -7.1 | -0.355 | -6.8 | -0.34 | -6.5 | -0.325 |
| ZINC18163421 | -7.8 | -0.39 | -6.9 | -0.345 | -6.6 | -0.33 | -6.2 | -0.31 |
| ZINC18166466 | -7.5 | -0.441 | -6.4 | -0.376 | -6 | -0.353 | -6 | -0.353 |
| ZINC18166590 | -9 | -0.429 | -6.2 | -0.295 | -5.9 | -0.281 | -5.4 | -0.257 |
| ZINC18168901 | -4.9 | -0.613 | -4 | -0.5 | -4.3 | -0.537 | -4.4 | -0.55 |
| ZINC18169477 | -9.2 | -0.287 | -8.1 | -0.253 | -7 | -0.219 | -7.2 | -0.225 |
| ZINC18173699 | -8.3 | -0.437 | -6.5 | -0.342 | -6.1 | -0.321 | -6.3 | -0.332 |
| ZINC18178799 | -6.9 | -0.46 | -5.9 | -0.393 | -5.9 | -0.393 | -5.5 | -0.367 |
| ZINC18183236 | -7.7 | -0.405 | -5.7 | -0.3 | -5.6 | -0.295 | -6.4 | -0.337 |
| ZINC18189380_1 | -7.6 | -0.38 | -6.9 | -0.345 | -6.6 | -0.33 | -6.6 | -0.33 |
| ZINC18189380 | -7.9 | -0.395 | -6.6 | -0.33 | -5.8 | -0.29 | -6.1 | -0.305 |
| ZINC18192390 | -7.8 | -0.355 | -6.1 | -0.277 | -6 | -0.273 | -6.2 | -0.282 |
| ZINC18193021 | -6.8 | -0.486 | -5.2 | -0.371 | -5.1 | -0.364 | -5.1 | -0.364 |
| ZINC18193844_1 | -8.1 | -0.405 | -6.5 | -0.325 | -5.7 | -0.285 | -5.6 | -0.28 |
| ZINC18193844 | -7.6 | -0.38 | -6.3 | -0.315 | -5.8 | -0.29 | -5.5 | -0.275 |
| ZINC18207613 | -7.4 | -0.336 | -6.2 | -0.282 | -6.3 | -0.286 | -5.6 | -0.255 |
| ZINC18211623 | -8.3 | -0.377 | -6.6 | -0.3 | -6.3 | -0.286 | -6.2 | -0.282 |
| ZINC18212500 | -5.6 | -0.509 | -4.9 | -0.445 | -4.9 | -0.445 | -5 | -0.455 |
| ZINC18213583 | -7.5 | -0.268 | -7.3 | -0.261 | -6.7 | -0.239 | -6.5 | -0.232 |
| ZINC18217586 | -6.4 | -0.533 | -5.3 | -0.442 | -4.9 | -0.408 | -4.9 | -0.408 |
| ZINC18219562 | -7.8 | -0.339 | -6.8 | -0.296 | -6.8 | -0.296 | -6.2 | -0.27 |
| ZINC18219564 | -8.6 | -0.374 | -6.8 | -0.296 | -6.6 | -0.287 | -6.2 | -0.27 |
| ZINC18227443 | -5.8 | -0.527 | -4.5 | -0.409 | -4.6 | -0.418 | -5 | -0.455 |
| ZINC18825330 | -7.9 | -0.395 | -6.3 | -0.315 | -7 | -0.35 | -5.8 | -0.29 |
| ZINC18847035 | -7.1 | -0.473 | -6.8 | -0.453 | -5.6 | -0.373 | -5.6 | -0.373 |
| ZINC19205460 | -6.7 | -0.372 | -5.7 | -0.317 | -5.4 | -0.3 | -4.9 | -0.272 |
| ZINC19230120 | -4.4 | -0.489 | -3.9 | -0.433 | -3.6 | -0.4 | -3.9 | -0.433 |
| ZINC19322683 | -5.5 | -0.393 | -5.2 | -0.371 | -4.8 | -0.343 | -5.1 | -0.364 |
| ZINC19325788 | -6.6 | -0.508 | -5.2 | -0.4 | -5.2 | -0.4 | -5.1 | -0.392 |
| ZINC19325791 | -6.4 | -0.492 | -5.8 | -0.446 | -4.8 | -0.369 | -5.2 | -0.4 |
| ZINC19325794 | -5.9 | -0.454 | -5.3 | -0.408 | -5.2 | -0.4 | -5.6 | -0.431 |
| ZINC19362650_1 | -6.7 | -0.279 | -6.4 | -0.267 | -5.9 | -0.246 | -6 | -0.25 |
| ZINC19362650 | -5.8 | -0.242 | -6.6 | -0.275 | -6 | -0.25 | -5.9 | -0.246 |
| ZINC19362651_1 | -6 | -0.25 | -6.7 | -0.279 | -6.1 | -0.254 | -6 | -0.25 |
| ZINC19362651 | -5.9 | -0.246 | -6.9 | -0.288 | -6.1 | -0.254 | -6 | -0.25 |
| ZINC19366110 | -5.5 | -0.5 | -5.1 | -0.464 | -4.8 | -0.436 | -5.4 | -0.491 |
| ZINC19366557_1 | -4.9 | -0.49 | -4.1 | -0.41 | -3.9 | -0.39 | -4.3 | -0.43 |
| ZINC19366557 | -4.7 | -0.47 | -4.1 | -0.41 | -3.7 | -0.37 | -4.3 | -0.43 |
| ZINC19456377 | -6.8 | -0.283 | -5.8 | -0.242 | -5.4 | -0.225 | -5.1 | -0.212 |
| ZINC19456379 | -6.8 | -0.283 | -5.8 | -0.242 | -5.5 | -0.229 | -5.4 | -0.225 |
| ZINC19632726 | -7.1 | -0.263 | -7.1 | -0.263 | -7.1 | -0.263 | -6.4 | -0.237 |
| ZINC19701771_1 | -7.8 | -0.252 | -7.9 | -0.255 | -7.8 | -0.252 | -7 | -0.226 |
| ZINC19701771 | -7.7 | -0.248 | -7.9 | -0.255 | -7.7 | -0.248 | -7 | -0.226 |
| ZINC19735376 | -5.7 | -0.518 | -4.9 | -0.445 | -4.7 | -0.427 | -5.5 | -0.5 |
| ZINC19794764 | -7.7 | -0.308 | -6.1 | -0.244 | -6.6 | -0.264 | -6.1 | -0.244 |
| ZINC19794765 | -7.1 | -0.284 | -6.3 | -0.252 | -6.3 | -0.252 | -6.2 | -0.248 |
| ZINC19799271 | -5.9 | -0.454 | -5.1 | -0.392 | -5 | -0.385 | -5.6 | -0.431 |
| ZINC19909409_1 | -6.2 | -0.388 | -6.7 | -0.419 | -6 | -0.375 | -5.6 | -0.35 |
| ZINC19909409 | -6.4 | -0.4 | -6.7 | -0.419 | -6 | -0.375 | -5.6 | -0.35 |
| ZINC19923695 | -7.4 | -0.296 | -6.6 | -0.264 | -6.1 | -0.244 | -5.8 | -0.232 |
| ZINC19923697 | -8.3 | -0.332 | -6.4 | -0.256 | -6.3 | -0.252 | -6.2 | -0.248 |
| ZINC20028475_1 | -6.9 | -0.46 | -5.5 | -0.367 | -5.4 | -0.36 | -5.2 | -0.347 |
| ZINC20028475 | -7 | -0.467 | -5.5 | -0.367 | -5.4 | -0.36 | -5.2 | -0.347 |
| ZINC20421310 | -6.2 | -0.413 | -5.1 | -0.34 | -4.9 | -0.327 | -5.6 | -0.373 |
| ZINC21673561 | -7.4 | -0.352 | -5.8 | -0.276 | -5.8 | -0.276 | -5.8 | -0.276 |
| ZINC22267016 | -6.3 | -0.252 | -6.9 | -0.276 | -6.7 | -0.268 | -6.7 | -0.268 |
| ZINC22586514 | -4.4 | -0.44 | -3.9 | -0.39 | -3.6 | -0.36 | -4.3 | -0.43 |
| ZINC22910159 | -7.8 | -0.312 | -7.1 | -0.284 | -6.1 | -0.244 | -6.4 | -0.256 |
| ZINC22910179 | -6.8 | -0.378 | -5.7 | -0.317 | -5.5 | -0.306 | -5.3 | -0.294 |
| ZINC22910859 | -9.3 | -0.443 | -6.9 | -0.329 | -6.6 | -0.314 | -6.2 | -0.295 |
| ZINC22910942 | -7.9 | -0.395 | -6.4 | -0.32 | -6.3 | -0.315 | -6.3 | -0.315 |
| ZINC22911751 | -7.1 | -0.355 | -6 | -0.3 | -5.4 | -0.27 | -5.4 | -0.27 |
| ZINC22911959 | -8.8 | -0.338 | -7.5 | -0.288 | -6.9 | -0.265 | -6.1 | -0.235 |
| ZINC22912001 | -7.3 | -0.332 | -6.8 | -0.309 | -6.2 | -0.282 | -6.7 | -0.305 |
| ZINC22912013 | -8.5 | -0.405 | -7.1 | -0.338 | -7.1 | -0.338 | -6.6 | -0.314 |
| ZINC22912016 | -6.9 | -0.493 | -5.5 | -0.393 | -5.3 | -0.379 | -7 | -0.5 |
| ZINC22918614 | -6.2 | -0.238 | -6.7 | -0.258 | -6.2 | -0.238 | -5.5 | -0.212 |
| ZINC23118752 | -8.2 | -0.304 | -7.2 | -0.267 | -7 | -0.259 | -6.9 | -0.256 |
| ZINC23118754 | -9 | -0.333 | -7.1 | -0.263 | -6.7 | -0.248 | -6.9 | -0.256 |
| ZINC23118770 | -8.8 | -0.314 | -7.5 | -0.268 | -6.3 | -0.225 | -6.6 | -0.236 |
| ZINC23118772 | -9.4 | -0.336 | -6.9 | -0.246 | -6.7 | -0.239 | -6.6 | -0.236 |
| ZINC23211906 | -7.7 | -0.308 | -6.5 | -0.26 | -6.2 | -0.248 | -6.1 | -0.244 |
| ZINC23211908 | -8.3 | -0.332 | -6.3 | -0.252 | -6.2 | -0.248 | -6.1 | -0.244 |
| ZINC24334040 | -7.3 | -0.317 | -6.3 | -0.274 | -5.8 | -0.252 | -5.7 | -0.248 |
| ZINC25783872 | -7.2 | -0.195 | -7.4 | -0.2 | -6.3 | -0.17 | -7 | -0.189 |
| ZINC26730911_1 | -8.2 | -0.265 | -8.1 | -0.261 | -7.3 | -0.235 | -7.3 | -0.235 |
| ZINC26730911 | -7.7 | -0.248 | -8.1 | -0.261 | -7.2 | -0.232 | -7.2 | -0.232 |
| ZINC26780933 | -7.9 | -0.304 | -6.5 | -0.25 | -6 | -0.231 | -6.1 | -0.235 |
| ZINC26780935 | -7.6 | -0.292 | -6.5 | -0.25 | -6.3 | -0.242 | -6 | -0.231 |
| ZINC26780937 | -7.8 | -0.3 | -6.4 | -0.246 | -5.7 | -0.219 | -6 | -0.231 |
| ZINC26780939 | -7.5 | -0.288 | -6 | -0.231 | -6.1 | -0.235 | -5.5 | -0.212 |
| ZINC29589543 | -7.2 | -0.3 | -7.8 | -0.325 | -6.4 | -0.267 | -6.2 | -0.258 |
| ZINC29589553 | -7.9 | -0.439 | -6.3 | -0.35 | -5.8 | -0.322 | -5.7 | -0.317 |
| ZINC29589777 | -7.6 | -0.362 | -6.8 | -0.324 | -6.3 | -0.3 | -6.2 | -0.295 |
| ZINC29589785 | -5.1 | -0.51 | -4.5 | -0.45 | -4.5 | -0.45 | -4.7 | -0.47 |
| ZINC29589797 | -7.5 | -0.375 | -6.4 | -0.32 | -6.6 | -0.33 | -6 | -0.3 |
| ZINC29589800_1 | -5.4 | -0.491 | -4.5 | -0.409 | -4.4 | -0.4 | -4.8 | -0.436 |
| ZINC29589800 | -5.4 | -0.491 | -4.5 | -0.409 | -4.4 | -0.4 | -4.8 | -0.436 |
| ZINC29589816 | -7.8 | -0.312 | -6.8 | -0.272 | -6.5 | -0.26 | -6.1 | -0.244 |
| ZINC29589818 | -8.2 | -0.328 | -6.6 | -0.264 | -6.1 | -0.244 | -5.9 | -0.236 |
| ZINC29589820 | -7.8 | -0.312 | -6.6 | -0.264 | -6.4 | -0.256 | -6.1 | -0.244 |
| ZINC29589822 | -7.8 | -0.312 | -6.5 | -0.26 | -6.5 | -0.26 | -6 | -0.24 |
| ZINC29589824 | -7.4 | -0.296 | -6.6 | -0.264 | -6.4 | -0.256 | -6.5 | -0.26 |
| ZINC29589826 | -8 | -0.32 | -6.7 | -0.268 | -6.7 | -0.268 | -6.7 | -0.268 |
| ZINC29589828 | -8.4 | -0.3 | -7.2 | -0.257 | -7 | -0.25 | -6.1 | -0.218 |
| ZINC29589829 | -9.3 | -0.332 | -7.3 | -0.261 | -6.8 | -0.243 | -6.6 | -0.236 |
| ZINC29589831 | -8.1 | -0.289 | -6.9 | -0.246 | -6.7 | -0.239 | -6.6 | -0.236 |
| ZINC29589833 | -8.5 | -0.304 | -6.9 | -0.246 | -6.2 | -0.221 | -6.7 | -0.239 |
| ZINC29589835 | -8.3 | -0.296 | -7.4 | -0.264 | -6.9 | -0.246 | -6.7 | -0.239 |
| ZINC29589837 | -9.1 | -0.325 | -7 | -0.25 | -6.9 | -0.246 | -7 | -0.25 |
| ZINC29589855 | -7.4 | -0.285 | -7.7 | -0.296 | -6.3 | -0.242 | -6.3 | -0.242 |
| ZINC29589859 | -8 | -0.471 | -6.2 | -0.365 | -6.1 | -0.359 | -6.1 | -0.359 |
| ZINC29589876 | -7.3 | -0.365 | -6.9 | -0.345 | -5.9 | -0.295 | -5.9 | -0.295 |
| ZINC29589877 | -6.7 | -0.335 | -6.8 | -0.34 | -6 | -0.3 | -6.2 | -0.31 |
| ZINC29589879 | -7.3 | -0.365 | -7 | -0.35 | -6 | -0.3 | -6 | -0.3 |
| ZINC29589881 | -6.6 | -0.244 | -6.4 | -0.237 | -6.1 | -0.226 | -5.6 | -0.207 |
| ZINC29589883 | -7 | -0.292 | -7 | -0.292 | -6.9 | -0.288 | -6.8 | -0.283 |
| ZINC29589885 | -7.9 | -0.329 | -7.1 | -0.296 | -7 | -0.292 | -7.1 | -0.296 |
| ZINC29589888 | -8.8 | -0.267 | -8.7 | -0.264 | -8.1 | -0.245 | -7.3 | -0.221 |
| ZINC29589897 | -8.5 | -0.472 | -6.1 | -0.339 | -5.8 | -0.322 | -5.6 | -0.311 |
| ZINC29589899 | -7.2 | -0.343 | -6.6 | -0.314 | -6.5 | -0.31 | -6 | -0.286 |
| ZINC29589901 | -7.2 | -0.343 | -6.8 | -0.324 | -6.3 | -0.3 | -6.2 | -0.295 |
| ZINC29589906 | -5.2 | -0.578 | -4.4 | -0.489 | -4.1 | -0.456 | -4.7 | -0.522 |
| ZINC29589907_1 | -6.4 | -0.4 | -5.7 | -0.356 | -5.4 | -0.338 | -5 | -0.312 |
| ZINC29589907 | -6.2 | -0.388 | -5.9 | -0.369 | -5.4 | -0.338 | -5 | -0.312 |
| ZINC29589912 | -6.4 | -0.267 | -6.6 | -0.275 | -5.8 | -0.242 | -5.8 | -0.242 |
| ZINC29589917 | -7 | -0.333 | -7.3 | -0.348 | -6.2 | -0.295 | -6.5 | -0.31 |
| ZINC29589923 | -7.4 | -0.37 | -5.6 | -0.28 | -6.3 | -0.315 | -5.1 | -0.255 |
| ZINC29589924 | -5.7 | -0.438 | -4.5 | -0.346 | -4.3 | -0.331 | -4.4 | -0.338 |
| ZINC29590257 | -7.9 | -0.161 | -8.5 | -0.173 | -8.3 | -0.169 | -8.4 | -0.171 |
| ZINC29590259 | -8.3 | -0.169 | -7.8 | -0.159 | -8.3 | -0.169 | -8.4 | -0.171 |
| ZINC29590262 | -8 | -0.163 | -8.4 | -0.171 | -8 | -0.163 | -8.2 | -0.167 |
| ZINC29590263 | -8.3 | -0.169 | -8.2 | -0.167 | -8.6 | -0.176 | -8.4 | -0.171 |
| ZINC29590275 | -9.1 | -0.294 | -7.9 | -0.255 | -7.7 | -0.248 | -7.4 | -0.239 |
| ZINC29590277 | -7.3 | -0.235 | -7.5 | -0.242 | -7.3 | -0.235 | -7 | -0.226 |
| ZINC29590287 | -6.7 | -0.479 | -5.3 | -0.379 | -5.2 | -0.371 | -5.2 | -0.371 |
| ZINC29590289_1 | -6.8 | -0.378 | -5.2 | -0.289 | -5.1 | -0.283 | -5 | -0.278 |
| ZINC29590289 | -6.9 | -0.383 | -5.2 | -0.289 | -5 | -0.278 | -5.1 | -0.283 |
| ZINC29590292_1 | -8.4 | -0.336 | -7.4 | -0.296 | -6.9 | -0.276 | -5.7 | -0.228 |
| ZINC29590292 | -8.9 | -0.356 | -7.3 | -0.292 | -6.8 | -0.272 | -6 | -0.24 |
| ZINC29590296 | -7.8 | -0.325 | -6.5 | -0.271 | -6.5 | -0.271 | -6.4 | -0.267 |
| ZINC29590298 | -8.3 | -0.346 | -6.6 | -0.275 | -6.5 | -0.271 | -6.2 | -0.258 |
| ZINC29590300 | -8.1 | -0.324 | -6.5 | -0.26 | -6.7 | -0.268 | -6.5 | -0.26 |
| ZINC29590302 | -8.8 | -0.352 | -6.7 | -0.268 | -6.6 | -0.264 | -6.6 | -0.264 |
| ZINC29590304 | -8.5 | -0.37 | -7.2 | -0.313 | -6.7 | -0.291 | -6.4 | -0.278 |
| ZINC29590306 | -8.7 | -0.378 | -6.9 | -0.3 | -6.5 | -0.283 | -6.6 | -0.287 |
| ZINC29590308 | -7.5 | -0.395 | -5.9 | -0.311 | -5.9 | -0.311 | -5.3 | -0.279 |
| ZINC29590315 | -6.2 | -0.238 | -6.9 | -0.265 | -6.1 | -0.235 | -5.9 | -0.227 |
| ZINC29590323_1 | -5.7 | -0.475 | -4.4 | -0.367 | -4.3 | -0.358 | -5.2 | -0.433 |
| ZINC29590323 | -5.7 | -0.475 | -4.2 | -0.35 | -4.1 | -0.342 | -5.2 | -0.433 |
